# KMT5A-methylated SNIP1 promotes triple-negative breast cancer metastasis by activating YAP signaling

**DOI:** 10.1038/s41467-022-29899-w

**Published:** 2022-04-21

**Authors:** Bo Yu, Jun Su, Qiqi Shi, Qing Liu, Jun Ma, Guoqing Ru, Lei Zhang, Jian Zhang, Xichun Hu, Jianming Tang

**Affiliations:** 1 Department of Medical Oncology, Fudan University Shanghai Cancer Center, Shanghai, 200032 P. R. China; 2 Department of Oncology, Shanghai Medical College, Fudan University, Shanghai, 200032 P. R. China; 3 Department of Oncology, Xinhua Hospital Affiliated to Shanghai Jiaotong University School of Medicine, Shanghai, 200025 P. R. China; 4 State Key Laboratory of Oncogenes and Related Genes, Renji-Med X Clinical Stem Cell Research Center, Ren Ji Hospital, Shanghai Cancer Institute, School of Medicine, Shanghai Jiao Tong University, Shanghai, 200127 P. R. China; 5 Department of Medical Oncology, Zhongshan Hospital, Fudan University, Shanghai, 200032 P. R. China; 6 Eye Institute, Eye & ENT Hospital, Shanghai Medical College, Fudan University, Shanghai, 200032 P. R. China; 7 Department of Pathology, Zhejiang Provincial People’s Hospital, People’ s Hospital of Hangzhou Medical College, Hangzhou, Zhejiang 310014 P. R. China; 8 Department of Radiation Oncology, Renji Hospital, School of Medicine, Shanghai Jiao Tong University, Shanghai, 200127 P. R. China; 9 Department of Phase I Clinical Trial Center, Fudan University Shanghai Cancer Center, No. 270, Dong’an Road, Shanghai, 200032 China; 10 Institute of Cancer Neuroscience, Medical Frontier Innovation Research Center, The First Hospital of Lanzhou University, The First Clinical Medical College of Lanzhou University, Lanzhou, 730000 P. R. China

**Keywords:** Breast cancer, Methylation, Methylation

## Abstract

Smad nuclear-interacting protein 1 (SNIP1) is a transcription repressor related to the TGF-β signaling pathway and associates with c-MYC, a key regulator of cell proliferation and tumor development. Currently, the mechanism by which SNIP1 regulates tumorigenesis and cancer metastasis is unknown. Here, we identify that SNIP1 is a non-histone substrate of lysine methyltransferase KMT5A, which undergoes KMT5A-mediated mono-methylation to promote breast cancer cell growth, invasion and lung metastasis. Mechanistically, we show KMT5A-mediated K301 methylation of SNIP1 represents a sensing signal to release histone acetyltransferase KAT2A and promotes the interaction of c-MYC and KAT2A, and the recruitment of c-MYC/KAT2A complex to promoter of c-MYC targets. This event ultimately inhibits the Hippo kinase cascade to enhance triple-negative breast cancer (TNBC) metastasis by transcriptionally activating MARK4. Co-inhibition of KMT5A catalytic activity and YAP in TNBC xenograft-bearing animals attenuates breast cancer metastasis and increases survival. Collectively, this study presents an KMT5A methylation-dependent regulatory mechanism governing oncogenic function of SNIP1.

## Abstract (web summary)

SNIP1 methylation initiates its oncogenic functions. Here, the authors show that SNIP1 is methylated by KMT5A and this leads to downstream signalling that activates the YAP pathway, resulting in tumorigenesis and metastasis.

## Introduction

Breast cancer is the most prevalently diagnosed cancer type and a lethal primary malignant tumor among women across the globe^1,2^. Triple-negative breast cancers (TNBCs) account for ~15% of all invasive breast cancers; they are insensitive to endocrine-targeted therapy due to lack of receptors^3^. Despite an initial response to systemic chemotherapy, increased risk of recurrence, metastasis, and mortality have been reported in patients diagnosed with TNBCs^4^. Therefore, there is an urgent need for a better understanding of the aggressive phenotype and higher metastatic potential, as well as identifying molecular targets.

Smad nuclear interacting protein 1 (SNIP1) is a nuclear protein cloned and characterized based on its ability to inhibit the TGF-β signal transduction pathway^5^. It is expressed in various cell types, including the brain, placenta, and kidney. SNIP1 is also upregulated in a wide range of tumors and associated with cell transformation and DNA damage response^6,7^. Several functions have been identified to SNIP1, suggesting its ability to interact with multiple cellular partners; notably, the majority of these functions described so far involve transcriptional regulation^8^. The N-terminal nuclear localization signal (NLS) domain of SNIP1 binds the C/H1 domain of transcriptional coactivators CBP and p300, competing for the binding of Smad4 and p65/RelA to CBP/p300^9^. SNIP1 overexpression inhibits Smad4 and p65/RelA transactivation by preventing their interaction with p300 through its NLS domain^9^. Conversely, SNIP1 also enhances the transcriptional activity of c-Myc by stabilizing it against proteasomal degradation and bridging the c-Myc/p300 complex, binding the N terminus of c-Myc through its own C terminus^7^. Therefore, SNIP1 might be an important endogenous modulator of multiple transcriptional pathways requiring transcriptional coactivators CBP/p300.

Accumulating evidence reveals that the epigenetic landscape is dysregulated in breast cancer, including TNBC, partially due to the aberrant patterns of posttranslational modifications (PTMs)^10–12^. Lysine methylation has evolved as a vital PTM implicated in various biological functions^13^. Further, lysine methylation of non-histone proteins regulates numerous molecular events including protein-protein interaction, protein stability, protein subcellular localization, and transcription^14–16^. Mechanistically, lysine methylation of non-histone proteins facilitates their interactions with specific readers for downstream signaling; besides, the crosstalk between lysine methylation and other PTMs on non-histone proteins impacts the biological functions of modified proteins^17,18^. Lysine methylation is primarily catalyzed by a family of protein methyltransferases (KMTs), including seven major families namely SUV39, SET1, STE2, RIZ, SMYD, EZ, and SUV4-20^19^. Although a significant number of KMTs have been detected in the human genome, only a few non-histone proteins including YAP, p53, STAT1, and AKT, undergo lysine methylation^20–23^. This indicates that a considerable number of lysine methylated species remain unidentified. Moreover, whether SNIP1 methylation occurs, and regulates oncogenic signaling, as well as is involved in TNBC cancer metastasis, remains to be investigated.

This work uses mass spectrometry (MS) analysis to identify KMT5A (also known as PR-Set7/9, SETD8, and SET8) as a SNIP1-interacting protein that methylates non-histone SNIP1 at K301. Consequently, KMT5A-mediated K301 methylation of SNIP1 releases KAT2A as well as promotes c-MYC/KAT2A complex formation and transcriptional activation of c-MYC targets. Subsequently, methylation of SNIP1 by KMT5A enhances key oncogenic pathway-Hippo signaling, thereby promoting TNBC metastasis. As such, our data recognize KMT5A-mediated SNIP1 methylation as an essential step for c-MYC/KAT2A complex formation and c-MYC target activation. In addition, our findings further highlight the histone methyltransferase KMT5A as a potential therapeutic target for hyperactive SNIP1-driven TNBC metastasis.

## Results

### SNIP1 methylation promotes its oncogenic functions

**Fig. 1:**
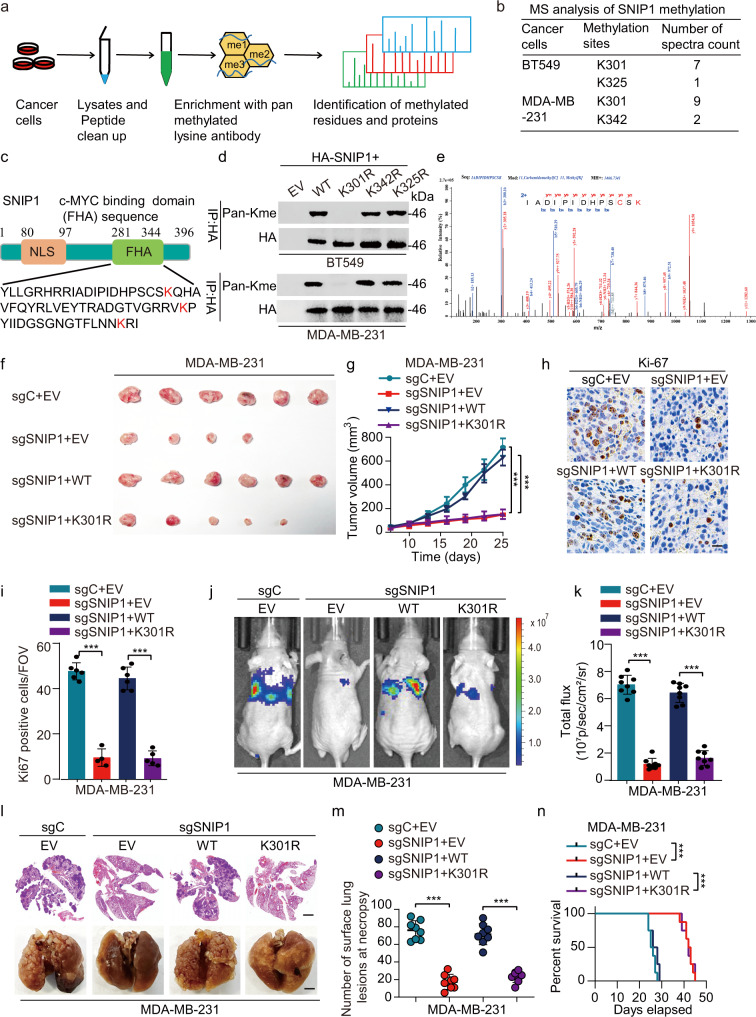
SNIP1 methylation promotes its oncogenic functions. **a** A schematic workflow of IAP LC-MS/MS experiments. BT549 and MDA-MB-231 cell lysates were digested to perform IAP LC–MS/MS assays. **b** MS analysis showing potential methylation sites in SNIP1 after the immunoprecipitation (IP) of SNIP1 in BT549 and MDA-MB-231 cells. **c** Schematic representation of SNIP1 showing the c-MYC binding domain (FHA) and its amino acid sequence with all lysine (K) residues highlighted in red. NLS, nuclear localization signal. FHA, Forkhead associated domain. **d** Immunoblot (IB) analysis of the lysine methylation of wildtype (WT) and KR mutant SNIP1 in MDA-MB-231 and BT549 cells transfected with the indicated plasmids; lysates were assessed by IP with anti-HA and IB with anti-Pan-Me-Lys and anti-HA (*n* = 3). **e** LC-MS/MS spectrum of the tryptic peptide IADIPIDHPSCSK identified a monomethylated residue at K301, carrying a mass of +14.016 Da. **f**, **g** Cells derived from MDA-MB-231/sgSNIP1 cells re-overexpressed with indicated SNIP1-WT or SNIP1-K301R and selected with hygromycin (200 μg/ml) for 72 h before the collection was subjected to mouse xenograft assays by orthotopic injection. Tumor sizes were monitored and analyzed (**f**, **g**) (*n*  =  6 mice per group). **h** Representative photos of Ki-67 positive staining cells in murine primary tumor sections of indicated tumor tissues. Scale bar, 100 μm (IHC). **i** Quantification of the number of cells positive for Ki-67 staining per field of vision in tumor sections from (**h**) (*n* = 6 for each group). FOV field of view. **j**–**m** Cells generated in (**f**) were subjected to mouse xenograft through tail vein injection. Lung metastases were monitored (**j**, **l**) and calculated (**k**, **m**) (*n*  =  8 mice per group). **n** Kaplan–Meier survival of mice in (**j**) (*n*  =  8 mice per group). Data information: In (**g**, **i**, **k**, **m**), statistical analysis was performed by a two-tailed *t*-test. In (**n**), by log-rank test. ***P < 0.001. Data are represented as mean  ± SEM. Panels (**d**) and (**f**–**n**) show one experiment representative of three independent experiments with similar results.

To identify important non-histone proteins regulated in a methylation-dependent manner, a specific pan-lysine methylation antibody was used to perform a mass spectrometry (MS)-based screening of cell lysates derived from two TNBC cell lines, i.e., BT549 and MDA-MB-231 (Fig. 1a). Notably, several SNIP1-derived peptides were identified, comprising methylated modifications at three nearby evolutionarily conserved lysine residues (K301, K325, and K342) in the FHA (281-344aa) domain (Fig. 1b, c). Notably, the K301 methylation was detected in both two cell lines. Then, the SNIP1 mutant was generated by replacing the above lysine residues in the FHA domain with arginine. This lysine (K)-to-arginine (R) substitution prevents methylation but maintains a positive charge, thereby mimicking the non-methylated form of a protein. Interestingly, only the SNIP1 K301R mutant, but not the other mutants, was not methylated (Fig. 1d), suggesting Lys301 as the SNIP1 methylation site in the FHA. And LC-MS/MS revealed that K301 was mono-methylated (Fig. 1e). Furthermore, SNIP1 mono-methylation was confirmed using Kme1-specific antibody in cells treated with a global histone methylation inhibitor, 3-deazaneplanocin A (Supplementary Fig. 1a). Moreover, the Lys301 site was highly and evolutionarily conserved in SNIP1 proteins among different species (Supplementary Fig. 1b), suggesting the functional specificity of lysine in SNIP1. Taken together, these findings indicate that SNIP1 is methylated at Lys301.

Given that the K301 site was identified at endogenous levels through a large-scale non-biased MS approach, this paper investigated the contribution of K301 mono-methylation to SNIP1 activity and its oncogenic functions. To reveal the potential biological functions of mono-methylation within its FHA region, a methylation deficient variant of SNIP1 (K301R) was ectopically re-expressed in SNIP1 knockout MDA-MB-231 and BT549 cell lines (MDA-MB-231/sgSNIP1 and BT549/sgSNIP1) (Supplementary Fig. 1c). Furthermore, a rabbit polyclonal antibody specifically recognizing the K301 mono-methylated SNIP1 (Supplementary Fig. 1c–e) was generated through a commercial vendor. Dot blot analysis was performed to demonstrate a minimal cross-reactivity of the polyclonal antibody with un-methylated K301 containing peptide (Supplementary Fig. 1f). Also, its specificity for SNIP1-K301me1 was verified in MDA-MB-231 cells and a clinical breast cancer specimen (Supplementary Fig. 1g, h).

Then in contrast with the control (sgC), the knockout of SNIP1inhibited the colony formation in vitro (Supplementary Fig. 1i, j), and reduced tumor growth in vivo (Fig. 1f, g) with significantly downregulated Ki67 expression in primary tumor tissues (Fig. 1h, i); while re-expression of wild-type SNIP1 (SNIP1^WT^) but not the SNIP1^K301R^ mutant significantly restored the ability of cell colony formation (Supplementary Fig. 1i, j), tumor growth (Fig. 1f, g) and Ki67 expression (Fig. 1h, i). Moreover, SNIP1 knockout exhibited significantly inhibited the invasiveness of breast cancer cells in vitro (Supplementary Fig. 1k, l), tumor metastasis to the lungs and metastatic lung nodule formation (Fig. 1j–m), and prolonged animal survival (Fig. 1n); similarly, re-expression of SNIP1^WT^ but not the SNIP1^K301R^ mutant restored the ability of cell invasion (Supplementary Fig. 1k, l), tumor lung metastasis (Fig. 1j–m), and shortened animal survival (Fig. 1n). Together, these findings confirm that SNIP1 methylation potentially promotes TNBC growth and metastasis both in vitro and in vivo.

### SNIP1 interacted with and was methylated at Lys301 by KMT5A

**Fig. 2:**
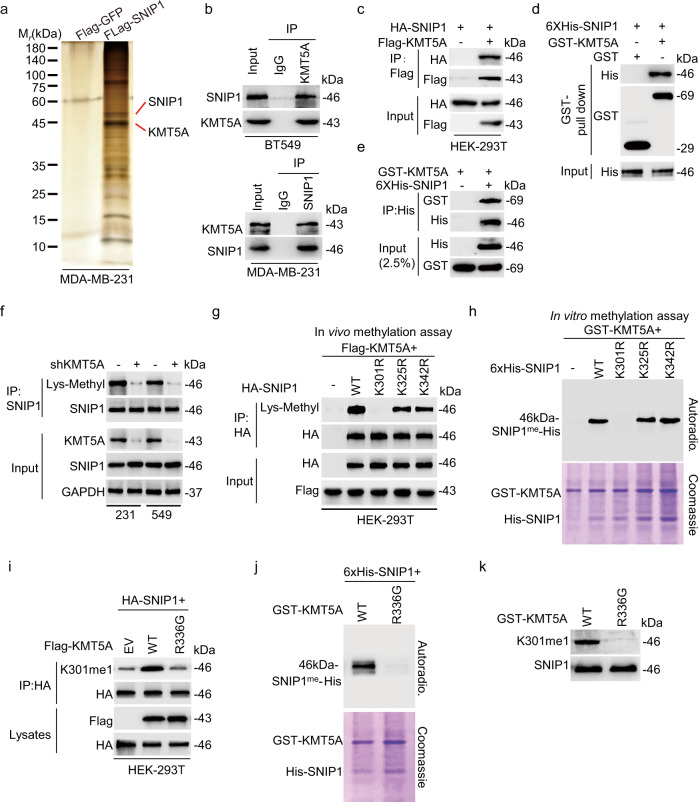
SNIP1 interacted with and was methylated at Lys301 by KMT5A. **a** Immunoprecipitation followed by silver staining of the SNIP1 binding proteins. **b** Co-immunoprecipitation of endogenous SNIP1 with anti KMT5A antibody in BT549 (upper) and endogenous KMT5A with anti SNIP1 antibody (lower) in MDA-MB-231 cells (*n* = 3). **c** Immunoprecipitation and western blotting for Flag-KMT5A association with HA-SNIP1 protein in HEK-293T cells (*n* = 3). **d**, **e** GST pulldown with purified GST-KMT5A (**d**) and Ni-NTA pulldown with 6X His-SNIP1 (**e**) followed by immunoblotting with anti-His and GST antibodies (*n* = 3). **f** Immunoblot analysis of the lysine methylation of SNIP1 in control and KMT5A-knockdown MDA-MB-231 and BT549 cells. Lysates were assessed by immunoprecipitation (IP) with anti-SNIP1 and immunoblotting with anti-Lys-Methyl. **g**, In vivo methylation assay for the detection of SNIP1 methylation by western blotting using anti-Lys-Methyl. HA-tagged SNIP1^WT^, SNIP1^K301R^, SNIP1^K325R^, and SNIP1^K342R^ mutants were immunoprecipitated with anti-HA magnetic beads from HEK-293T cells co-expressed with Flag-KMT5A (*n* = 3). **h** Autoradiography of the in vitro methylation assay using purified GST-KMT5A and 6× His-SNIP1. Top panel, 3H-SAM is the methyl donor, and methylation visualized by autoradiography. Bottom panel, total amounts of GST-KMT5A and 6× His-SNIP1 are shown by Coomassie brilliant blue (C.B.B.) staining. Empty vector (EV). Wild-type (WT) (*n* = 3). **i** Effects of wild type and methyltransferase activity-deficient mutant R336G of KMT5A on SNIP1-K301 methylation (*n* = 3). **j** Autoradiography of the in vitro methylation assay using purified GST-KMT5A^WT^ or methyltransferase activity-deficient mutant GST-KMT5A^R336G^ and 6× His-SNIP1. Top panel, 3H-SAM is the methyl donor and methylation visualized by autoradiography. Bottom panel, total amounts of GST-KMT5A^WT^ or -KMT5A^R336G^ and 6× His-SNIP1 are shown by Coomassie brilliant blue (C.B.B.) staining. Wild-type (WT) (*n* = 3). **k** In vitro methylation assay was performed with wild-type and mutant KMT5A on recombinant SNIP1 and probed with anti-K301me1 (*n* = 3). Data information: Panels (**b**–**k**) show 1 experiment representative of three independent experiments with similar results.

To identify the physiological upstream methyltransferase (or methyltransferases) that mediates SNIP1 K301 mono-methylation, a co-immunoprecipitation (co-IP) assay and LC-MS/MS were conducted to capture the SNIP1-interacting proteins from MDA-MB-231 cells. As a result, methyltransferase KMT5A was the only lysine methyltransferase among the top SNIP1-interacting proteins based on the abundance ratio above 10 and *P* < 0.05 (Fig. 2a). This suggests that KMT5A potentially methylates SNIP1. We indeed observed a relatively strong physical interaction between endogenous SNIP1 and KMT5A in MDA-MB-231 and BT549 cells (Fig. 2b). Subsequently, HA-SNIP1 was also detected in the anti-Flag-KMT5A immunoprecipitates of HEK-293T cells transfected with indicated plasmids (Fig. 2c). Further, His-tagged SNIP1 was pulled down by GST-KMT5A and vice versa, indicating a direct physical interaction between SNIP1 and KMT5A (Fig. 2d, e). To determine the SNIP1 domain that binds to KMT5A, a deletion-mapping approach was adopted (Supplementary Fig. 2a, b); then these deletion mutant protein isoforms were transduced into HEK-293T cells. As shown in Supplementary Fig. 2a, FL, N1, and C mutants of SNIP1, but not the N2 mutant bound to KMT5A, indicating that the SNIP1 FHA (281-344 aa) domain directly interacted with the KMT5A protein. Moreover, FL and C mutants of KMT5A, but not the N mutant bound to SNIP1 (Supplementary Fig. 2b), suggesting that the SET (216-343 aa) domain of KMT5A was also directly bound to the SNIP1 protein. These results indicate that SNIP1 directly interacts with KMT5A.

The physical interaction between SNIP1 and KMT5A suggests a potential methylation regulation. Indeed, the methyl lysine (K) levels of SNIP1 were substantially impaired in both MDA-MB-231 and BT549 cells with KMT5A knockdown, as established by the anti-pan methyl antibody (Fig. 2f). Considering SNIP1 was mono-methylated at Lys301 which located in FHA domain bound to KMT5A, K301 was further predicted for KMT5A methylation site. A mutant SNIP1 was generated using arginine (R) to replace the K301 residue and two other lysine residues (K325 and K342) in the FHA domain. As shown in Fig. 2g, in vivo methylation analysis confirmed that only the replacement of K301 with R had a significant effect on SNIP1 lysine methylation by KMT5A when examined by an anti-pan-methylation antibody. Furthermore, in vitro methylation analysis demonstrated that only K301R (but not K325R and K342R), showed a decrease in methylation signals (Fig. 2h). This indicates that KMT5A acts as a putative SNIP1 methyltransferase and potentially responsible for K301 methylation on SNIP1.

To further confirm this observation, the wild-type (KMT5A^WT^) or catalytic inactive mutant of KMT5A (KMT5A^R336G^) was co-transfected with HA-SNIP1 into HEK-293T cells. Consequently, unlike KMT5A^WT^, KMT5A^R336G^ sharply abolished mono-methyl K301 (K301me1) levels of HA-SNIP1 (Fig. 2i). Comparable findings were observed in another independent in vitro methylation analysis (Fig. 2j). This methylation was further confirmed in tests with the anti-SNIP1 K301me1 antibody (Fig. 2k). These results confirm a direct interaction between KMT5A and SNIP1, causing a mono-methylation of lysine 301 in SNIP1 by KMT5A.

### KMT5A mediated SNIP1 methylation activates Hippo/YAP signaling

**Fig. 3:**
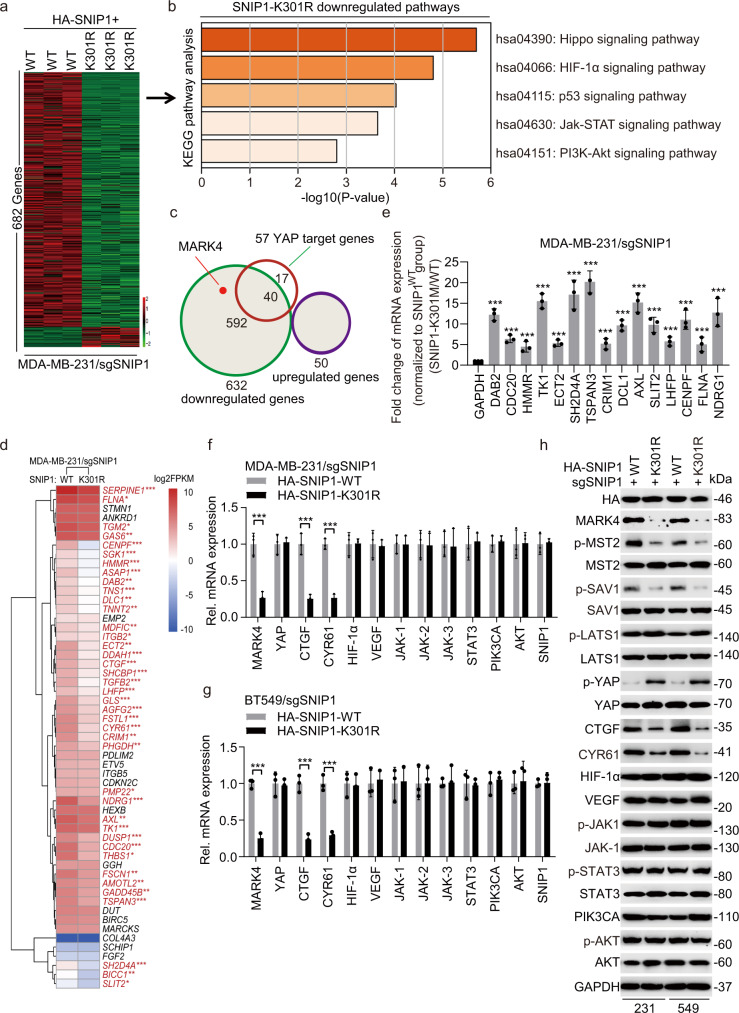
KMT5A-mediated SNIP1 K301 methylation activates Hippo/YAP signaling. **a** Heatmap of RNA-Seq analysis of differentially expressed genes downregulated by SNIP1-K301R (SNIP1^K301R^) (twofold change and FDR < 0.05). SNIP1 sgRNA-resistant Flag-SNIP1^WT^ and SNIP1^K301R^ mutant were re-expressed in sgSNIP1/MDA-MB-231 cells. **b** Kyoto Encyclopedia of Genes and Genomes (KEGG) analysis indicates that the genes downregulated by the SNIP1^K301R^ mutant were predominantly associated with the Hippo signaling pathway. **c** Venn diagram shows YAP target genes that are regulated by SNIP1 demethylation (SNIP1^K301R^). **d** Heatmap representing expression levels of 55 YAP target genes determined by RNA-Seq in (**a**). Forty genes were found to be significantly downregulated in K301R mutant cell lines were highlighted in red. **e** qPCR analysis of a group of YAP target genes that were induced upon SNIP1 K301 methylation (SNIP1^K301M^) (*n* = 3). **f**, **g** these representative typical pathway genes that may be regulated by SNIP1 methylation were measured and verified in MDA-MB-231 (**f**) and BT549 (**g**) cells by qRT-PCR (*n* = 3). **h** Western blot was performed in MDA-MB-231/sgSNIP1 and BT549/sgSNIP1 cells transfected with SNIP1 sgRNA-resistant Flag-SNIP1^WT^ and SNIP1^K301R^ mutant (*n* = 3). Data information: In (**d**, **e**–**g**), statistical analysis was performed by a two-tailed *t*-test. mean ± SEM. *P < 0.05, **P < 0.01, ***P < 0.001. Panels (**e**–**h**) show 1 experiment representative of three independent experiments with similar results.

To elucidate the functional significance of SNIP1 methylation, RNA sequencing analysis was performed to compare gene expression profiles in MDA-MB-231/sgSNIP1 cells with re-expression of sgRNA-resistant HA-SNIP1^WT^ or HA-SNIP1^K301R^ mutant. A total of 632 downregulated and 50 upregulated genes were identified (raw data accessible via Bioproject ID: PRJNA797682) by re-expressing sgRNA-resistant SNIP1^K301R^ mutant compared to the SNIP1^WT^ control group (fold change >2, *P* < 0.05) (Fig. 3a). KEGG pathway analysis of 632 down-regulated genes identified the Hippo signaling pathway as the most significant pathway (Fig. 3b and Supplementary data 1). Previous studies identified 57 YAP target genes in various cell types including breast cancer cells^24,25^. Among these, 40 genes, also identified as YAP-regulated downstream genes, were downregulated in MDA-MB-231/sgSNIP1 cells with re-expressing sgRNA-resistant SNIP1^K301R^ mutant compared to the SNIP1^WT^ group (Fig. 3c, d). Conversely, YAP target gene levels were significantly increased in MDA-MB-231/sgSNIP1 cells with re-expressing sgRNA-resistant SNIP1 K301 methylation mimics mutant (SNIP1^K301M^) compared to the SNIP1^WT^ group (Fig. 3e).

Interestingly, as shown in Fig. 3c, SNIP1^K301R^ also downregulated the expression of MAP/microtubule affinity-regulating kinase 4 (MARK4), which could phosphorylate the Hippo core components MST1/2 or SAV; besides, it disrupted a complex formation between MST/SAV and LATS to influence YAP/TAZ activity, which as a potent activator of YAP/TAZ activity, MARK4 inhibits YAP/TAZ phosphorylation to promote YAP/TAZ nuclear localization, ultimately inducing transcriptional activation in breast cancer^26^. To investigate the possibility of SNIP1 methylation mediating YAP target genes by the Hippo kinase cascade MARK4/MST/SAV/YAP-mediated transcriptional activation, re-expression of SNIP1^K301R^ significantly downregulated MARK4, p-MST2, p-SAV, upregulated p-YAP and slightly increased p-LATS1 expression levels. Besides, it effectively altered the Hippo kinase cascade and target genes of the Hippo/YAP signaling pathway, i.e., CTGF and CYR61^27^ in MDA-MB-231 and BT549 cells; excluding the typical genes including HIF-1α, VEGF, p-JAK1, JAK1, p-STAT3, STAT3, PI3KCA, p-AKT and AKT levels implicated in the major pathways of KEGG pathway analysis (Fig. 3f–h). These data indicate that SNIP1 K301 methylation modulates the Hippo/YAP signaling pathway by regulating MARK4 expression and subsequently modifying phosphorylation of MST2, SAV, LATS1, and YAP, but not other components.

### KMT5A-mediated SNIP1 K301 methylation promotes TNBC metastasis by activating YAP signaling

KMT5A-mediated SNIP1 K301 methylation positively regulates the Hippo/YAP pathway, and numerous studies have firmly established the role of Hippo/YAP during tumorigenesis and metastasis^28^. We hypothesized that SNIP1 K301 methylation modulates YAP signaling to promote TNBC metastasis in a KMT5A-dependent manner. First, an immunohistochemical staining analysis was performed in 100 clinical TNBC primary tumors and matched metastatic lymph nodes to gain insight into the role of KMT5A in TNBC metastasis. As a result, KMT5A was more highly expressed in matched metastatic lymph nodes than in the primary tumor (Supplementary Fig. 3a, b). Moreover, TNBC patients with high KMT5A levels had a significantly worse prognosis than those with low KMT5A expression using Kaplan–Meier survival analysis (Supplementary Fig. 3c). In addition, unlike the normal breast epithelium cell line MCF-10A cells, KMT5A expression was significantly higher in all breast cancer cells. Furthermore, the metastatic breast cancer cell lines (4T1, BT549, and MDA-MB-231) expressed higher levels of KMT5A than those of non-metastatic breast cancer cell lines (MDA-MB-453, MCF7, T47D, and BT474) (Supplementary Fig. 3d). These data suggest that high expression of KMT5A is linked to a metastatic phenotype, indicating a poor prognosis.

**Fig. 4:**
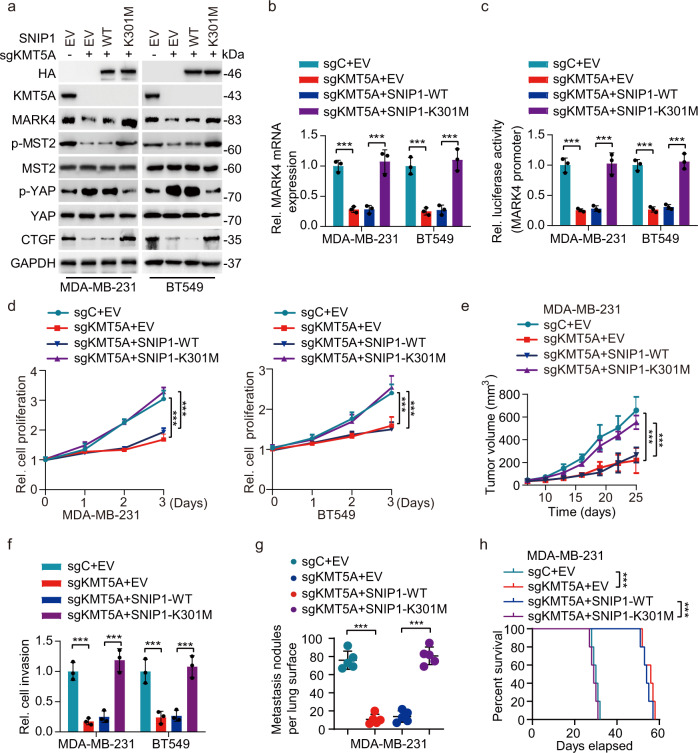
KMT5A-mediated SNIP1 K301 methylation promotes TNBC metastasis via activating YAP signaling. **a** Western blotting for ectopic expression of SNIP1^WT^ or SNIP1^K301M^ mutant combined with KMT5A knockout on MARK4 protein expression and Hippo signaling activation in MDA-MB-231 and BT549 cells. Empty vector (EV), Wild-type (WT) (*n* = 3). **b**, **c** Effects of ectopic expression of SNIP1^WT^ or SNIP1^K301M^ mutant combined with KMT5A knockout on MARK4 mRNA expression (**b**) and luciferase activity from MARK4 promoter fragments (**c**). Nontargeting sgRNA (sgC) (*n* = 3). **d** Effects of ectopic expression of SNIP1^WT^ or SNIP1^K301M^ mutant combined with KMT5A knockout on MDA-MB-231 and BT549 cells proliferation (*n* = 3). **e** Cells generated in (**d**) were subjected to mouse xenograft assays by orthotopic injection in athymic nude mice, and tumor sizes were monitored and analyzed. Data represent the mean ± SEM (*n* = 5 mice per group). **f**–**h** Effects of ectopic expression of SNIP1^WT^ or SNIP1^K301M^ mutant combined with KMT5A knockout on cell invasion (**f**) (*n* = 3), lung metastasis (**g**), and mouse lifespan (**h**). 1 × 10^5^ MDA-MB-231 breast cancer cells transduced with control sgRNA (sgC) or sgKMT5A, with or without ectopic expression of SNIP1^WT^ or SNIP1^K301M^ mutant were implanted into the lateral tail vein of athymic nude mice (*n* = 5 mice per group). Mice were euthanized 25 days after the injection. The number of macroscopic lesions on the lung surfaces were quantified at necropsy from cohorts of mice injected with MDA-MB-231/sgC+EV, MDA-MB-231/sgKMT5A+EV, MDA-MB-231/sgKMT5A with SNIP1^WT^ or SNIP1^K301M^ re-expression breast cancer cells. Independent experimental groups under the same conditions were performed to calculate the animal survival (n = 5 mice per group). Data information: In (**b**–**g**), statistical analysis was performed by a two-tailed *t*-test. In (**h**), by log-rank test. ***P < 0.001. Data are represented as mean ± SEM. Panels (**a**–**h**) show one experiment representative of three independent experiments with similar results.

To investigate the roles of KMT5A-dependent SNIP1 methylation in TNBC metastatic modulation, HA-SNIP1^WT^ or HA-SNIP1^K301M^ mutant was re-expressed in MDA-MB-231/sgKMT5A and BT549 /sgKMT5A cells. Further experiments indicated that SNIP1^K301M^ overexpression, mimicking the SNIP1 K301 methylation status, but not the SNIP1^WT^, could rescue KMT5A deletion-mediated loss of MARK4 expression and transcriptional activity, YAP phosphorylation as well as its target gene expression in MDA-MB-231 and BT549 cells (Fig. 4a–c). In line with these in vitro findings, ectopic of SNIP1^K301M^, but not SNIP1^WT^, restored KMT5A depletion-mediated inhibition of proliferation and invasion (Fig. 4d, f) in vitro, as well as tumor growth and lung metastasis in vivo (Fig. 4e, g), and decreased KMT5A depletion prolonged animal survival (Fig. 4h). In addition, knockdown of KMT5A significantly restrained SNIP1 K301 methylation, the Hippo/YAP target gene CTGF expression (Supplementary Fig. 4a), cell colony formation and invasion (Supplementary Fig. 4b, d), then over-expression of MARK4 reversed the level of CTGF, cell colony formation and invasion inhibited by KMT5A knockdown in MAD-MB-231 and BT549 cells (Supplementary Fig. 4a, b, d); moreover, consistent with the results in vitro, we observed that overexpression of MARK4 could still reverse the tumor growth and lung metastasis inhibited by knockdown of KMT5A in vivo (Supplementary Fig. 4c, e), as well as shortened animal survival (Supplementary Fig. 4f). Taken together, these results demonstrate that KMT5A-dependent SNIP1 methylation promotes TNBC tumor and lung metastasis by activating MARK4 transcription and subsequent modification of the Hippo/YAP signaling pathway.

### SNIP1 K301 methylation disrupts its interaction with KAT2A and releases the inhibitory effect of SNIP1 on KAT2A HAT activity

Given the evidence that SNIP1 directly interacts with c-MYC and enhances the transcriptional activity of c-MYC^7^, we hypothesized that SNIP1 K301 methylation upregulated MARK4 by transcriptional activation of c-MYC. First, to assess whether c-MYC directly mediates MARK4 transcription, MARK4 expression was detected in *c-MYC*-knockdown breast cancer cells, where its expression was significantly downregulated in MDA-MB-231 and BT549 cells (Supplementary Fig. 5a, b). Then, the JASPAR database prediction indicated two putative c-MYC binding sites at the −1500 to −1489 bp and 97 to 108 bp of the MARK4 promoter (Supplementary Fig. 5c). Importantly, ChIP-qPCR assays also revealed that c-MYC could be recruited to the MARK4 promoter at the −1500 to −1489 bp or 97 to 108 bp site but not at the −3500 to −4000 bp control site, which was not predicted by the JASPAR database in MDA-MB-231 and BT549 cells (Supplementary Fig. 5d, e). Furthermore, the MARK4 transcription was significantly enhanced by c-MYC overexpression in HEK-293T cells, while the single mutation (MUT1) showed a decreased signal at least to some extent. Both two mutations (MUT1 + MUT2) dramatically suppressed the transcription activity (Supplementary Fig. 5f). These data suggest that c-MYC is directly bound to the MARK4 promoter, inducing MARK4 transcription.

**Fig. 5:**
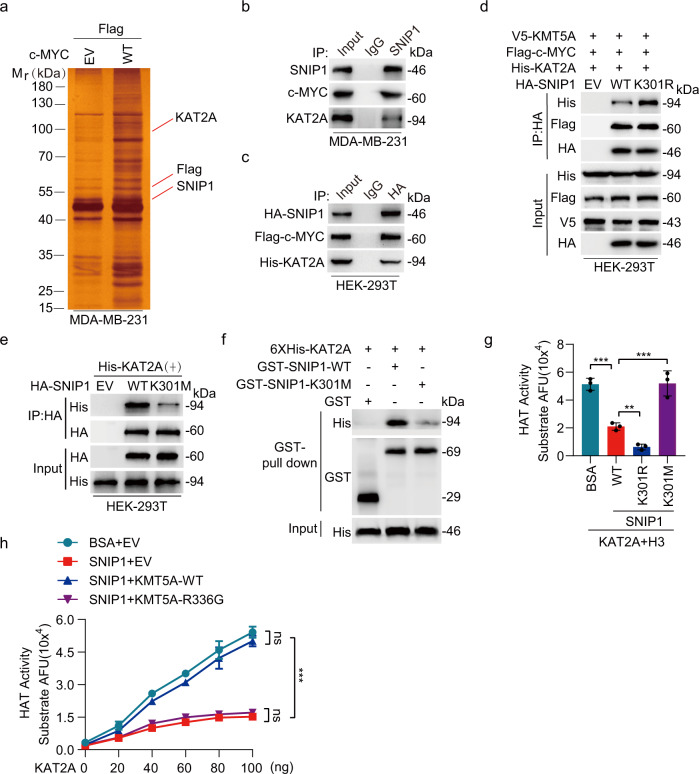
SNIP1 K301 methylation disrupts its interaction with KAT2A and releases the inhibitory effect of SNIP1 on KAT2A HAT activity. **a** Silver staining revealed c-MYC-bound proteins from MDA-MB-231 cells. **b**, **c** Immunoprecipitation and western blotting for SNIP1 association with KAT2A and c-MYC proteins in MDA-MB-231 (**b**) and HEK-293T (**c**) cells (*n* = 3). **d** Effects of SNIP1^WT^ or SNIP1^K301R^ mutant on SNIP1 association with KAT2A and c-MYC proteins. Flag-c-MYC, V5-KMT5A, and His-KAT2A were co-transfected into HEK-293T cells with HA-SNIP1 constructs or empty vector control (EV) (*n* = 3). **e** Effects of SNIP1^WT^ or SNIP1^K301M^ mutant on SNIP1 association with KAT2A. His-KAT2A were co-transfected into HEK-293T cells with HA-SNIP1 constructs or empty vector control (EV) (*n* = 3). **f** GST pulldown and western blotting for SNIP1^WT^ or SNIP1^K301M^ association with KAT2A (*n* = 3). **g** In vitro HAT activity assays of KAT2A in the presence of SNIP1^WT^ and its corresponding mutants (SNIP1^K301R^ and SNIP1^K301M^) as indicated (*n* = 3). **h** In vitro HAT activity assays of KAT2A in the presence of SNIP1 and KMT5A^WT^ or its methyltransferase activity-deficient mutant KMT5A ^R336G^ (*n* = 3). Data information: In (**g**–**h**), statistical analysis was performed by a two-tailed t-test. Error bars ± SEM. **P < 0.01, ***P < 0.001. Panels (**b**–**h**) show one experiment representative of three independent experiments with similar results.

A growing number of functions have been attributed to SNIP1, suggesting its ability to interact with multiple cellular partners. In addition, the ability of c-MYC to activate transcription relies partly on the recruitment of cofactor complexes comprising histone acetyltransferase KAT2A^29^. KAT2A is recruited to chromatin by the transcriptional activator c-MYC, and acts as a co-activator of gene transcription by acetylating histone H3, resulting in c-MYC binding to its target gene promoter and enhancing the transcription of its target genes^29,30^. Specific posttranscriptional modifications of non-histone proteins might influence their binding affinity for their partners. Tandem affinity purification combined with mass spectrometry suggested that c-MYC interacts with endogenous SNIP1 and KAT2A (Fig. 5a). Immunoprecipitation and western blotting assays are shown in Fig. 5b, c, where SNIP1 was further bound to KAT2A and c-MYC, suggesting that SNIP1/KAT2A/c-MYC form a complex in breast cancer cells. Further, we proposed that SNIP1 K301 methylation activates transcription by affecting its relationship with c-MYC or KAT2A. Interestingly, the findings demonstrated that the SNIP1^K301R^ mutant presented a more robust association with the KAT2A protein than SNIP1^WT^, but not c-MYC (Fig. 5d). However, this interaction was dramatically inhibited by SNIP1 K301 methylation (Fig. 5e). In addition, GST pull-down assays revealed that His-KAT2A were pulled down by purified GST-SNIP1 but not by GST alone, and GST-SNIP1-K301M obviously weakened this combination (Fig. 5f), indicating SNIP1 methylation disrupts a physical interaction between SNIP1 and KAT2A.

SNIP1 interacts with the catalytic domain of p300, causing the HAT activity of p300 to be strongly inhibited; while signal-induced binding of *BCAR4* to SNIP1 releases an inhibitory role of SNIP1 on HAT activity of p300^31^. Therefore, this work explored whether SNIP1 has a similar inhibitory effect on KAT2A HAT activity. Thus, the HAT activity of KAT2A in presence of SNIP1^WT^, SNIP1^K301R^, or SNIP1^K301M^ was determined. Similarly, it was strongly inhibited by recombinant SNIP1^WT^, and even much weaker by recombinant SNIP1^K301R^, but could be fully rescued by SNIP1^K301M^ (Fig. 5g). Furthermore, in vitro observations indicate that SNIP1 directly binds to KAT2A, suggesting a possible role of KMT5A-mediated SNIP1 K301 methylation in regulating the HAT activity of KAT2A. In the presence of BSA and empty vector (EV), KAT2A exhibited dose-dependent HAT activity; this was abolished in the presence of SNIP1^WT^ alone (Fig. 5h). In contrast, in the presence of KMT5A^WT^ but not inactivated kinase activity mutant KMT5A^R336G^, KAT2A HAT activity was largely rescued (Fig. 5h). These data suggest that the KMT5A-mediated SNIP1 K301 methylation releases its interaction with KAT2A, ultimately causing the activation of KAT2A enzyme activity.

### SNIP1 K301 methylation facilitates c-MYC-dependent recruitment of KAT2A to MARK4 promoter and activates the MARK4 transcriptional activity

KAT2A acts as an acetyltransferase or succinyltransferase, catalyzing acetylation of histone H3 at ‘Lys-9’ (H3K9ac) or succinylation of histone H3 on ‘Lys-79’ (H3K79succ), with a maximum frequency around the transcription start sites of genes; it also provides a specific tag for epigenetic transcription activation^32,33^. To confirm whether KAT2A regulates MARK4 expression by epigenetically activating transcription, two different lentiviruses specifically targeting KAT2A or a non-silencing control were used to deplete KAT2A in MDA-MB-231 and BT549 cells (Supplementary Fig. 6a). Interestingly, KAT2A knockdown did not affect the binding of SNIP1 and c-MYC; however, it lowered MARK4 and CTGF expression; and increased YAP phosphorylation (Supplementary Fig. 6a). Furthermore, KAT2A knockdown sharply suppressed MARK mRNA expression (Supplementary Fig. 6b), MARK4 transcription activity (Supplementary Fig. 6c), and the recruitment of KAT2A to the MARK4 promoter (Supplementary Fig. 6d, e). Also, it also decreased H3K79succ and H3K9ac levels of the MARK4 promoter after KAT2A silencing in MDA-MB-231 and BT549 cells (Supplementary Fig. 6d, e). Furthermore, SNIP1 and KAT2A co-occupied the promoter of c-MYC targeted gene MARK4 but less frequently occupied the promoter of a control gene VEGF (Supplementary Fig. 6f, g). In addition, KAT2A significantly augmented KMT5A^WT^ but not KMT5A^R336G^ induced SNIP1 methylation mediated-activation of target gene transcription (Supplementary Fig. 6h). These findings suggest that KAT2A collaborates with KMT5A-mediated SNIP1 methylation; they co-occupy the promoter of target gene MARK4 to induce transcriptional activation.

**Fig. 6:**
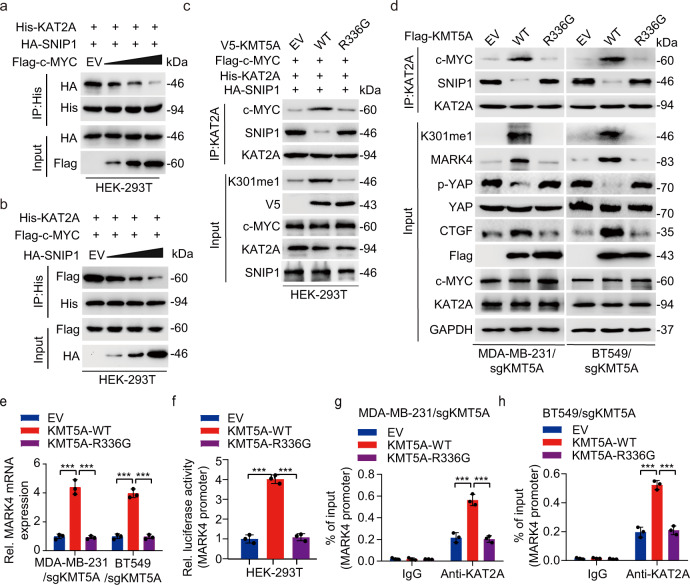
KMT5A-mediated SNIP1 K301 methylation facilitates c-MYC-dependent recruitment of KAT2A to MARK4 promoter and activates the MARK4 transcriptional activity. **a**, **b** c-MYC overexpression disrupted KAT2A-SNIP1 interaction in HEK-293T cells (**a**). SNIP1 overexpression impaired KAT2A-c-MYC association in HEK-293T cells (**b**). HEK-293T cells were transfected with the indicated plasmids. 48 h after transfection, cells were harvested for co-immunoprecipitation (*n* = 3). **c** KMT5A wild type but not the catalytically inactivated R336G mutant increased formation of the KAT2A/c-MYC complex and disrupted KAT2A-SNIP1 interaction in HEK-293T cells. HEK-293T cells were transfected with the indicated plasmids. The amount of c-MYC or SNIP1 bound to KAT2A was detected by immunoblot with the anti-c-Myc or SNIP1antibody, respectively (*n* = 3). **d** Effects of KMT5A^WT^ or KMT5A^R336G^ mutant on KAT2A association with c-MYC and SNIP1, SNIP1 K301 methylation, MARK4 protein expression and the YAP signaling pathway activation. KMT5A sgRNA resistant KMT5A^WT^ or KMT5A^R336G^ constructs or empty vector control was respectively transfected into the MDA-MB-231/sgKMT5A and BT549/sgKMT5A cell lines (*n* = 3). **e** Effects of KMT5A^WT^ or KMT5A^R336G^ mutant on MARK4 mRNA expression in MDA-MB-231/sgKMT5A and BT549/sgKMT5A cell lines (*n* = 3). **f** Effects of KMT5A^WT^ or KMT5A^R336G^ mutant on MARK4 promoter transcription activity in HEK-293T cells (*n* = 3). **g**, **h** These ChIP-qPCR results were used to measure the effects of KMT5A^WT^ or KMT5A^R336G^ mutant on the binding of KAT2A at MARK4 promoter in MDA-MB-231/sgKMT5A and BT549/sgKMT5A cell lines (*n* = 3). Data information: In (**e**–**h**), statistical analysis was performed by two-tailed *t*-test. ***P < 0.001. Data are shown as mean ± SEM. Panels (**a**–**h**) show one experiment representative of three independent experiments with similar results.

SNIP1 strongly inhibits Smad/p65-dependent transcriptional activity by competing with Smad4 and p65/RelA for binding the co-activator p300^5,9^. Further, an interaction between c-MYC, SNIP1, and the KAT2A was examined to address the mechanism by which SNIP1 methylation activates MARK4 transcription. Immunoprecipitation of KAT2A followed by western blotting revealed that forced overexpression of c-MYC tampered with the interaction between KAT2A and SNIP1 (Fig. 6a). Conversely, SNIP1 overexpression also inhibited KAT2A-c-MYC interaction (Fig. 6b). These data suggest that SNIP1 might be a candidate modifier of the MARK4 transcriptional activity by competing with c-MYC for binding KAT2A. Importantly, KMT5A^WT^ but not KMT5A^R336G^, which could methylate SNIP1 K301, promoted Flag-c-MYC binding to His-KAT2A; while it significantly impaired the relationship between HA-SNIP1 and His-KAT2A (Fig. 6c). Moreover, re-expression of KMT5A^WT^ but not KMT5A^R336G^ in MDA-MB-231/sgKMT5A and BT549/sgKMT5A significantly enhanced interaction between endogenous c-MYC and KAT2A, SNIP1 k301 methylation, MARK4 expression and the Hippo/YAP target gene CTGF expression, simultaneously, this was with a sharply decreased endogenous SNIP1 binding to KAT2A, and re-expression of KMT5A^WT^ also reduced YAP phosphorylation (Fig. 6d).

Besides, re-expression of KMT5A^WT^ but not KMT5A^R336G^, upregulated MARK4 mRNA expression in MDA-MB-231/sgKMT5A and BT549/sgKMT5A cells (Fig. 6e). Overexpression of KMT5A^WT^ but not KMT5A^R336G^ significantly enhanced the MARK4 transcription activity in HEK-293T cells (Fig. 6f). Moreover, re-expression of KMT5A^WT^ but not KMT5A^R336G^ promoted the binding of KAT2A to the MARK4 promoter in MDA-MB-231/sgKMT5A and BT549/sgKMT5A cells (Fig. 6g, h). These findings indicate that KMT5A-mediated SNIP1 K301 methylation releases KAT2A to promote the KAT2A HAT activity and c-MYC/KAT2A complex formation as well as the recruitment of c-MYC/KAT2A complex to MARK4 promoter. This ultimately inhibits the Hippo kinase cascade in a kinase-dependent manner by transcriptionally activating MARK4 expression.

### KMT5A catalytic activity combined with YAP signaling inhibition impedes triple-negative breast cancer progression

As previously reported, YAP/TAZ interacts with transcription factor TEAD to jointly activate target gene expression, playing critical roles in cancer biology including EMT, metastasis, drug resistance, and cancer stem cells^34,35^. Notably, verteporfin is a small molecule compound effectively inhibiting YAP/TAZ-TEAD interaction^36^. Considering the critical role of the Hippo/YAP signaling pathway in KMT5A/SNIP1/MARK4-driven breast cancer progression and metastasis, the effects of verteporfin treatment combined with KMT5A-depletion on breast cancer metastasis were assessed. Consistent with our findings, KMT5A knockdown significantly curbed lung metastasis of MDA-MB-231 and 4T1 cell lines in vivo (Supplementary Fig. 7a, b). In contrast with the empty vector control, re-expression of shRNA-resistant KMT5A^WT^ but not the KMT5A^R336G^ mutant re-established the KMT5A knockdown-inhibited lung metastasis in both two models (Supplementary Fig. 7a, b). These data confirm that the pro-metastatic functions of KMT5A require its catalytic activity.

**Fig. 7:**
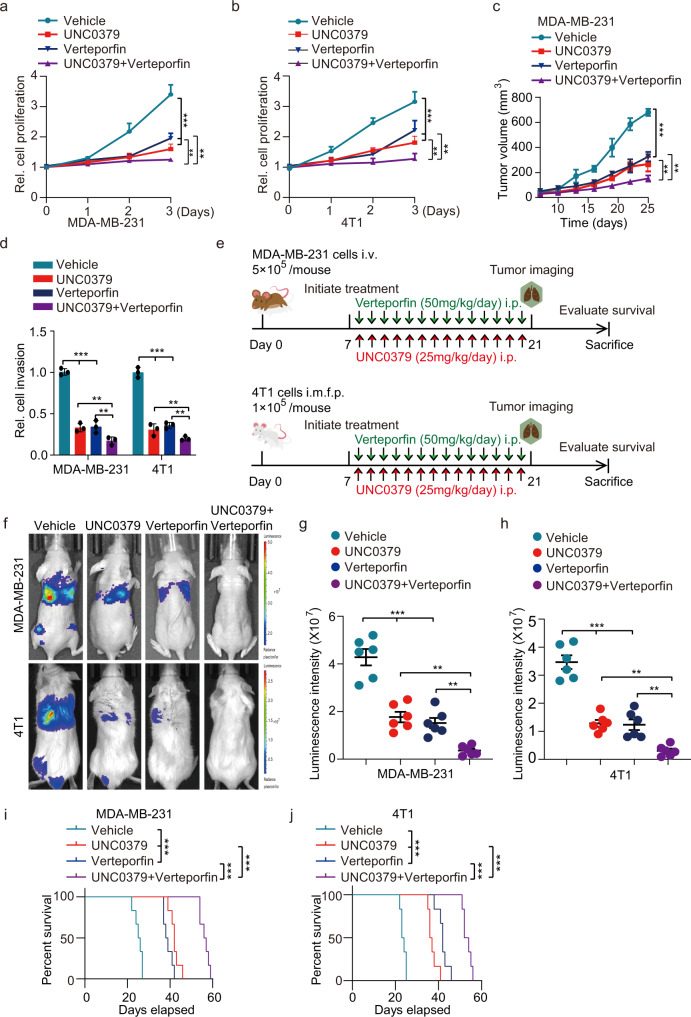
KMT5A catalytic activity depletion combined with YAP signaling inhibition impedes triple-negative breast cancer progression. **a**, **b** Effects of UNC0379 or/and verteporfin on MDA-MB-231 and 4T1 cells proliferation. Cells were treated with UNC0379 (5 μM), verteporfin (10 μM) or placebo (vehicle) as indicated (*n* = 3). **c** Cells generated in (**a**) were subjected to mouse xenograft assays by orthotopic injection in athymic nude mice, then 7 days after cell inoculation, these mice were treated with either verteporfin (50 mg/kg, i.p.) or/and UNC0379 (25 mg/kg, i.p.) daily for another 14 days, and tumor sizes were monitored and analyzed (*n* = 6 mice per group). **d** Invasion analysis of MDA-MB-231 and 4T1 cell lines treated with UNC0379 or/and verteporfin. Cells were treated with UNC0379 (5 μM), verteporfin (10 μM) or placebo (vehicle) as indicated (*n* = 3). **e** Treatment schedules for the administration of UNC0379 (25 mg/kg, intraperitoneal injection once daily) or/ and verteporfin (50 mg/kg, intraperitoneal injection once daily) to mice grafted with MDA-MB-231 cells (upper) or 4T1 cells (lower). Control mice received placebo (vehicle). Intravenous injection (i.v.). Orthotopic mammary fat pad injection (i.m.f.p.). Intraperitoneal injection (i.p.) (*n* = 6 mice per group). **f** Representative bioluminescence images in (**e**) on day 21. **g**, **h** Quantification of the bioluminescence activity of MDA-MB-231 (**g**) and 4T1 (**h**) tumor xenografts from UNC0379 or/and verteporfin treated and control mice in (**e**). **i**, **j** Kaplan–Meier survival analysis of mice with MDA-MB-231 (**i**) and 4T1 (**j**) tumor xenografts (*n* = 6 mice per group). Data information: In (**a**–**c**), statistical analysis was performed by a two-tailed *t*-test. In (**d**, **g**, **h**), by two-tailed Student’s *t*-test or one-way ANOVA. In (**i**, **j**), by log-rank test. ***P < 0.001, **P < 0.01. Data are represented as mean ± SEM. Panels (**a**–**d**) and (**f**–**j**) show one experiment representative of three independent experiments with similar results.

Furthermore, the treatment of verteporfin or KMT5A depletion showed a modest inhibitory effect on MDA-MB-231 and 4T1 cells invasiveness, respectively (Fig. 7c). Strikingly, combined treatment of verteporfin with KMT5A depletion was more effective than that of single-agent treatment in both breast cancer cell lines invasion (Supplementary Fig. 7c). To find out whether this synergy occurs in vivo, athymic nude mice injected intravenously with MDA-MB-231 cells and Balb/c mice injected orthotopically with 4T1 cells were adopted (Supplementary Fig. 7d); 7 days after inoculation, mice transplanted with MDA-MB-231 and 4T1 with or without *KMT5A* depletion were treated with either verteporfin (50 mg/kg, i.p.) or vehicle daily for another 14 days; tumor metastasis was monitored at day 21 post-implantation, respectively. Notably, tumor metastasis was robustly decelerated in both implant models (Supplementary Fig. 7e–g) with prolonged animal survival (Supplementary Fig. 7h, i) by the combination treatment.

To furthermore examine the potential for therapeutic targeting of KMT5A in clinical practice in the near future, we also treated MDA-MB-231 and 4T1 cells with a KMT5A-selective inhibitor, UNC0379, which has been evaluated to inhibit hepatocellular carcinoma and ovarian cancer progression and metastasis^37,38^. The result showed that relative to control, the treatment of UNC0379 or verteporfin presented a modest inhibitory effect on cell proliferation in vitro and tumor growth in vivo (Fig. 7a–c); then, combined treatment of UNC0379 and verteporfin was more effective than that of single-agent treatment in cell proliferation and tumor growth (Fig. 7a–c). Furthermore, we also determine whether these two small molecule inhibitors synergy occurs in cell invasion in vitro and lung metastasis in vivo. As expected, combined treatment with UNC0379 and verteporfin significantly inhibit cell invasion, compared with single-drug treatment (Fig. 7d); and the scheme of lung metastasis in vivo was consistent to the protocol of combined treatment with verteporfin or/and KMT5A depletion (Fig. 7e). Seven days after cell inoculation, transplantation tumors were treated with either verteporfin (50 mg/kg, i.p.) or/and UNC0379 (25 mg/kg, i.p.) daily for another 14 days; tumor metastasis was monitored at day 21 post-implantation, respectively. Consistently, tumor metastasis was robustly decelerated in both implant models (Fig. 7f–h) with prolonged animal survival (Fig. 7i, j) by the combination treatment. All these data suggest that the KMT5A/SNIP1/MARK4 axis might comprise a clinically targetable vulnerability of TNBC driven by the aberrant Hippo/YAP signaling pathway.

### KMT5A expression significantly correlated with activated Hippo/YAP signaling in triple-negative breast cancer

**Fig. 8:**
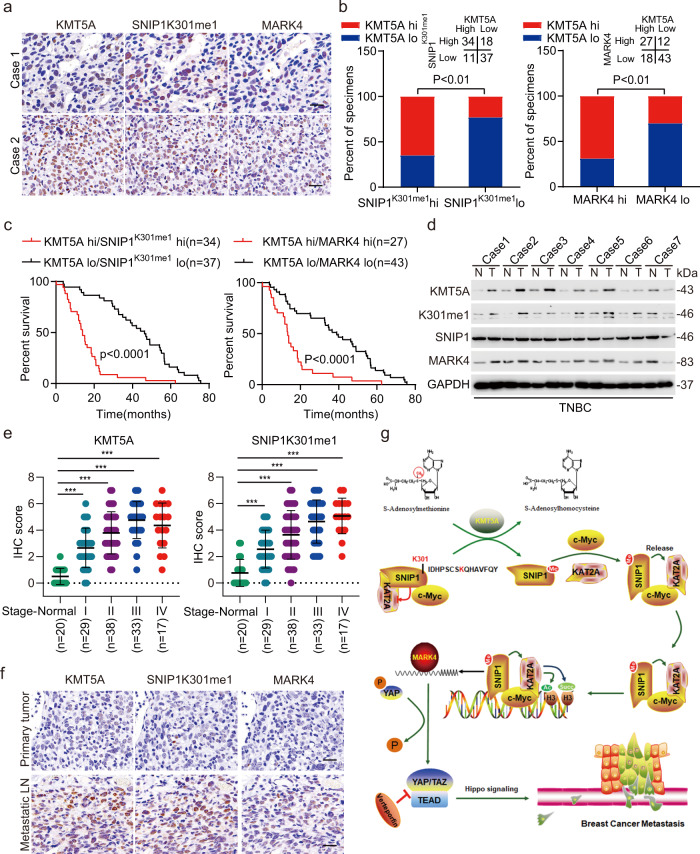
KMT5A expression was significantly correlated with activated MARK4/YAP signaling in triple-negative breast cancer. **a** Representative images of KMT5A, SNIP1K301me1, and MARK4 in 100 clinical TNBC metastatic lymph node tissues. Scale bars, 50 μm. **b** Correlation of expression levels between KMT5A, SNIP1K301me1, and MARK4 in (**a**). **c** Prognosis comparison of breast cancer patients with KMT5A/SNIP1K301me1 or KMT5A/MARK4 ectopic differential expression using Kaplan–Meier survival analysis. **d** Representative immunoblots showing KMT5A, SNIP1K301me1, and MARK4 expression in normal breast specimen (N) and paired tumor tissues (T) (*n* = 3). **e** Correlation between TNBC stages and IHC scores of KMT5A and SNIP1K301me1. **f** Representative IHC staining of KMT5A, SNIP1K301me1, and MARK4 in primary TNBC and matched metastatic lymph nodes (*n* = 100 paired samples). Scale bars: 50 μm. **g** A working model of KMT5A-promoted Hippo/YAP signaling pathway modification and triple-negative breast cancer metastasis through the SNIP1 methylation status. KMT5A methylated SNIP1 at K301, which released KAT2A for activated histone acetyltransferase (HAT) and recruited by c-MYC, leading to enhanced KAT2A anchoring onto the MARK4 promoter and MARK4 transcription, ultimately activating the Hippo/YAP signaling pathway. Data information: In (**b**), Statistical analysis was performed by chi-square test. In (**c**), by log-rank test. In (**e**), by two-tailed *t*-test. *P < 0.05, ***P < 0.001. Data are represented as mean ± SEM. Panel (**d**) shows one experiment representative of three independent experiments with similar results.

To further demonstrate the clinical relevance of our findings, the expression levels of KMT5A, SNIP1K301me1, and MARK4 were detected in 100 clinical TNBC species. As shown in Fig. 8a, KMT5A, SNIP1K301me1, and MARK4 were significantly co-expressed in TNBC tissues. According to the quantification of protein expression via immunohistochemical staining analysis, KMT5A significantly and positively correlated with SNIP1K301me1 and MARK4, as demonstrated using the chi-square test (Fig. 8b). Then the roles of KMT5A, SNIP1K301me1, and MARK4 in TNBC progression, we analyzed the correction between differences in the expression levels of KMT5A, SNIP1K301me1, and MARK4 and various clinicopathological parameters of individuals with TNBC (Table 1). All these protein levels did not show a significant correlation with age, tumor size, and T staging. However, the expression of KMT5A and MARK4 were significantly correlated with Pathological grading; additionally, SNIP1K301me1 and MARK4 levels were correlated with N staging, and SNIP1 K301me1 has a certain correlation with AJCC staging (Table 1), indicating that KMT5A-mediated methylation of SNIP1 may enhance TNBC malignant metastasis. Kaplan–Meier analysis showed that co-expression of KMT5A/SNIP1^K301me1^ or KMT5A/MARK4 at high expression levels positively correlated with a poor prognosis in patients with TNBC (Fig. 8c). Moreover, we detected KMT5A, SNIP1K301me1, and MARK4 expression in normal breast specimens and tumor tissues, and these protein levels were significantly upregulated in tumor tissues (Fig. 8d); simultaneously, we also detected KMT5A and SNIP1K301me1 expression in the tissues of different tumor stage, and found that the expression of KMT5A and SNIP1K301me1 were closely related to the progress of TNBC (Fig. 8e). Eventually, KMT5A, SNIP1K301me1, and MARK4 expression were dramatically upregulated in metastatic LN compared with primary TNBC tumors (Fig. 8f). In conclusion, all these data confirm that KMT5A-mediated methylation of SNIP1 significantly correlates with activated MARK4 kinase cascade signaling in TNBC.

**Table 1:** Correlation between different expression levels of SET8, SNIP1 K301me1, and MARK4 with clinicopathological parameters of individuals with TNBC.

		SET8			SNIP1 K301me1			MARK4		
Parameters	*N*	Low (*n* = 55)	High (*n* = 45)	*p*	Low (*n* = 48)	High (*n* = 52)	*p*	Low (*n* = 61)	High (*n* = 39)	*p*
Age (years)^a^										
≥56	50	26	24	0.5465	25	25	0.6889	31	19	0.8376
<56	50	29	21		23	27		30	20	
Tumor sizes (cm)										
≤2	17	10	7	0.8766	9	8	0.8678	13	4	0.2764
>2 + ≤5	67	37	30		32	35		40	27	
>5	16	8	8		7	9		8	8	
T staging										
T1 + T2	81	48	33	0.0771	42	39	0.1114	52	29	0.1759
T3 + T4	19	7	12		6	13		9	10	
N staging										
N1	29	18	11	0.3638	19	10	0.025	25	4	0.001
N2 + N3	71	37	34		29	42		36	35	
AJCC staging^b^										
II	26	16	10	0.436	17	9	0.0392	19	7	0.1422
III + IV	74	39	35		31	43		42	32	
Pathological grading										
I	10	9	1	0.0095	7	3	0.225	6	4	0.0032
II	78	43	35		37	41		53	25	
III	12	3	9		4	8		2	10	

^a^The age of 100 individuals with TNBC ranged from 24 to 79 years old, with a median of 56 years old.

^b^The clinical staging of the individuals was based on the AJCC staging (8th edition).

## Discussion

Effective treatment options for breast cancer, specifically for TNBC are limited. PTMs, like methylation modifications-based mechanisms, might be the crucial nodal points for therapeutic intervention in TNBC^39,40^. To identify non-histone methylated proteins implicated in oncogenic signaling pathways, an MS-based high throughput screening was performed. Consequently, we detected numerous proteins modified by lysine methylation. The present study shows that Smad nuclear-interaction protein 1 (SNIP1), could be methylated in its FHA domain by methyltransferase KMT5A. Besides, the absence of SNIP1 K301 methylation significantly repressed cell growth, invasion, and lung metastasis. More strikingly, KMT5A-mediated K301 methylation of SNIP1 represents a sensing signal to release KAT2A for activated HAT activity, while driving an interaction of c-MYC and KAT2A. Further, it promotes the recruitment of the c-MYC/KAT2A complex to the promoter of the c-MYC target; ultimately inhibiting the Hippo kinase cascade in a kinase-dependent manner by transcriptionally activating MARK4 (Fig. 8g). In addition, loss of KMT5A catalytic activity by KMT5A depletion or KMT5A-selective inhibitor combined with YAP signaling inhibition impedes breast cancer progression using a mouse xenograft model of breast cancer metastasis. Furthermore, a concomitant upregulation in KMT5A, SNIP1K301me1, and MARK4 expression depict poor prognostic factors for metastatic TNBC patients.

Methylation has evolved as a common posttranslational modification, regulating various non-histone proteins^14–17,41^. SNIP1 has been identified as a transcription repressor for the TGF-β and NF-кB signaling pathways; it disrupts the recruitment of co-activator p300^9^. It is essential for Cyclin D1 mRNA stability and expression^42^; augments c-Myc function^7^, which also cooperates with both c-Myc and H-Ras to induce foci formation in an in vitro transformation assay^7^, implicating the role of SNIP1 in tumorigenesis. SNIP1-TET2 interaction enhances the ability of TET2 to trans-activate c-MYC target genes, ultimately promoting anchorage-independent cell growth and cisplatin resistance of MCF-7 breast cancer cells^6^. Previous research demonstrated that SUMOylation of SNIP1 attenuates its inhibitory effect in TGF-β signaling, causing the loss of its-mediated inhibition on the expression of TGF-β target genes PAI-1 and MMP2; it eventually enhances TGF-β-regulated cell migration and invasion^43^. Herein, we found that SNIP1 K301 was innovatively mono-methylated. Further, the absence of SNIP1 K301 methylation significantly decreased growth, invasion, and lung metastasis of breast cancer cells, indicating that SNIP1 methylation regulates TNBC metastasis.

Once the methylation of SNIP1 at K301 is validated, it is vital to identify the methyltransferase involved. This paper performed mass spectrometry (MS) analysis to screen known histone methyltransferases (HMTs) and determine whether they might function as SNIP1 methyltransferases. Consequently, KMT5A was identified as a SNIP1-interacting protein methylating non-histone SNIP1 at K301. KMT5A is the only known monomethyltransferase of histone 4 at lysine 20 (H4K20me1). In addition to histone modifications^44^, KMT5A mono-methylates non-histone protein p53 and PCNA^21,45^. Furthermore, KMT5A involvement in cell proliferation and genome stability as well as the importance of its non-histone target p53 in cell-cycle control predict its possible role in tumorigenesis^21^. KMT5A facilitates hepatocellular carcinoma growth by enhancing aerobic glycolysis^46^. Notably, it also acts as a dual epigenetic modifier on the promoters of TWIST target genes E-cadherin and N-cadherin via its H4K20 mono-methylation activity, demonstrating its metastatic potential in breast carcinoma^47^. We also demonstrated that KMT5A methylates SNIP1 K301 to activate vital oncogenic pathway-Hippo/YAP signaling, as well as its overexpression in TNBC and relationship with TNBC prognosis. Moreover, loss of KMT5A catalytic activity by KMT5A depletion or KMT5A-selective inhibitor, and combined with YAP signaling inhibition significantly impairs breast cancer progression and metastasis. In addition, elevated KMT5A and methylated SNIP1 are associated with clinical TNBC metastasis and prognosis.

This study, therefore, confirms that SET-mediated SNIP1 K301 methylation regulates c-MYC transcriptional activity to transcriptionally activate MARK4; this was associated with different co-regulators. Moreover, c-MYC functions with multiple histone acetyltransferases, including KAT2A, TIP60, and CBP^29,48,49^. c-MYC transformation domain recruits human STAGA (SPT3-TAF-KAT2A acetylase) coactivator complex and particularly requires KAT2A HAT activity, as a co-activator for c-MYC in regulating c-MYC target genes^29^. Our current research revealed that KMT5A-mediated SNIP1 K301 methylation released KAT2A, promoting c-MYC/KAT2A complex formation, and transcriptionally activating the target gene MARK4 of c-MYC; this was consistent with previous studies that a model for c-MYC-dependent transcription activation on chromatin involved direct recruitment by c-MYC of KAT2A^50^. c-MYC is also a substrate of KAT2A acetyltransferase activity; its acetylation of K323 triggers a dramatic increase in c-MYC protein stability^51^. Therefore, these data suggest that a stable existence of the c-MYC/KAT2A complex promotes a continuous transcriptional activation of c-MYC target genes.

KAT2A was identified as a histone acetyltransferase, enhancing H3K9 acetylation^33^. Lately, KAT2A coupled with the -KGDH complex regulates H3K79 succinylation^52^, which plays a crucial role in transcription activation by providing a scaffold for the formation of complex transcriptional components^32^. KAT2A HAT activity for histone H3 modifications has been confirmed to be associated with the poor prognosis of many cancers. For instance, KAT2A-mediated H3K79 succinylation in the promoter of *YWHAZ* (encoding 14-3-3zeta expression) is essential for β-catenin stability to promote pancreatic carcinoma metastasis^53^. KAT2A recruited a nuclear receptor binding protein TIF1β by mediating H3K9 acetylation to activate NF90-maintained HIF1α stability, further enhancing nasopharyngeal carcinoma progression^33^. We revealed that SNIP1 K301 methylation released its interaction with KAT2A, and reversed the inhibition of KAT2A HAT activity by SNIP1; this was similar to the previous research, where SUMOylation of SNIP1 attenuated its inhibitory effect in TGF-β signaling as SUMO-conjugated form of SNIP1 with impaired ability to disrupt the interaction between p300 and Smads^43^. Furthermore, KAT2A increased H3K79succ and H3K9ac levels of the MARK4 promoter and co-occupied the promoter of MARK4 with SNIP1 to enhance its transcriptional activation. In addition, the EZH2/H3K27me3/DNMT1 complex promotes the YAP pathway in breast cancer^54^. As the major functional transducer of the Hippo pathway, YAP has been identified as an oncogene, which was regulated by diverse upstream signals like MARK4^26^ and has been linked to many cancers such as liver cancer, breast cancer, and pancreatic cancer^55,56^. And our results identified UNC0379, a KMT5A selective inhibitor, cooperates with YAP signaling inhibition to prevent breast cancer progression and metastasis, which may help overcome the narrow therapeutic index of single treatment strategy.

In summary, we identify KMT5A-mediated SNIP1 K301 methylation as a crucial initiation event for subsequent c-MYC-activating the key oncogenic pathway-Hippo signaling, which depends on SNIP1 K301 methylation releasing KAT2A for activated HAT activity and promoting the formation of c-MYC/KAT2A complex, eventually transcriptional activating MARK4. Importantly, this study reveals that KMT5A-mediated SNIP1 K301 methylation not only serves as a key signal driving c-MYC hyper-activation and cancer metastasis, but also acts as a marker of poor prognosis of TNBC patients. These findings add to our understanding on the role of SNIP1 in cancer metastasis. Furthermore, these results provide ideas for developing therapeutic strategies for preventing cancer metastasis.

## Methods

### Cell lines and cell culture

MCF10A, T47D, MDA-MB-453, BT474, MCF7, BT549, MDA-MB-231, and HEK-293T cell lines were purchased from the Chinese National Infrastructure of Cell Line Resource (Beijing, China). The 4T1 cell line was obtained from the American Type Culture Collection (ATCC). All human cell lines were authenticated before the start of experiments using STR DNA fingerprinting at Shanghai Biowing Applied Biotechnology Co., Ltd. (Shanghai, China). T47D, MDA-MB-453, BT474, MCF7, BT549, MDA-MB-231, and HEK-293T cells were cultured in DMEM (Dulbecco’s modified Eagle’s medium) supplemented with 10% FBS (Gibco™,10100147) and 100 U/ml penicillin-streptomycin. MCF10A cells were cultured in DMEM/F12 (Invitrogen) supplemented with 20 ng/ml epidermal growth factor, 5% horse serum, 0.5 μg/ml hydrocortisone, 10 μg/ml insulin, 100 ng/ml cholera toxin, and 100 μg/ml penicillin-streptomycin. 4T1 cells were maintained in RPMI-1640 medium supplemented with 10% FBS and 100 U/ml penicillin-streptomycin. All cell lines were cultured at 37 °C and 5% CO_2_. Mycoplasma contamination was detected with a LookOut Mycoplasma PCR Detection Kit (Sigma-Aldrich).

### Patient specimens

Human tissue samples of TNBC were provided by Zhejiang Provincial People’s Hospital (Hangzhou, China). The study protocol was approved by the Research Ethics Board of the Zhejiang Provincial People’s Hospital. Written informed consent was obtained from all participants in the study. All the research was carried out according to the provisions of the Declaration of Helsinki of 1975. None of these patients had received any chemotherapy or radiotherapy before surgery. The study is compliant with the “Guidance of the Ministry of Science and Technology (MOST) for the Review and Approval of Human Genetic Resources” in China.

### Chromatin immunoprecipitation (ChIP), re-ChIP, and quantitative RT-PCR

ChIP was performed by a SimpleChIP® Plus Enzymatic Chromatin IP Kit (Magnetic Beads, Cell Signaling Technology, 9005 S). Briefly, cells were crosslinked with 1% formaldehyde in PBS at RT for 10 min, quenched with 2.5 M glycine at RT for 5 min and washed with PBS three times. Nuclei were prepared, and chromatin was incubated with micrococcal nuclease at 37 °C for 20 min, which was followed by an appropriate amount of sonication. The supernatants were immunoprecipitated by being incubated with 3 µg of anti-c-MYC, anti-KAT2A, anti-SNIP1 or nonspecific rabbit IgG at 4 °C for 12–16 h. The next day, the immunocomplexes were rotationally incubated with 30 µl of ChIP-Grade Protein G Magnetic Beads for 2 h at 4 °C and then were washed three times using low salt wash buffer and 1 time with high salt wash buffer at 4 °C for 5 min per wash. Chromatin was eluted by ChIP elution buffer for 30 min at 65 °C with gentle vortex mixing (1200 rpm) and crosslinks were reversed by treatment with 5 M NaCl and proteinase K overnight at 65 °C. Samples were then incubated with RNase at 37 °C for 1 h. CHIP DNA was purified and subsequently quantified by quantitative real-time PCR (qPCR). Gene expression levels were normalized to the expression level of beta-Actin. Data analysis was finally presented as percentages of the input DNA. Re-ChIP assays were performed using the Re-ChIP-IT kit (Active Motif). Briefly, the precipitated chromatin from the first ChIP reaction was eluted by 100 µl diluted Re-ChIP-IT elution buffer at RT for 30 min. Next, the eluted precipitate was desalted by the desalting column provided in the kit. The second ChIP was performed with 30 µl Protein G magnetic beads, 90 µl desalted chromatin, and 3 µg second antibody. Next, the second precipitate was washed, eluted, and reverse cross-linked as in the first ChIP. DNA was obtained by phenol and phenol/chloroform extractions, and subjected to real-time PCR evaluation. Primer sequences for PCR are available upon request.

### Western blotting and immunoprecipitation (IP) assays

Total cells were lysed by incubation at 4 °C for 30 min in IP lysis buffer (20 mM Tris-HCl, pH 7.5, 150 mM NaCl, 1 mM EDTA, 2 mM Na_3_VO_4_, 5 mM NaF, and 1% Triton X-100) supplemented with complete protease inhibitor cocktail (Roche). The lysates were cleared by centrifugation, separated by SDS-PAGE and analyzed by immunoblotting. To detect SNIP1 methylation in vivo, cells were lysed in IP buffer supplemented with a complete protease inhibitor cocktail, which was followed by sonication and centrifugation at 12,000 × *g* for 15 min at 4 °C. The supernatants were immunoprecipitated by incubation with a SNIP1 antibody or an HA antibody for 12 h at 4 °C. The immunoprecipitants were washed six times with IP buffer and then were boiled in 1X SDS-loading buffer for immunoblot analysis.

To detect protein interactions, cells were lysed in IP buffer, and the supernatants were immunoprecipitated with the indicated antibodies, slowly shaken on a rotating shaker at 4 °C overnight and then incubated with Pierce™ Protein A/G Magnetic Beads (Invitrogen) at room temperature for 1 h with mixing. The immunoprecipitants were washed five times using wash buffer (25 mM Tris, 0.5 M NaCl, 0.05% Tween-20, pH 7.5), then were eluted, and boiled in 1X SDS loading buffer to prepare the samples for immunoblot analysis. For SNIP1-associated protein detection, ~4 × 10^8^ MDA-MB-231 cells overexpressing Flag-SNIP1 or Flag-GFP control were harvested in IP lysis buffer supplemented with a complete protease inhibitor cocktail for assays. Equal amounts of cell lysates, including FLAG-tagged SNIP1, c-MYC or Flag-GFP control protein, were immunoprecipitated with anti-FLAG (Sigma-Aldrich) and Pierce™ Protein A/G Magnetic Beads (Invitrogen) at 4 °C for the appropriate time. Beads were washed with wash buffer and the binding protein was eluted with elution buffer (0.1 M glycine, pH 2.0) at RT with mixing for 10 min. After magnetically separating the beads, the saved supernatants containing the target antigen were neutralized immediately by neutralization buffer (1 M Tris HCl, pH 8.5), then were boiled, resolved by SDS-PAGE and silver-stained. Bands were excised and subjected to LC-MS/MS sequencing and data analysis.

### Purification of recombinant proteins

HEK-293T cells was transfected with 6XHis-tagged SNIP1 lysed at 4 °C for 30 min in buffer containing 10 mM HEPES (pH 7.6), 3 mM MgCl_2_, 300 mM KCl, 5% glycerol, 0.5% NP-40, 10 mM imidazole, 1 mM Na_2_VO_4_, 20 mM NaF, 1 mM sodium pyrophosphate, 25 mM β-glycerophosphate, and 1X complete EDTA-free complete protease inhibitor cocktail (Roche). Then, the lysates were sonicated and centrifuged at 4 °C and 21,000 × *g* for 20 min. The supernatants were then loaded onto a pre-equilibrated HisPur Cobalt resin (Thermo Scientific) column. Then, the loaded resin was washed with equilibration/wash buffer (50 mM sodium phosphate, 300 mM sodium chloride, and 10 mM imidazole, pH 7.6). After washing two times, the His-tagged SNIP1 proteins were eluted with elution buffer (50 mM sodium phosphate, 300 mM sodium chloride, and 250 mM imidazole, pH 7.6) from the resin, and several fractions were collected and subjected to dialysis against PBS. The purified recombinant proteins were further examined.

### GST and His pulldown assay

Recombinant GST-conjugated KMT5A and histidine (His)-conjugated SNIP1 were generated by transforming *Escherichia coli* BL21 with pGEX-4T-1-KMT5A and pET-28a (+)-SNIP1, respectively. Five milliliters of bacterial cultures that were grown overnight at 37 °C in a 150 rpm shaking incubator were inoculated into 500 ml ampicillin- or kanamycin-resistant Luria-Bertani broth (LB). Cell cultures were grown at 37 °C until the log phase was reached, as indicated by an optical density of 0.8; then protein expression was induced by the addition of 0.25 mM IPTG, and cells were placed at 16 °C for 16 h with continuous shaking. After induction, the cultures were centrifuged at 4 °C 5000 rpm for 10 min, supernatants were discarded, and pellets were resuspended in 30 ml lysis buffer (1X PBS, 500 mM NaCl, 1% Triton X-100, 0.5 mg/ml lysozyme, and 1X EDTA-free protease inhibitor cocktail) and sonicated on ice. The lysate was centrifuged at 15000 rpm for 15 min at 4 °C. Supernatants were incubated with the corresponding volume of 50% glutathione Sepharose 4B bead slurry (GE Healthcare) or Ni-NTA Magnetic Agarose Beads (Qiagen) at 4 °C for 8 h with end-over-end mixing following the manufacturer’s instructions. The glutathione beads were washed three times using lysis buffer and then were stored at 4 °C in lysis buffer, samples were eluted with elution buffer (10 mM glutathione in 50 mM Tris-HCl pH 8.0). The Ni beads were washed three times with 50 mM Tris-Cl (pH 8.0) wash buffer containing 20 mM imidazole (1 ml per wash), and samples were eluted with Tris elution buffers containing 250 mM imidazole.

### In vitro methylation assay

GST-KMT5A and 6xHis-SNIP1 proteins were purified utilizing glutathione beads and Ni-NTA agarose beads, respectively, as described above, according to the manufacturer’s procedures. In brief, 1 µg of GST-tagged methyltransferase was incubated with 2.5 µg of His-tagged substrate in 50 µl of reaction buffer [50 mM Tris-HCl (pH 8.0), 10% glycerol, 20 mM KCl, 5 mM MgCl_2_, 1 mM DTT, and 1 mM PMSF] supplemented with 2 µg Ci S-adenosyl-L-[methyl-3H] methionine (Amersham Biosciences) for 2 h at 30 °C. Reactions were resolved by SDS-PAGE for Coomassie staining (Expedeon, InstantBlue) and then were exposed to an autoradiogram for the final analysis.

### In-gel digestion

For in-gel tryptic digestion, gel pieces were destained in 50 mM NH_4_HCO_3_ in 50% acetonitrile (v/v) until they were clear. Gel pieces were dehydrated with 100 μl of 100% acetonitrile for 5 min, the liquid was removed, and the gel pieces were rehydrated in 10 mM dithiothreitol and incubated at 56 °C for 60 min. Gel pieces were again dehydrated in 100% acetonitrile, the liquid was removed, and gel pieces were rehydrated with 55 mM iodoacetamide. Samples were incubated at room temperature in the dark for 45 min. Gel pieces were washed with 50 mM NH_4_HCO_3_ and then dehydrated with 100% acetonitrile. Gel pieces were rehydrated with 10 ng/μL trypsin resuspended in 50 mM NH_4_HCO_3_ on ice for 1 h. Excess liquid was removed, and gel pieces were digested with trypsin at 37 °C overnight. Peptides were extracted with 50% acetonitrile/5% formic acid, which was followed by 100% acetonitrile. Peptides were dried to completion and resuspended in 2% acetonitrile/0.1% formic acid.

### LC-MS/MS analysis

Tryptic peptides were dissolved in 0.1% formic acid (solvent A) and directly loaded onto a custom-made reversed-phase analytical column (15-cm length, 75 μm i.d.). The gradient comprised an increase from 6 to 23% solvent B (0.1% formic acid in 98% acetonitrile) over 16 min, 23 to 35% in 8 min before climbing to 80% in 3 min and then holding at 80% for the last 3 min; this all occurred at a constant flow rate of 400 nl/min on an EASY-nLC 1000 UPLC system. The peptides were subjected to NSI source followed by tandem mass spectrometry (MS/MS) in Q Exactive HF (Thermo) coupled online to the UPLC. The electrospray voltage applied was 2.0 kV. The *m/z* scan range was 350–1800 for a full scan, and intact peptides were detected in the Orbitrap at a resolution of 70,000. Peptides were then selected for MS/MS with the NCE set at 28, and the fragments were detected in the Orbitrap at a resolution of 17,500. A data-dependent procedure that alternated between one MS scan followed by 20 MS/MS scans with 15.0 s dynamic exclusion. The automatic gain control (AGC) was set at 5E4.

### Data processing

The resulting MS/MS data were processed using MASCOT. The retrieval parameters were set as follows: the database was set to UNIPROT human (20214 sequences). Trypsin/P was specified as a cleavage enzyme allowing up to two missing cleavages. The mass error was set to ±10 ppm for precursor ions and 0.02 Da for fragmentations. Carbamidomethyl (C) and methyl (K/R) were specified as fixed modifications, and acetyl (protein N-term) and oxidation (M) were specified as variable modifications. Peptide confidence was set at high, and the peptide ion score was set at >20.

### In vitro KAT2A HAT assay

The in vitro KAT2A HAT assay was performed using the HAT assay kit from the Active Motif according to the manufacturer’s instruction.

### Luciferase reporter assays

pGL3.0 basic with the wild-type MARK4 promoter and the promoter with mutated c-Myc binding sites were cotransfected with or without c-Myc using Lipofectamine™ 3000 Transfection Reagent (Thermo Fisher) according to the manufacturer’s protocol. A pRL Renilla luciferase control reporter vector (Promega) was utilized as a negative control. A dual-luciferase assay was performed 48 h after cotransfection using the Promega E1960 Dual-Luciferase® Reporter System following the manufacturer’s recommendations.

### Antibodies

Antibodies for KMT5A (1:1000 for WB, 1:200 for IHC and Co-IP, #2996), VEGF (1:50 for ChIP, #50661), GAPDH (1:1000 for WB, #5174), MARK4(1:1000 for WB, 1:200 for IHC, #4834), MST2(1:1000 for WB, #3952), p-MST2(1:1000 for WB, Thr180, #49332), YAP (1:1000 for WB, #14074), p-YAP (1:1000 for WB, Ser127, #13008), SAV1 (1:1000 for WB, #13301), LATS1 (1:1000 for WB, #3477), Phospho-Ser/Thr (1:1000 for WB, #9631), CTGF (1:1000 for WB, #10095), Mono-Methyl Lysine (1:1000 for WB, #14679), HA (1:1000 for WB, 1: 200 for Co-IP, #3724), His (1:1000 for WB, 1:200 for Co-IP, #12698), KAT2A (1:1000 for WB, 1:200 for Co-IP, 4 μg for ChIP, #3305), c-Myc (1:1000 for WB, 4 μg for ChIP, #18583), and mono-methyl lysine (1:1000 for WB, #14679) were purchased from CST (Danvers, MA); SNIP1 K301 mono-methylation rabbit polyclonal antibody generated by Proteintech (US) using the peptide IDHPSCS-K(me)-QHAVFQY (1:1000 for WB, 1: 200 for IHC); an antibody for FLAG (1:1000 for WB, 1:50 for Co-IP, F3165) was from Sigma-Aldrich (US); antibodies for GAPDH (sc-25778) and GST (1:1000 for WB, sc-138) were from Santa Cruz Biotechnology (CA); antibodies for Phospho-Ser/Thr (1:1000 for WB, ab17464), SNIP1 (1:1000 for WB, 1:100 for Co-IP, 4 μg for ChIP, ab126194), acetyl-Histone H3 (Lys9) (4 μg for ChIP, ab4441), and V5-tag (1:1000 for WB, 1:200 for Co-IP, ab9113) were purchased from Abcam (UK); an antibody for succinyl-Histone H3 (Lys79) (4 μg for ChIP, PTM-412) was purchased from PTM Biolabs (US); the secondary antibodies, anti-rabbit IgG, HRP-linked antibody (1:5000, #7074) and anti-mouse IgG, HRP-linked antibody (1:5000, #7076) were purchased from CST (Danvers, MA).

### Plasmids

KMT5A, SNIP1, c-Myc, and KAT2A DNAs (cDNAs) were purchased from GeneChem (Shanghai, China). They were then sequenced and subcloned into the pcDNA3.3 or pLVX-Puro vector (Clontech). KMT5A or SNIP1-truncated constructs were generated by PCR using KMT5A or SNIP1-pcDNA3.3 as templates, and then they were inserted into pcDNA3.3. GST-SNIP1 was constructed by cloning the wild-type cDNA into the pGEX-4T-1 vector. SNIP1 mutants (K301R, K325R, and K342R) and KMT5A (G336G) were purchased from Bioegene (Shanghai, China). The MARK4 promoter, extending from −2000 to +200 relative to the transcription start site, was cloned into the pGL3-basic luciferase reporter vector and was used as a template to subclone and generate a series of promoter deletions of MARK4. The promoter of MARK4 with disrupted potential c-Myc binding sites was constructed using PCR-based site-directed mutagenesis. The primers are listed in Supplementary Table 1.

### shRNA-knockdown, sgRNA-knockout, and transfection assays

shRNA-knockdown, single-guide RNA (sgRNA)-knockout, and transfection assays were performed as previously described^57^. KMT5A or SNIP1 sgRNA sequences were designed using the MIT online tool (http://crispr.mit.edu). shRNA sequences were purchased from GeneChem (Shanghai, China). DNA and packaging plasmids were transfected into HEK293 cells. The supernatants were filtered through a 0.22 µm membrane (Millipore) at 48 and 72 h after transfection and then concentrated. MDA-MB-231 and BT-549 cells were infected using lentivirus expressing shRNAs or shGFP control with 8 µg/ml polybrene. Infected cells were selected by treatment with 5 μg/ml puromycin 48–72 h after infection. Multiple monoclonal cultures were screened for shRNAs by western blotting and RT-PCR analysis. For transient transfection, plasmids were transfected using Lipofectamine 3000 (Invitrogen) reagent following the manufacturer’s instructions.

### Transwell invasion assays

A total of 5 × 10^4^ cells suspended in a medium without FBS were plated on the upper chamber membranes (8 µm pore size, 5 mm diameter, Corning), which were coated with Matrigel (BD Biosciences). The insert was incubated in 500 µl of medium supplemented with 10% FBS (Invitrogen) for 16–24 h. To evaluate the cell invasion capacity, noninvasive cells were removed by softly swiping the top of the membrane using cotton swabs, and migrated or invasive cells were fixed with methanol, stained with crystal violet, and counted under a light microscope.

### Histology and immunohistochemical staining

Mice lungs were collected by necropsy, and fixed in 4% paraformaldehyde, embedded in paraffin. Four-step sections (100 mm per step) were obtained from each set of lungs. The sections were stained with H&E and scanned using a Scanscope XT digital slide scanner (Aperio Technologies). Digital images of lung sections were used to analyze the metastatic burden. Immunohistochemistry (IHC) for patients’ tissues was performed using anti-KMT5A (1:500), anti-SNIP1K301me1 (1:500), and anti-MARK4 (1:100) antibodies. Each sample was assigned a score according to the intensity of the staining (0 = no staining, 1 = weak staining, 2 = moderate staining, and 3 = strong staining) and the proportion of the stained cells (0 = 0%, 1 = 1–25%, 2 = 25–50%, 3 = 50–75%, and 4 = 75–100%). Negative control slides without primary antibodies were included. Core staining was scored as negative (0) when <10% of tumor cells exhibited positive expression. A score ranging from 0 to 7 was calculated by adding the staining extent score with the intensity score, resulting in a low (0–3) level or a high (4–7) level value for each specimen. The stained tissues were scored by three individuals blinded to the clinical parameters.

### RNA-Seq and differentially expressed gene analysis

RNA-Seq and differentially expressed gene analysis were performed as previously described^32^. In brief, total RNA was extracted and purified with a Qiagen RNeasy Mini Kit (Valencia, CA, USA) according to the manufacturer’s protocols. The RNA quality was evaluated by an Agilent 2100 Bioanalyzer before sequencing. Libraries for poly(A) + RNA were prepared according to the Illumina protocol. Libraries were sequenced on Illumina HiSeqX Ten platforms. Genes that were found to be statistically significantly differentially expressed between the two groups were identified through fold change filtering (fold change ≥ 2.0; *P* ≤ 0.05). The differentially expressed genes were used in a KEGG pathway analysis using DAVID software (https:// david.ncifcrf.gov/). Fisher’s exact or chi-square test with FDR was used to calculate the *P*-values of KEGG pathways, respectively. A threshold of *P* < 0.05 and FDR < 0.25 was used to select significant items.

### In vivo xenograft experiments

4–5 weeks-old pathogen-free female BALB/c and athymic nude mice were purchased from the Slaccas (Shanghai) and maintained in a mouse-specific pathogen-free (SPF) facility for 1 week before injection to allow the mice to adjust to their new environment. And 5-8 mice per cohort were used for each independent experiment. MDA-MB-231 cells (1 × 10^5^) or their derivatives were suspended in 100 μl of PBS and then were injected into the tail vein of nude mice, while MDA-MB-231 cells (5 × 10^5^) or 4T1 cells (5 × 10^5^) or their derivatives were suspended in 50 μl of PBS and mixed with matrigel (1:1), then were orthotopically injected into the mouse mammary fat pad following an established protocol (5–8 mice for each group). Mice were killed on day 25 after tail vein injection or day 28 after mammary fat pad injection. Tumor growth was determined by non-blinded weekly caliper measurements and tumor volumes were calculated using the following formula: (width × length^2 ^× π)/6. Metastasis in the lungs was detected by bioluminescence imaging (BLI). All lobes of the lung were harvested and the number of macroscopic metastatic nodules on the surface of the lung was counted. For in vivo drug treatment, 7 days after inoculation, tumor-bearing mice were treated with UNC0379 (25 mg/kg) or/and verteporfin (50 mg/kg) via intraperitoneal injection (i.p.) every day. Verteporfin was dissolved in DMSO (100 mg/ml), aliquoted, and stored at −80 °C. The working solution was prepared in PBS freshly before use, 21 days after injection, luciferin was injected and the primary/metastatic tumors were detected by BLI with the IVIS 100 (Caliper Life Sciences, Hopkinton, MA, USA). Animal survival was eventually analyzed. Besides, independent experimental groups used for corresponding survival analysis were performed under the same conditions. The mice were monitored daily and were euthanized upon reaching the criteria according to UCSD Institutional Animal Care and Use Committee guidelines. The survival data were analyzed by log-rank test. Data were pooled from independent experiments showing similar results. All mouse studies were conducted following protocols approved by the Animal Care and Use Committee of the Chinese Academy of Medical Science.

### Statistics and reproducibility

All co-IP and immunostaining experiments were repeated three times, with similar results detected each time. All statistical analyses were performed with GraphPad Prism version 8.3 (GraphPad Software Inc., San Diego, CA, USA). The significance of the data between experimental groups was determined by one-way analysis of variance (ANOVA) with Newman-Keuls post-test or unpaired two-tailed Student’s *t*-test. For clinicopathologic analysis, a chi-square test or Fisher exact test (two-sided) was performed. Survival analysis was calculated using the log-rank test and the Kaplan–Meier method. Statistical analyzed data are expressed as the mean ± SD or mean ± SEM, as indicated. A *P*-value < 0.05 represented a statistically significant difference. All the calculated *P* values were derived from at least three independent experiments with three technical replicates for each experiment. No statistical method was utilized to predetermine the sample size.

### Reporting summary

Further information on research design is available in the Nature Research Reporting Summary linked to this article.

## Supplementary information

Supplementary Information

Description of Additional Supplementary Information

Supplementary Data 1

Reporting Summary

## Data Availability

RNA-Seq data reported in this study have been deposited with the NCBI BioProject database (http://www.ncbi.nlm.nih.gov/bioproject) under Bioproject ID: PRJNA 797682. The mass spectrometry proteomics data have been deposited to the ProteomeXchange Consortium (http://proteomecentral.proteomexchange.org) with the dataset identifier PXD 032813. The data supporting the finding of this study are available within the article and its Supplementary Information files or available from the corresponding author on request. The source data underlying Figs. 1g, i, k, m, n, 3e–g, 4b–h, 5g, h, 6e–h, 7a– g, h, 8b, c, e, and Supplementary Figs. 1j, l, 3b, c, 4b–f, 5b, d, e, f, 6b–h are provided as a Source Data file. Source data are provided with this paper.

## Source data

Source Data
