# Supplementary material for: KMT5A-methylated SNIP1 promotes triple-negative breast cancer metastasis by activating YAP signaling
Source: Nat Commun. 2022 Apr 21;13:2192. doi: 10.1038/s41467-022-29899-w (PMC9023492; doi:10.1038/s41467-022-29899-w)
Supplement: Supplementary file 1 — Supplementary Information [file 41467_2022_29899_MOESM1_ESM.pdf]

Supplementary Figure and Figure legends

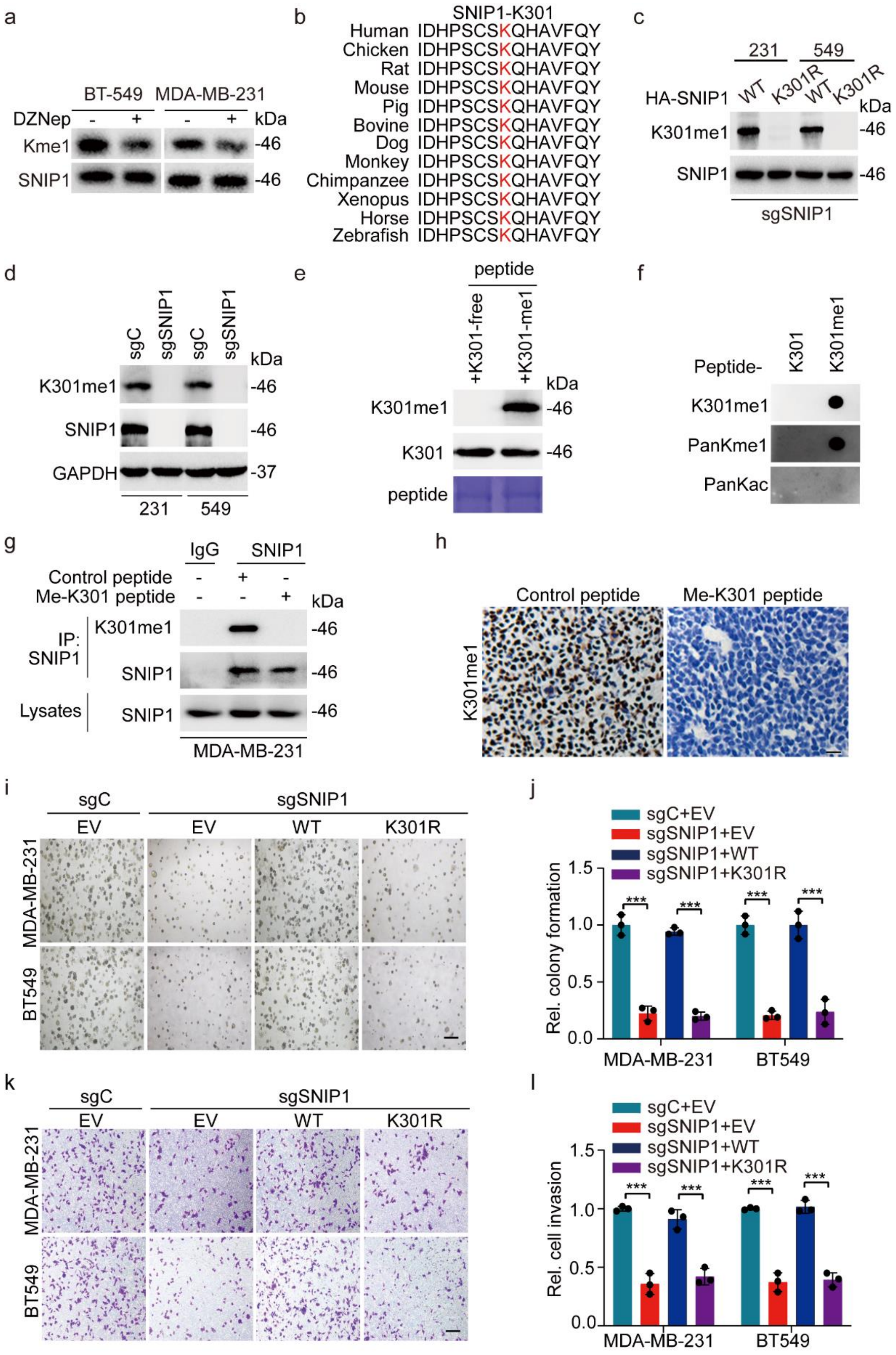

Supplementary Fig. 1 SNIP1 methylation identification, and it promotes cells colony formation and invasion.

a, Immunobloting (IB) analysis of SNIP1 immunoprecipitates (IP) products and whole cell lysates (WCL) derived from MDA-MB-231 and BT-549 cells treated with/without methyltransferase inhibitors DZNep (5 μM) 12 h before harvesting (n = 3).
b, Amino acid sequence alignment of SNIP1 among the indicated species showing Lys301, which is highlighted in red.
c, IB analysis of WCL derived from sgSNIP1 MDA-MB-231 and BT-549 cells infected with indicated $SNIP1^{WT}$ or $SNIP1^{K301R}$ and selected with hygromycin (200 μg/ml) for 72 h before collection. A rabbit anti-SNIP1-K301me1 antibody was generated against a specific methyl-peptide containing me-K301 (n = 3).
d, IB analysis of K301me1 and SNIP1 in wild type and SNIP1 knockout MDA-MB-231 and BT549 cells, as indicated (n = 3).
e and **f**, IB (**e**) and dot blot analysis (**f**) of SNIP1 K301 mono-methylation antibody with using K301 unmodified (K301-free) and K301 monomethylated (K301-me1) peptides. **e**, Top panel, IB analysis; lower panel, Coomassie brilliant blue staining. **f**, Dot blot analysis of K301me1, mono-methyllysine and pan-Kme1 for the peptide containing methylated K301 (n = 3).
g, IP and WB for SNIP1 methylation in MDA-MB-231 cells. Before IP, agarose beads were pre-incubated with a control peptide or the specific methyl-peptide containing me-K301 (n = 3).
h, Immunohistochemistry (IHC) assays of a clinical breast cancer tissue with the specific anti-SNIP1-K301me1 antibody in the presence of a control peptide or the specific methyl-peptide containing me-K301. IHC was performed twice on the breast cancer sample with the blocking peptide with similar results. Scale bar, 50μm (n = 3).
i and **j**, Cells derived from MDA-MB-231/sgSNIP1 cells re-expressed with indicated SNIP1-WT or SNIP1-K301R and selected with hygromycin (200 μg/ml) for 72 h before collection were subjected to colony-formation assay (**i**). Representative images are shown in **i**, and relative colony numbers are plotted in **j**. Scale bars, 250 μm (n = 3).
k and **l**, Cells generated in **i** were subjected to transwell assays (**k**) (n = 3). Representative images are shown in **k**, and relative invasion numbers are calculated in **l**. Scale bars, 50 μm.
Data information: In (**j**, **l**), data are expressed as the mean ± SD. ****P* <0.001, by two-tailed t-test. Panels (**a**, **c-l**) show 1 representative of 3 independent experiments with similar results.

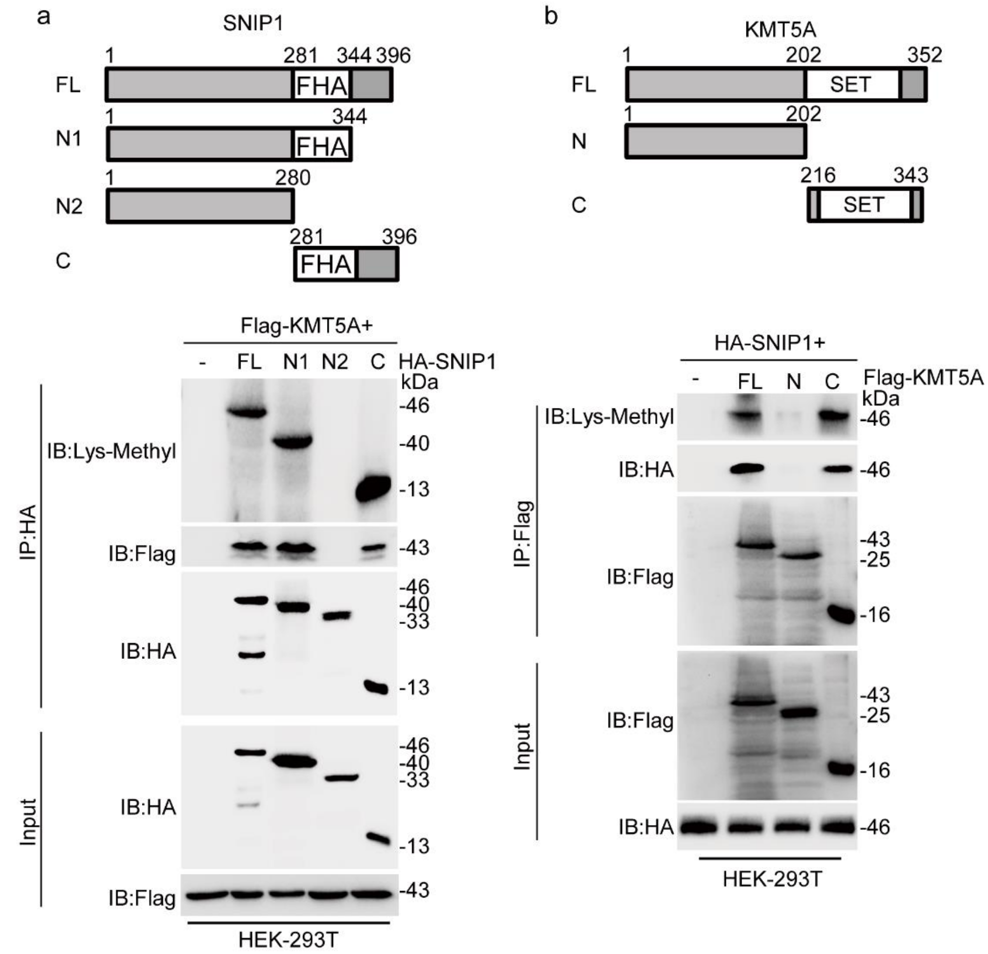

Supplementary Fig. 2 KMT5A interacts with SNIP1 directly.

a, Upper, schematics of SNIP1 full length (FL, 1-396aa), N1 (N-terminal, 1-344aa, contain FHA domain) mutant, N2 (N-terminal, 1-280aa) mutant, and C (C-terminal, 281-396aa, contain FHA domain) mutant plasmids; bottom, IB detection of SNIP1 methylation was immunoprecipitated with anti-HA magnetic beads in HEK-293T cells transfected with the Flag-KMT5A and HA-SNIP1 constructs (n = 3).

b, Upper, schematics of KMT5A full length (FL, 1-352aa), N (N-terminal, 1-202aa) mutant, and C (C-terminal, 216-343aa, contain SET domain) mutant plasmids; bottom, IB detection of SNIP1 methylation was immunoprecipitated with anti-Flag M2 beads in HEK-293T cells transfected with the HA-SNIP1 and Flag-KMT5A constructs (n = 3).

Data information: Panels (**a**) and (**b**) show 1 representative of 3 independent experiments with similar results.

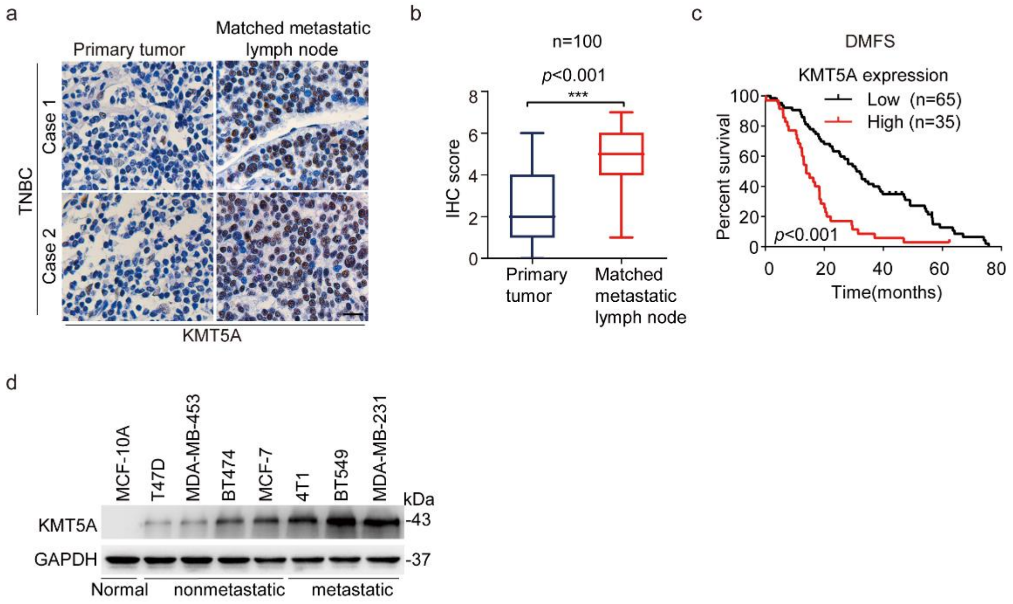

Supplementary Fig. 3 KMT5A is associated with triple negative breast cancer metastasis.

a, Representative IHC staining of KMT5A in primary TNBC and matched metastatic lymph nodes. Scale bars: 50 μm.

b, Quantitative analysis of KMT5A IHC scores in (**a**) (n=100 paired samples). All Box and whisker plots represent the median (central line), 25th-75th percentile (bounds of the box) and 5th-95th percentile (whiskers).

c, High expression of KMT5A protein in primary TNBC tumor tissues associated with poor distance metastasis free survival (DMFS) in our cohort (n= 100).

d, IB analysis of KMT5A in the normal breast epithelium cell line and breast cancer cell lines with different metastatic ability (n = 3).

Data information: In (**b**), data are presented as mean ± SEM, ***P<0.001, by two-tailed t-test. In (**c**), statistical analysis was performed by log-rank test, $P < 0.001$. Panel (**d**) shows 1 representative of 3 independent experiments with similar results.

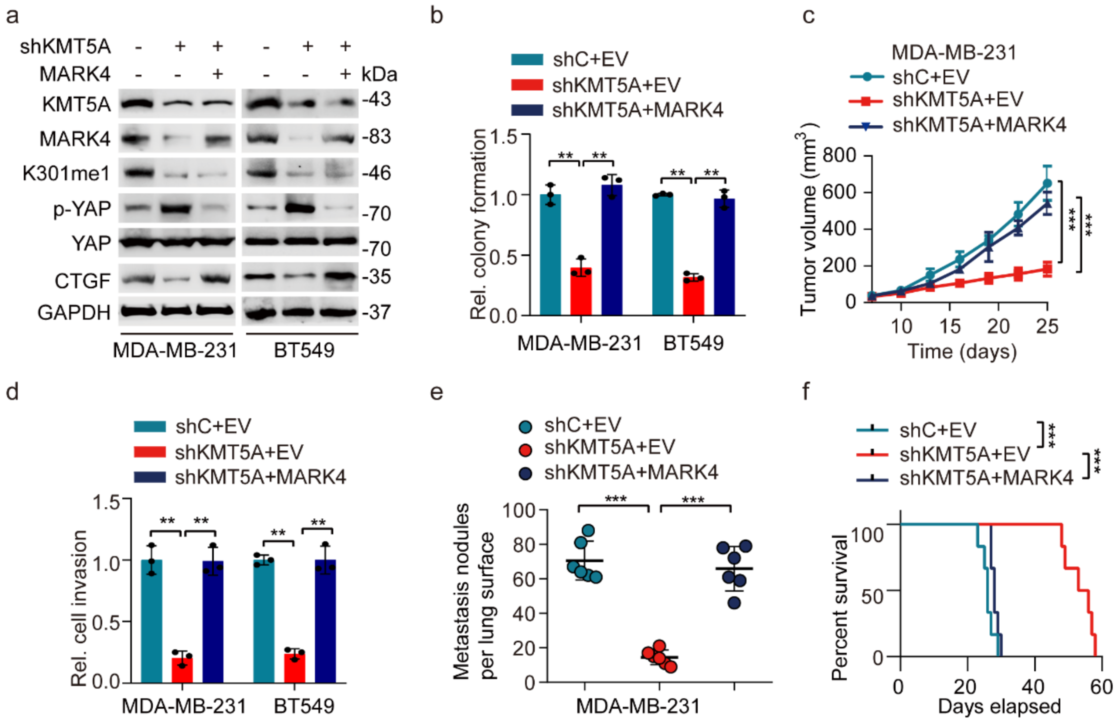

Supplementary Fig. 4 KMT5A mediated-SNIP1 methylation to activate Hippo/YAP signaling, cell proliferation and invasion through regulating MARK4.
a, IB analysis of ectopic expression of KMT5A combined with MARK4 knockdown on Hippo signaling activation in MDA-MB-231 and BT549 cells (n = 3).
b, Cells generated in **a** were subjected to colony-formation assay and relative colony numbers are plotted (n = 3).
c, Cells generated in **a** were subjected to mouse xenograft assays by orthotopic injection in athymic nude mice, and tumor sizes were monitored and analyzed (n = 6 mice per group).
d, Cells generated in **a** were subjected to transwell assays and relative invasion numbers are calculated (n = 3).
e-f, Effects of ectopic expression of MARK4 combined with KMT5A knockdown on cell lung metastasis (**e**), and mouse lifespan (**f**), which these cells generated in **a** were implanted into the lateral tail vein of athymic nude mice (n=6 mice per group).
Data information: In (**b**-**f**), statistical analysis was performed by two-tailed t-test. In (**f**), by log-rank test. Error bars ± S.E.M. $^{**}P$ <0.01, $^{***}P$ <0.001. Panels (**a**, **b**, **d**) show 1 representative of 3 independent experiments with similar results. Panels (**c**, **e**, **f**) show 1 representative of 2 independent experiments with similar results.

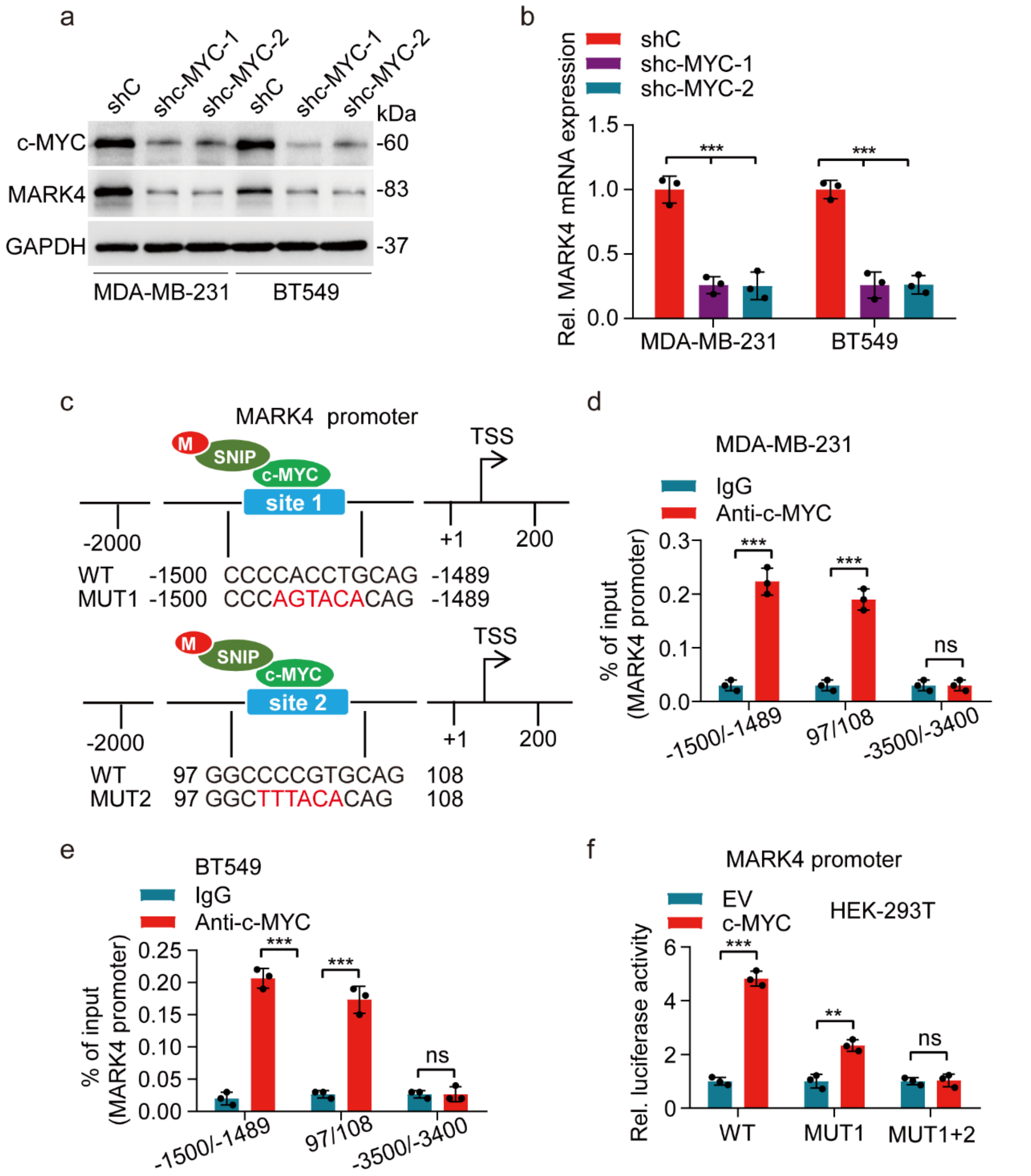

Supplementary Fig. 5 c-MYC regulates MARK4 transcription.

a, c-MYC knockdown impaired MARK4 expression in MDA-MB-231 and BT549 cells (n = 3).

b, qRT–PCR analysis of the effect of c-MYC knockdown on MARK4 expression in MDA-MB-231 and BT549 cells (n = 3).

c, Schematic diagram of putative c-MYC-binding sites in MARK4 promoter.

d and **e**, ChIP-qPCR analysis of the binding of c-MYC with MARK4 promoter in MDA-MB-231 (**d**) and BT549 (**e**) cells. An anti-IgG or anti-c-MYC antibody was used (n = 3).

f, Luciferase activity in HEK-293T cells co-transfected with c-MYC and luciferase reporters containing MARK4 promoter WT, mutants, or empty vector (EV) (n = 3).

Data information: In (**b-f**), data are expressed as the mean ± SD. ***P*<0.01, ****P* <0.001, by two-tailed t-test. Panels (**a**, **b**, **d-f**) show 1 representative of 3 independent experiments with similar results.

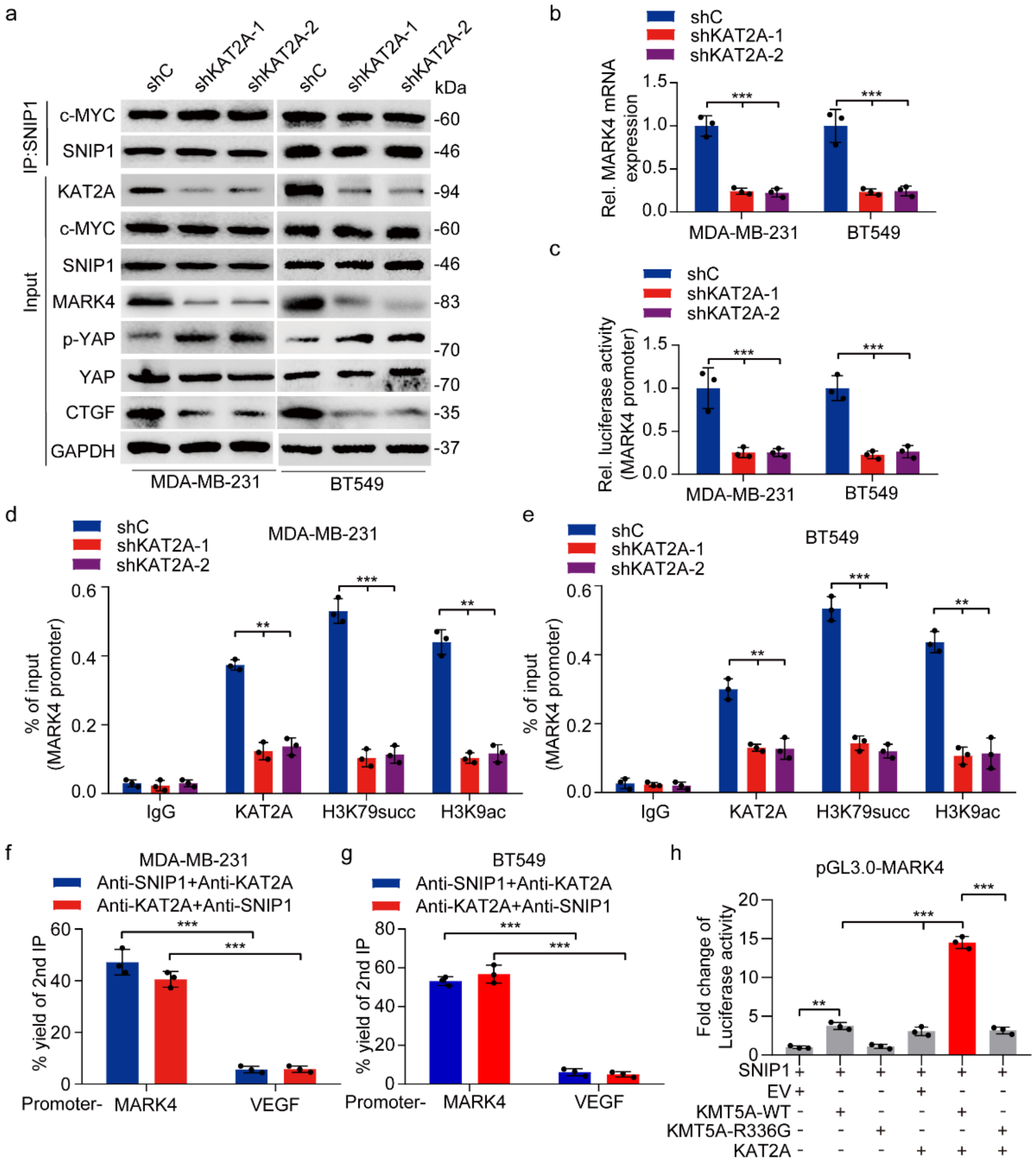

Supplementary Fig. 6 KMT5A-mediated SNIP1 methylation interacts with KAT2A to activate MARK4 transcription.

a, Immunoprecipitation and western blotting for KAT2A knockdown on SNIP1 binding with c-MYC protein, MARK4 expression and YAP signaling activation (n = 3).

b, MARK4 mRNA expression in *KAT2A* knockdown cells. Nontargeting shRNA (shC). KAT2A knockdown (shKAT2A-1 and shKAT2A-2) (n = 3).

c, Luciferase activity of MARK4 promoter in KAT2A knockdown cells in **b** (n = 3).

d and **e**, ChIP-qPCR results showing decreased KAT2A, H3K79succ and H3K9ac levels at the MARK4 promoter after *KAT2A* silencing in MDA-MB-231 (**d**) and BT549 (**e**) cells (n = 3).

f and **g**, Sequential ChIP-qPCR for analyzing the co-occupancy of SNIP1 and KAT2A on the promoter of MARK4 and VEGF in MDA-MB-231 (**f**) and BT549 (**g**) cells. Data represent yields of secondary immunoprecipitation (n = 3).

h, Luciferase (luc) reporter assay. The HEK-293T cells were co-transfected with the reporter plasmid containing MARK4 (pGL3.0-MARK4) promoter and different expression vectors as indicated (n = 3).

Data information: In (**b**-**h**), data are expressed as the mean ± SD. ***P*<0.01, ****P* <0.001, by two-tailed t-test. Panels (**a**-**h**) show 1 representative of 3 independent experiments with similar results.

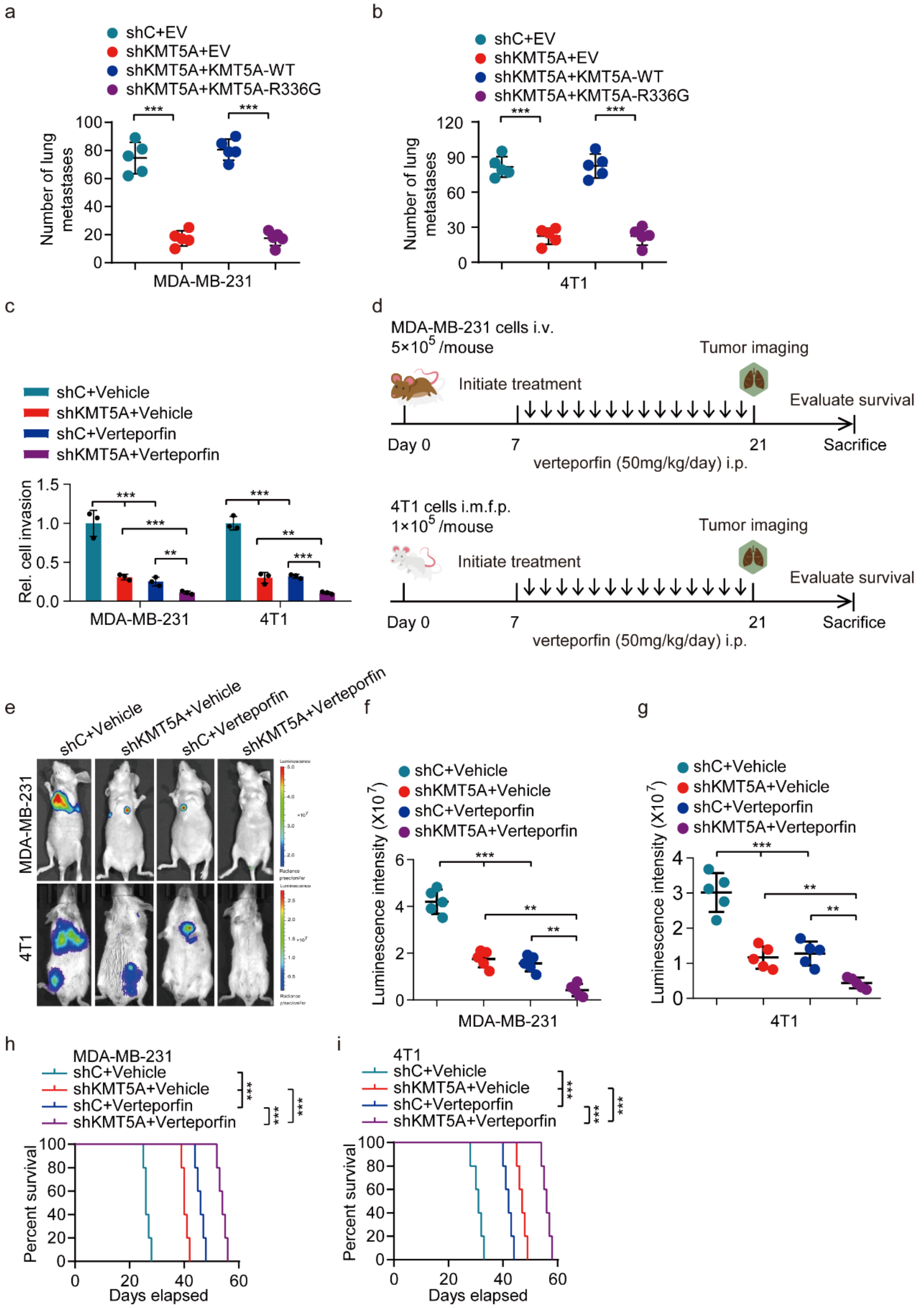

Supplementary Fig. 7 KMT5A catalytic activity depletion combined with YAP signaling inhibition impedes triple-negative breast cancer progression.

a and **b**, Scatter blot showing lung metastatic nodules for MDA-MB-231 (**a**) and 4T1 (**b**) xenografts modified to express shRNA KMT5A or shRNA control and re-expressing shRNA resistant KMT5A^{WT} or catalytically deficient KMT5A^{R336G} in mice (n = 5 mice for each group). Nontargeting shRNA (shC). Empty vector (EV).

c, Invasion analysis of MDA-MB-231 and 4T1 cell lines depleted for KMT5A or control. Cells were treated with verteporfin (10 µM) or placebo (vehicle) as indicated (n = 3).
d, Treatment schedules for the administration of verteporfin (50 mg/kg, intraperitoneal injection once daily) to mice grafted with MDA-MB-231 cells (upper) or 4T1 cells (lower). Control mice received placebo (vehicle). Intravenous injection (i.v.). Orthotopic mammary fat pad injection (i.m.f.p.). Intraperitoneal injection (i.p.). (n = 5 mice per group).
e, Representative bioluminescence images in **d** on day 21.
f and **g**, Quantification of the bioluminescence activity of MDA-MB-231 (**f**) and 4T1 (**g**) tumor xenografts from verteporfin treated and control mice in (**e)**. (n = 5 mice per group).
h and **i**, Kaplan-Meier survival analysis of mice with MDA-MB-231 (**h**) and 4T1 (**i**) tumor xenografts (n = 5 mice per group).
Data information: In (**a** and **b**), statistical analysis was performed by two-tailed t-test. In (**c, f** and **g**), by two-tailed Student's t-test or one-way ANOVA. In (**h** and **i**), by log-rank test. ***P <0.001, **P <0.01. Data are represented as mean ± S.E.M. Panels (**a-c**, **e-i**) show 1 representative of 3 independent experiments with similar results.

Supplementary Table 1 Primers for qRT-PCR assays and ChIP-qPCR assays.

Primer pairs	Sequence
qRT-PCR, GAPDH	5’-GGAGCGAGATCCCTCCAAAAT-3’ and 5’-GGCTGTTGTCATACTTCTCATGG-3’
qRT-PCR, DAB2	5’-GTAGAAACAAGTGCAACCAATGG-3’ and 5’-GCCTTTGAACCTTGCTAAGAGA-3’
qRT-PCR, CDC20	5’-GCACAGTTCGCGTTCGAGA-3’ and 5’-CTGGATTTGCCAGGAGTTCGG-3’
qRT-PCR, HMMR	5’-ATGATGGCTAAGCAAGAAGGC-3’ and 5’-TTTCCCTTGAGACTCTTCGAGA-3’
qRT-PCR, TK1	5’-GGGCAGATCCAGGTGATTCTC-3’ and 5’-TGTAGCGAGTGTCTTTGGCATA-3’
qRT-PCR, ECT2	5’-ACTACTGGGAGGACTAGCTTG-3’ and 5’-CACTCTTGTTTCAATCTGAGGCA-3’
qRT-PCR, SH2D4A	5’-CTGGAGCAAGGATCGAGGC-3’ and 5’-CAGCTCTTACAAATCTGCTTCGT-3’
qRT-PCR, TSPAN3	5’-GAGTGTCCCTCTTAGCTGCTG-3’ and 5’-AGCTTCTTCACTACTAGAGCCTC-3’
qRT-PCR, CRIM1	5’-CCCTGTGACGAGTCCAAGTG-3’ and 5’-GGTTCCGTAAATCCCGAAGGT-3’
qRT-PCR, DCL1	5’-TGGAGCGGACATGATAAGCAT-3’ and 5’-AGCACAGGTGTCAACTAAATCC-3’
qRT-PCR, AXL	5’-GTGGGCAACCCAGGGAATATC-3’ and 5’-GTACTGTCCCGTGTCGGAAAG-3’
qRT-PCR, SLIT2	5’-GCGAAGCTATACAGGCTTGAT-3’ and 5’-TGCAGTCGAAAAGTCCTAAGTTT-3’
qRT-PCR, LHFP	5’-CTCCTGCGTGGGGTTCTTTAT-3’ and 5’-CCGGTCACTATGGTGCAGAT-3’
qRT-PCR, CENPF	5’-CTCTCCCGTCAACAGCGTTC-3’ and 5’-GTTGTGCATATTCTTGGCTTGC-3’
qRT-PCR, FLNA	5’-CTTATCGCGCTGTTGGAGGT-3’ and 5’-GCCACCGACACGTTCTCAA-3’
qRT-PCR, NDRG1	5’-CTCCTGCAAGAGTTTGATGTCC-3’ and 5’-TCATGCCGATGTCATGGTAGG-3’
qRT-PCR, YAP	5’-TAGCCCTGCGTAGCCAGTTA-3’ and 5’-TCATGCTTAGTCCACTGTCTGT-3’
qRT-PCR, CTGF	5’-CAGCATGGACGTTCGTCTG-3’ and 5’-AACCACGGTTTGGTCCTTGG-3’
qRT-PCR, CYR61	5’-CTCGCCTTAGTCGTCACCC-3’ and 5’-CGCCGAAGTTGCATTCCAG-3’
qRT-PCR, HIF-1α	5’-GAACGTCGAAAAGAAAAGTCTCG-3’ and 5’-CCTTATCAAGATGCGAACTCACA-3’
qRT-PCR, VEGF	5’-AGGGCAGAATCATCACGAAGT-3’ and 5’-AGGGTCTCGATTGGATGGCA-3’

qRT-PCR, JAK-1	5’-CTTTGCCCTGTATGACGAGAAC-3’ and 5’-ACCTCATCCGGTAGTGGAGC-3’
qRT-PCR, JAK-2	5’-TCTGGGGAGTATGTTGCAGAA-3’ and 5’-AGACATGGTTGGGTGGATACC-3’
qRT-PCR, JAK-3	5’-TTCGGGCTACGCAAGGATTTG-3’ and 5’-AGGCTGAGACACTCACCCT-3’
qRT-PCR, STAT3	5’-CAGCAGCTTGACACACGGTA-3’ and 5’-AAACACCAAAGTGGCATGTGA-3’
qRT-PCR, PIK3CA	5’-CCACGACCATCATCAGGTGAA-3’ and 5’-CCTCACGGAGGCATTCTAAAGT-3’
qRT-PCR, AKT	5’-AGCGACGTGGCTATTGTGAAG-3’ and 5’-GCCATCATTCTTGAGGAGGAAGT-3’
qRT-PCR, SNIP1	5’-TGAAGCAGGAGCGTCTCAG-3’ and 5’-TCGGTTTCTCTTACTGCGAGG-3’
qRT-PCR, MARK4	5’-AGGTTGCCATCAAGATTATCGAC-3’ and 5’-GATGCGGACTTCTCGGAACAG-3’
ChIP-qPCR, MARK4 promoter -50 to 150	5’-TTCGTGTCTCTCTATCTCTA-3’ and 5’-AAAAGCTGGGCGCCGAGAA-3’
ChIP-qPCR, MARK4 promoter -1550 to -1350	5’-GCAGTGAGACCCTGTCTCAA-3’ and 5’-TCTGTGATCTTGAGGTTACC-3’
