# Supplementary material for: KMT5A-methylated SNIP1 promotes triple-negative breast cancer metastasis by activating YAP signaling
Source: Nat Commun. 2022 Apr 21;13:2192. doi: 10.1038/s41467-022-29899-w (PMC9023492; doi:10.1038/s41467-022-29899-w)
Supplement: Supplementary file 2 — Description of Additional Supplementary Information [file 41467_2022_29899_MOESM2_ESM.pdf]

Description of Additional Supplementary Files

KMT5A-methylated SNIP1 promotes breast cancer metastasis by activating YAP signalling

Bo Yu, Jun Su, Qiqi Shi, Qing Liu, Jun Ma, Guoqing Ru, Lei Zhang, Jian Zhang, Xichun Hu, Jianming Tang

Figure S1-S7
Table S1
