# Supplementary material for: KMT5A-methylated SNIP1 promotes triple-negative breast cancer metastasis by activating YAP signaling
Source: Nat Commun. 2022 Apr 21;13:2192. doi: 10.1038/s41467-022-29899-w (PMC9023492; doi:10.1038/s41467-022-29899-w)
Supplement: Supplementary file 4 — Reporting Summary [file 41467_2022_29899_MOESM4_ESM.pdf]

nature portfolio

Corresponding author(s): Jianming Tang

Last updated by author(s): Mar 10, 2022

Reporting Summary

Nature Portfolio wishes to improve the reproducibility of the work that we publish. This form provides structure for consistency and transparency in reporting. For further information on Nature Portfolio policies, see our Editorial Policies and the Editorial Policy Checklist.

Statistics

For all statistical analyses, confirm that the following items are present in the figure legend, table legend, main text, or Methods section.

n/a	Confirmed	
☐	☒	The exact sample size (*n*) for each experimental group/condition, given as a discrete number and unit of measurement
☐	☒	A statement on whether measurements were taken from distinct samples or whether the same sample was measured repeatedly
☐	☒	The statistical test(s) used AND whether they are one- or two-sided<br>*Only common tests should be described solely by name; describe more complex techniques in the Methods section.*
☐	☒	A description of all covariates tested
☐	☒	A description of any assumptions or corrections, such as tests of normality and adjustment for multiple comparisons
☐	☒	A full description of the statistical parameters including central tendency (e.g. means) or other basic estimates (e.g. regression coefficient) AND variation (e.g. standard deviation) or associated estimates of uncertainty (e.g. confidence intervals)
☐	☒	For null hypothesis testing, the test statistic (e.g. *F*, *t*, *r*) with confidence intervals, effect sizes, degrees of freedom and *P* value noted<br>*Give P values as exact values whenever suitable.*
☒	☐	For Bayesian analysis, information on the choice of priors and Markov chain Monte Carlo settings
☒	☐	For hierarchical and complex designs, identification of the appropriate level for tests and full reporting of outcomes
☐	☒	Estimates of effect sizes (e.g. Cohen's *d*, Pearson's *r*), indicating how they were calculated

Our web collection on statistics for biologists contains articles on many of the points above.

Software and code

Policy information about availability of computer code

Data collection	*Illumina HiSeqX Ten platforms<br>* Q ExactiveTM Plus (Thermo) coupled online
Data analysis	*DAVID<br>*MASCOT<br>*GraphPad Prism version 8.3<br>SPSS Statistics 20.0

For manuscripts utilizing custom algorithms or software that are central to the research but not yet described in published literature, software must be made available to editors and reviewers. We strongly encourage code deposition in a community repository (e.g. GitHub). See the Nature Portfolio guidelines for submitting code & software for further information.

Data

Policy information about availability of data

All manuscripts must include a data availability statement. This statement should provide the following information, where applicable:

- Accession codes, unique identifiers, or web links for publicly available datasets
- A description of any restrictions on data availability
- For clinical datasets or third party data, please ensure that the statement adheres to our policy

Source data are provided with this paper. RNA-Seq data reported in this study have been deposited with the NCBI BioProject database (http://www. ncbi.nlm.nih. gov/bioproject) under Bioproject ID: PRJNA797682. The datasets generated or analyzed during current study available from the corresponding author (ldyy_tangjm@lzu.edu.cn) on reasonable request.

March 2021

Field-specific reporting

Please select the one below that is the best fit for your research. If you are not sure, read the appropriate sections before making your selection.

☒ Life sciences ☐ Behavioural & social sciences ☐ Ecological, evolutionary & environmental sciences

For a reference copy of the document with all sections, see nature.com/documents/nr-reporting-summary-flat.pdf

Life sciences study design

All studies must disclose on these points even when the disclosure is negative.

Sample size	Sample sizes were indicated in the legend of each Figure and Supplementary Figure. No statistical methods were used to predetermine sample sizes. We determined sample sizes as follows; for cell culture experiments, we performed at least triplicate experiments and for animal experiments, at least n=6 for each treatment group, to meet the minimal requirements for statistical analysis.
Data exclusions	No data were excluded from the analyses.
Replication	All experiments were replicated at least three times . All attempts to replication were successful.
Randomization	All animals, all cells were maintained in the same environment and were randomly assigned to the experimental groups.
Blinding	For data automatically collected by instruments, such as RNA-seq analysis, researchers were not blinded as observer bias is expected not to affect the results. Investigators performing histopathological analyses were blinded to the genotype. For all cell biological experiments no blinding was performed due to the limited availability of persons performing the experiments.

Reporting for specific materials, systems and methods

We require information from authors about some types of materials, experimental systems and methods used in many studies. Here, indicate whether each material, system or method listed is relevant to your study. If you are not sure if a list item applies to your research, read the appropriate section before selecting a response.

Materials & experimental systems

n/a	Involved in the study	
☐	☒	Antibodies
☐	☒	Eukaryotic cell lines
☒	☐	Palaeontology and archaeology
☐	☒	Animals and other organisms
☒	☐	Human research participants
☒	☐	Clinical data
☒	☐	Dual use research of concern

Methods

n/a	Involved in the study	
☒	☐	ChIP-seq
☒	☐	Flow cytometry
☒	☐	MRI-based neuroimaging

Antibodies

Antibodies used	Antibodies for KMT5A (#2996), GAPDH (#5174), MARK4 (#4834), VEGF (#50661), MST2 (#3952), p-MST2 (Thr180, #49332), YAP (#14074), p-YAP (Ser127, #13008), SAV1 (#13301), Phospho-Ser/Thr (#9631), LATS1 (#3477), CTGF (#10095), Mono-Methyl Lysine (#14679), HA (#3724), His (#12698), KAT2A (#3305), c-Myc (#18583) , VEGF (#50661) and mono-methyl lysine (#14679) were from CST (Danvers, MA);<br>An antibody for SNIP1 K301 mono-methylation rabbit polyclonal antibody generated by Proteintech using the peptide IDHPSCS-K(me)-QHAVFQY;<br>An antibody for FLAG (F3165) was from Sigma-Aldrich;<br>Antibodies for GAPDH (sc-25778) and GST (sc-138) were from Santa Cruz Biotechnology (Santa Cruz, CA);<br>Antibodies for Phospho-Ser/Thr (ab17464), SNIP1 (ab126194), acetyl-Histone H3 (Lys9) (ab4441), and V5-tag (ab9113) were purchased from Abcam (UK);<br>An antibody for succinyl-Histone H3 (Lys79) (PTM-412) was from PTM Biolabs;<br>The secondary antibodies, anti-rabbit IgG, HRP-linked antibody (#7074) and anti-mouse IgG, HRP-linked antibody (#7076) were purchased from CST (Danvers, MA).
Validation	All antibodies were validated by western blotting.

Eukaryotic cell lines

Policy information about cell lines

Cell line source(s)	MCF10A, T47D, MDA-MB-453, BT474, MCF7, BT549, MDA-MB-231, and HEK-293T cell lines were purchased from the Chinese National Infrastructure of Cell Line Resource (Beijing, China). The 4T1 cell line was obtained from the American Type Culture Collection (ATCC).
Authentication	All human cell lines were authenticated before the start of experiments using STR DNA fingerprinting at Shanghai Biowing Applied Biotechnology Co., Ltd. (Shanghai, China).
Mycoplasma contamination	All cell lines were tested negatively for mycoplasma contamination.
Commonly misidentified lines (See ICLAC register)	No common misidentified lines were used.

Animals and other organisms

Policy information about studies involving animals; ARRIVE guidelines recommended for reporting animal research

Laboratory animals	4-5 weeks-old pathogen-free female BALB/c and athymic nude mice were purchased from the Slaccas (Shanghai).
Wild animals	The study did not involve wild animals.
Field-collected samples	The study did not involve field-collected samples.
Ethics oversight	The mice were monitored daily and were euthanized upon reaching the criteria according to UCSD Institutional Animal Care and Use Committee guidelines. All mouse studies were conducted following protocols approved by the Animal Care and Use Committee of the Chinese Academy of Medical Science.

Note that full information on the approval of the study protocol must also be provided in the manuscript.
