# Supplementary material for: KMT5A-methylated SNIP1 promotes triple-negative breast cancer metastasis by activating YAP signaling
Source: Nat Commun. 2022 Apr 21;13:2192. doi: 10.1038/s41467-022-29899-w (PMC9023492; doi:10.1038/s41467-022-29899-w)

Immunoblot images depicted in Fig. 1d

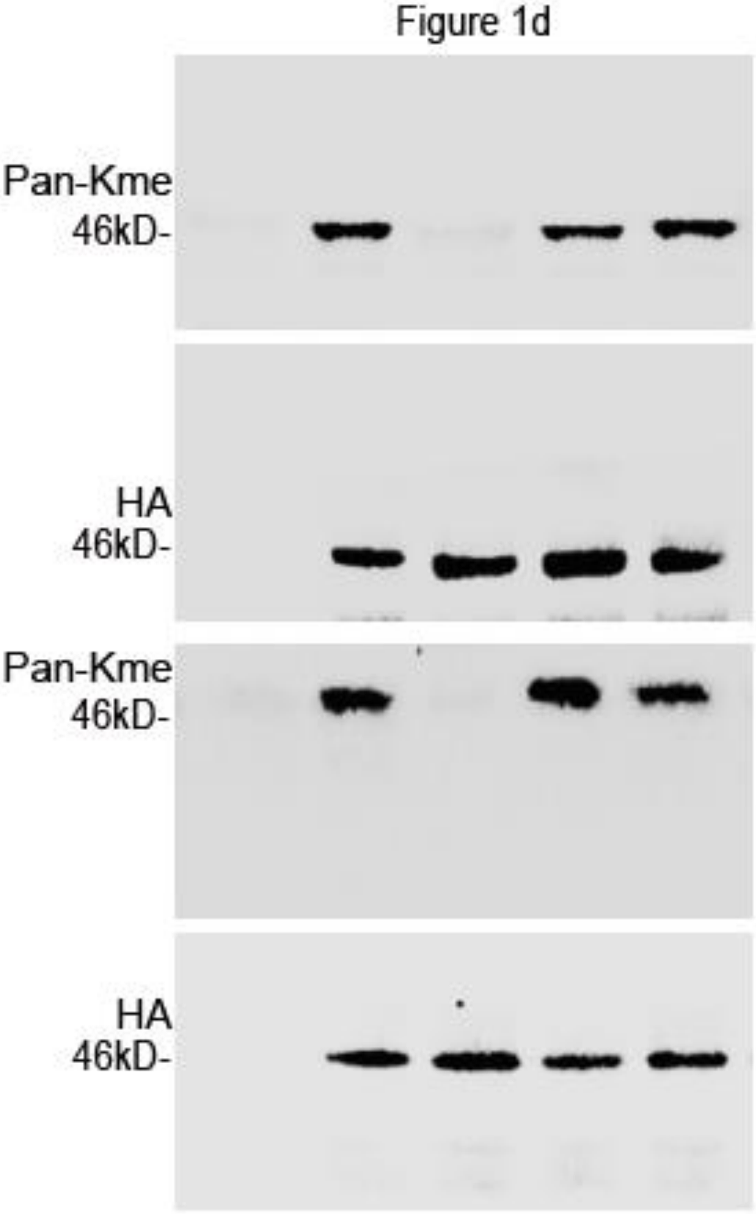

Immunoblot images depicted in Fig. 2b and 2c

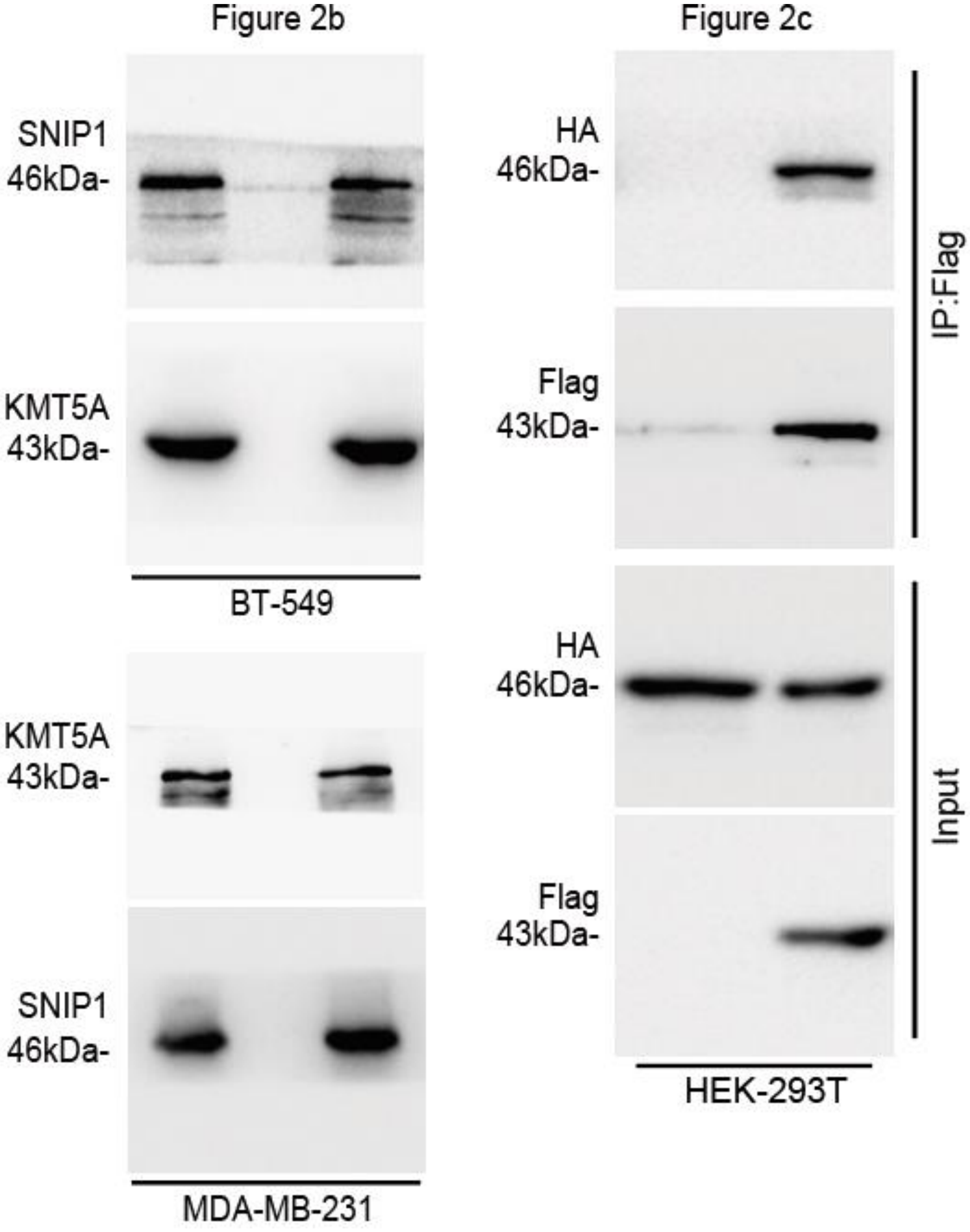

Immunoblot images depicted in Fig. 2d and 2e

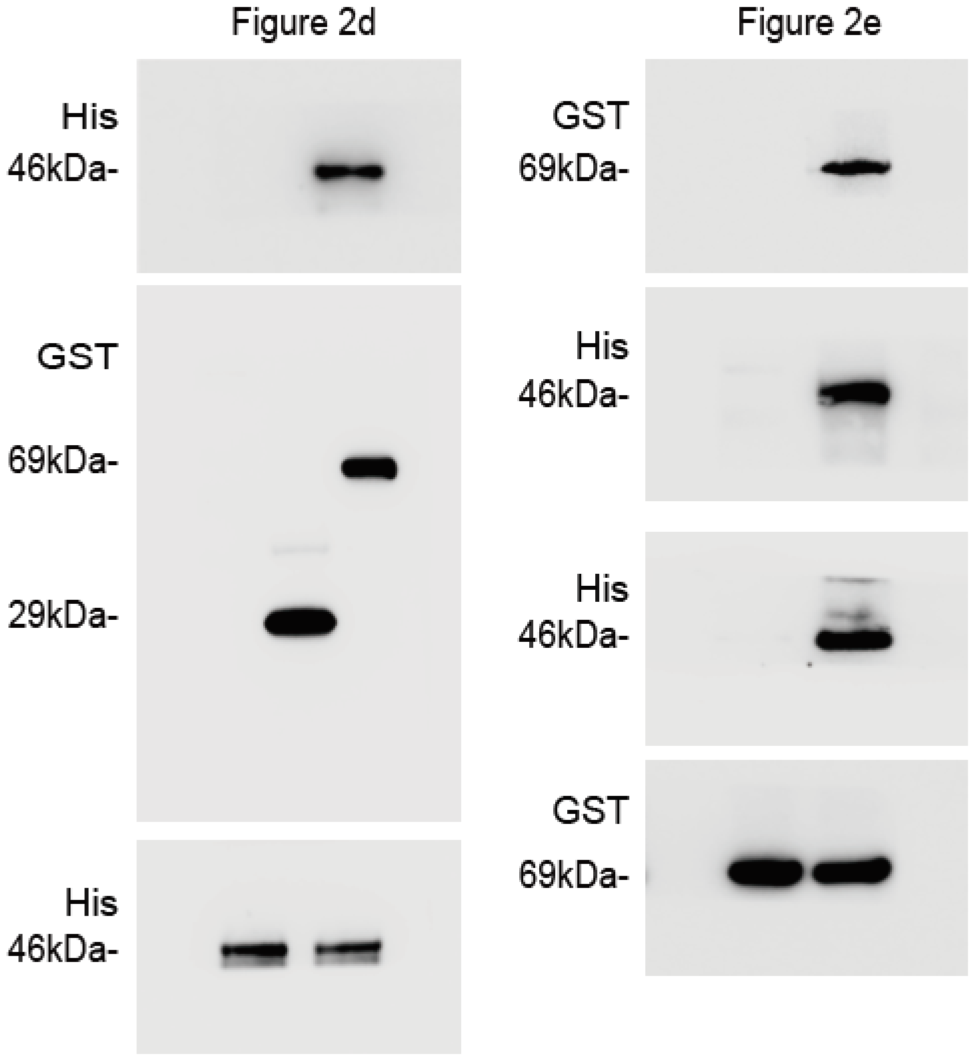

Immunoblot images depicted in Fig. 2f and 2g

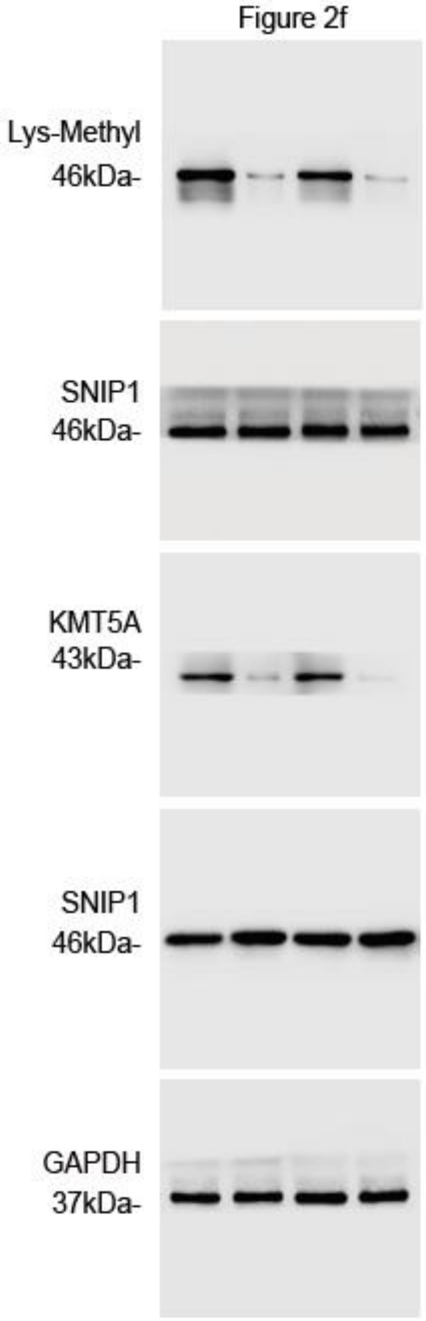

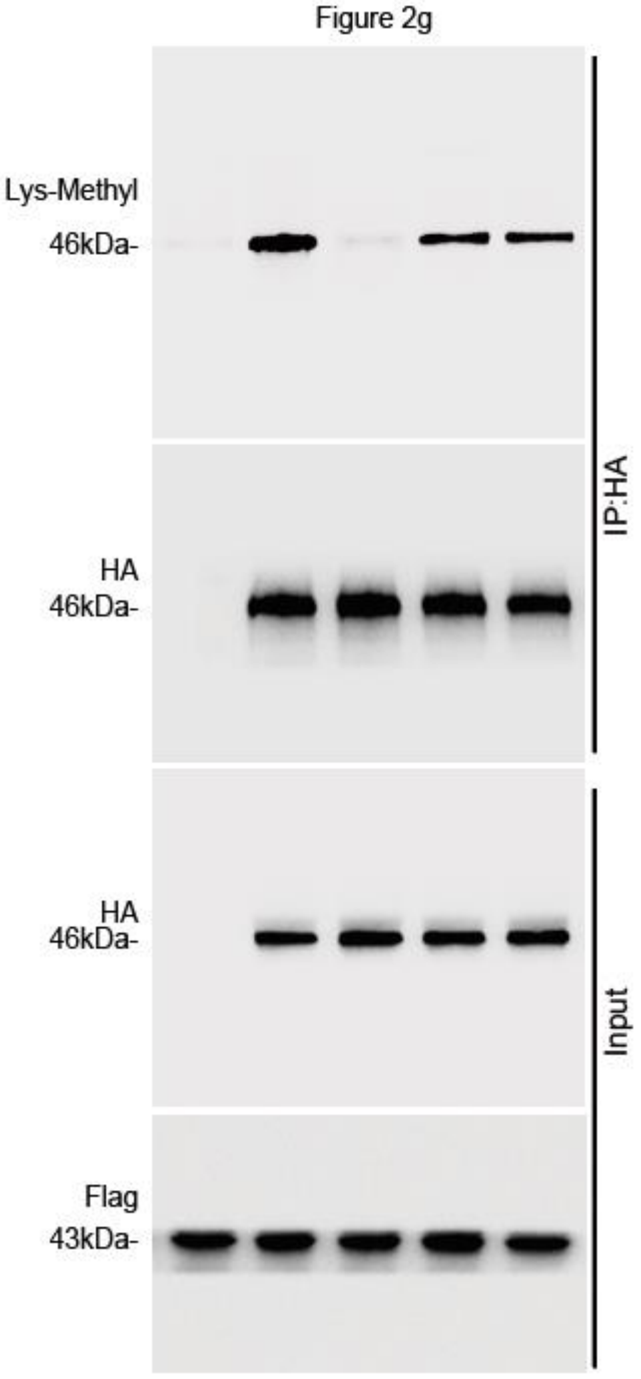

Immunoblot images depicted in Fig. 2h

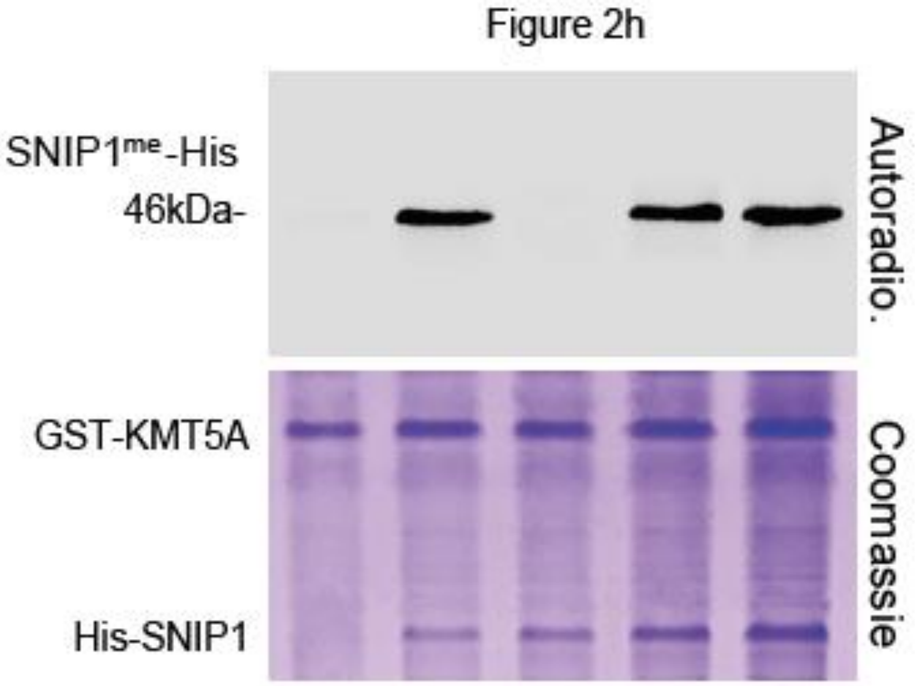

Immunoblot images depicted in Fig. 2i, 2j, and 2k

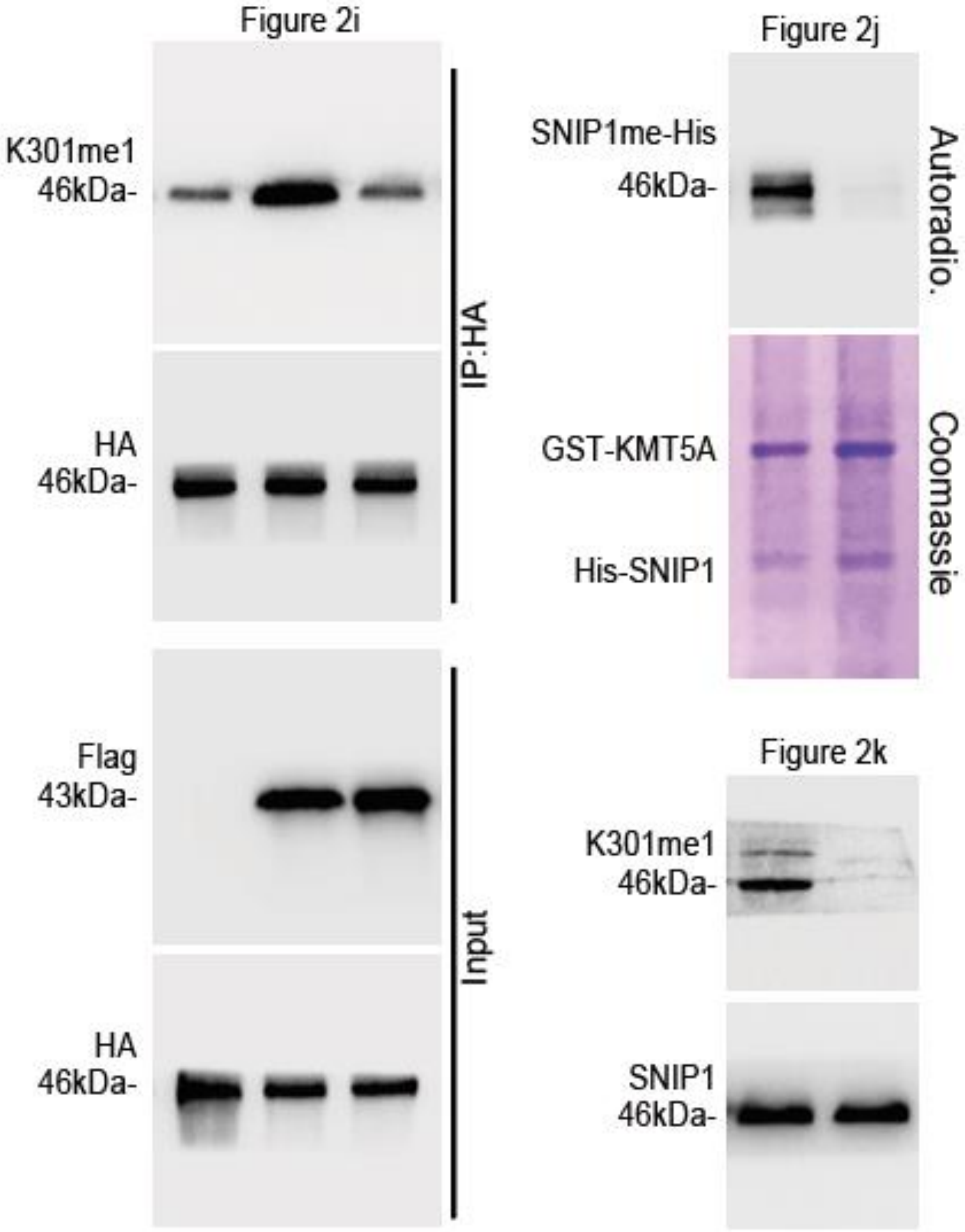

Immunoblot images depicted in Fig. 3h

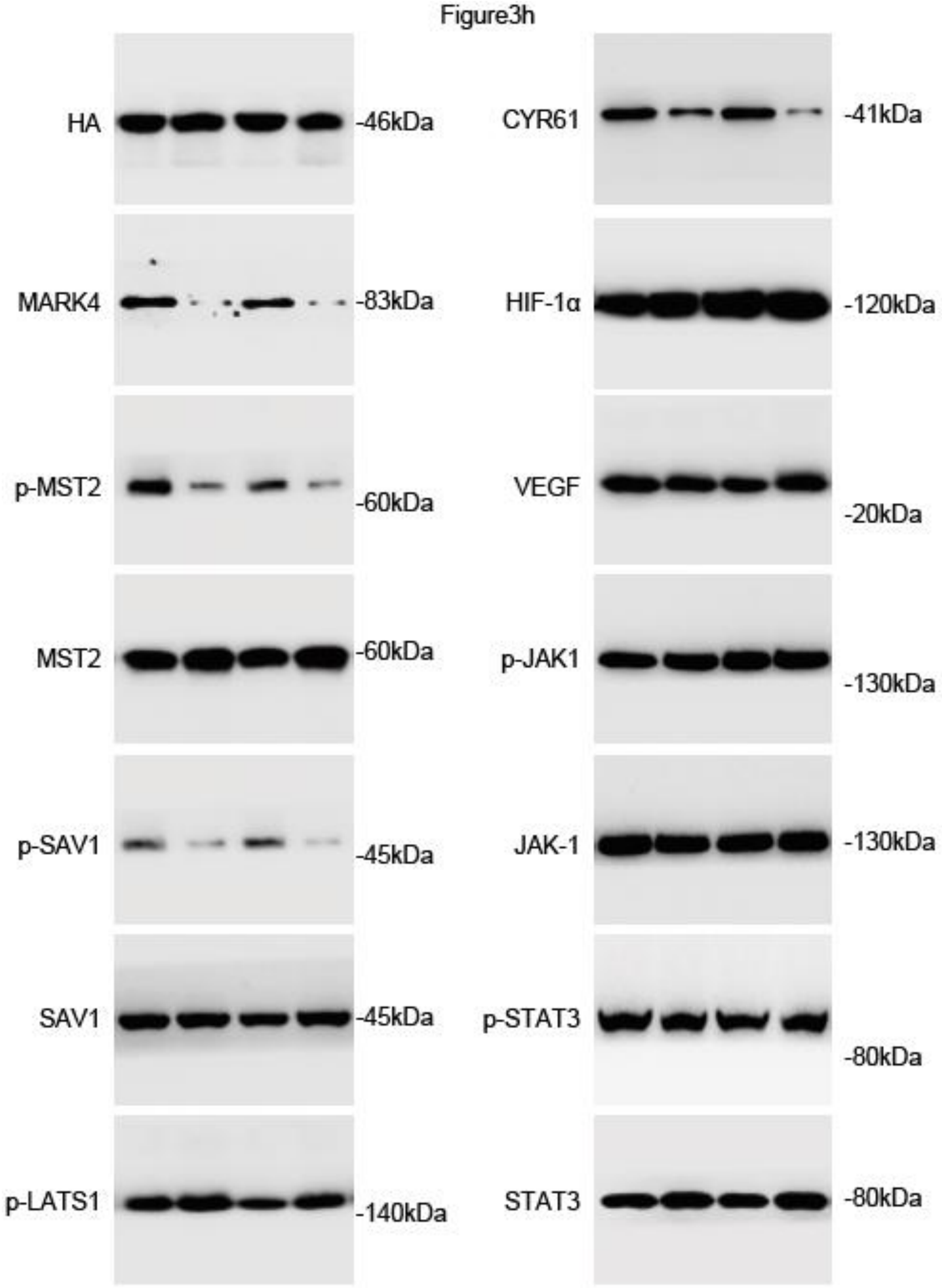

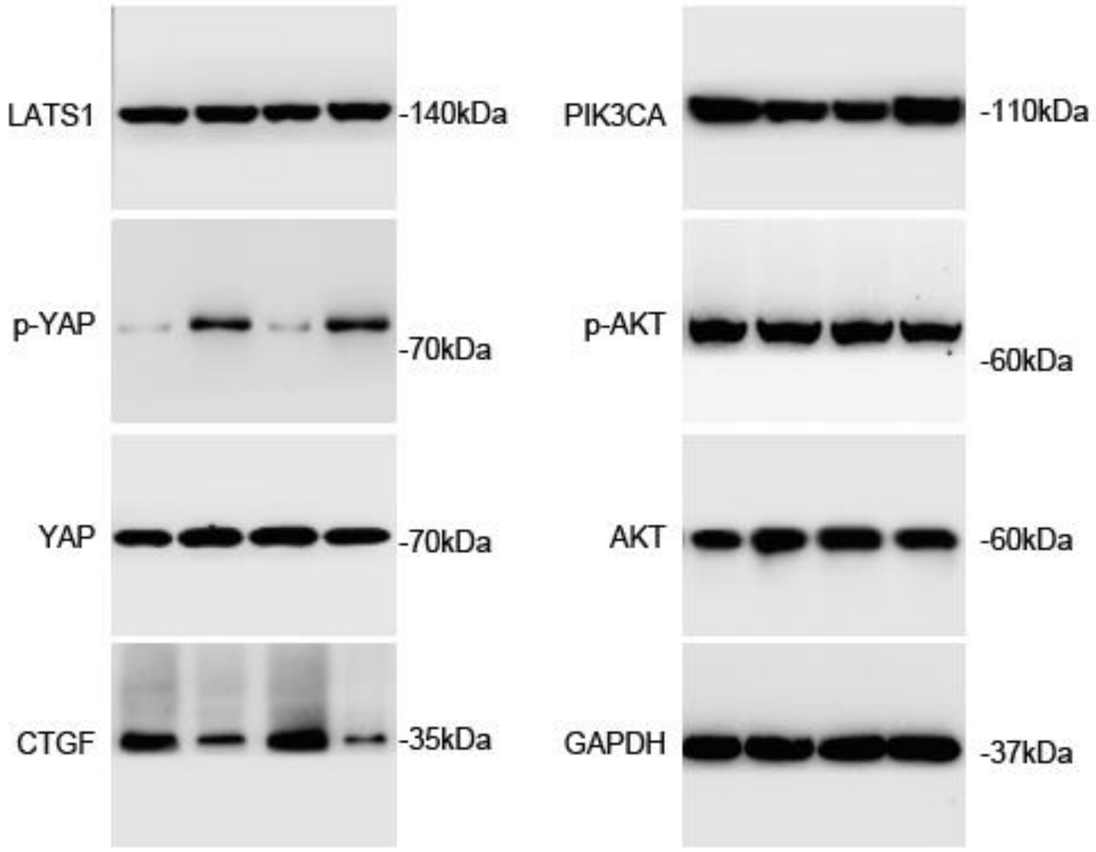
LATS1
-140kDa
p-YAP
-70kDa
YAP
-70kDa
CTGF
-35kDa
PIK3CA
-110kDa
p-AKT
-60kDa
AKT
-60kDa
GAPDH
-37kDa

Immunoblot images depicted in Fig. 4a

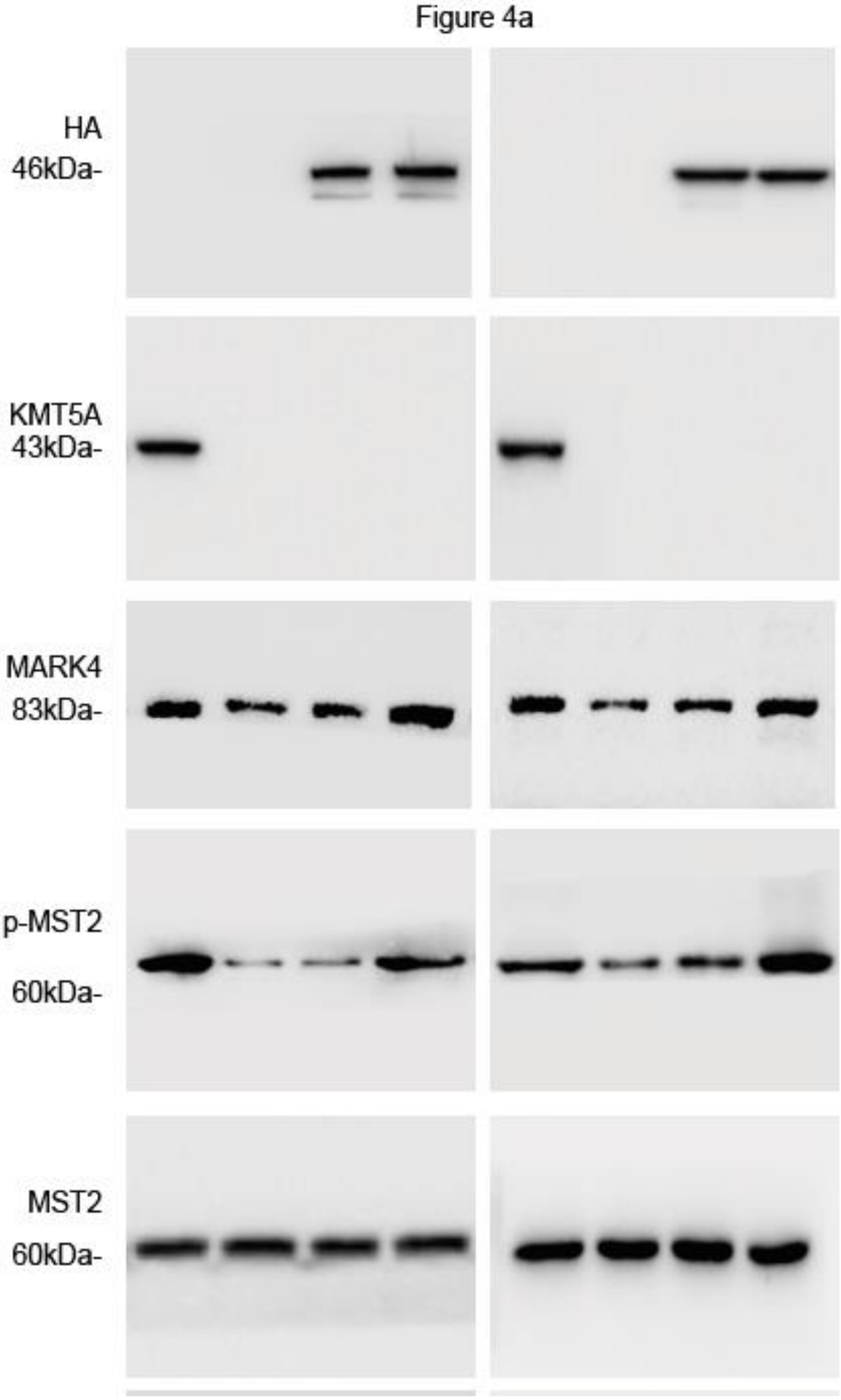

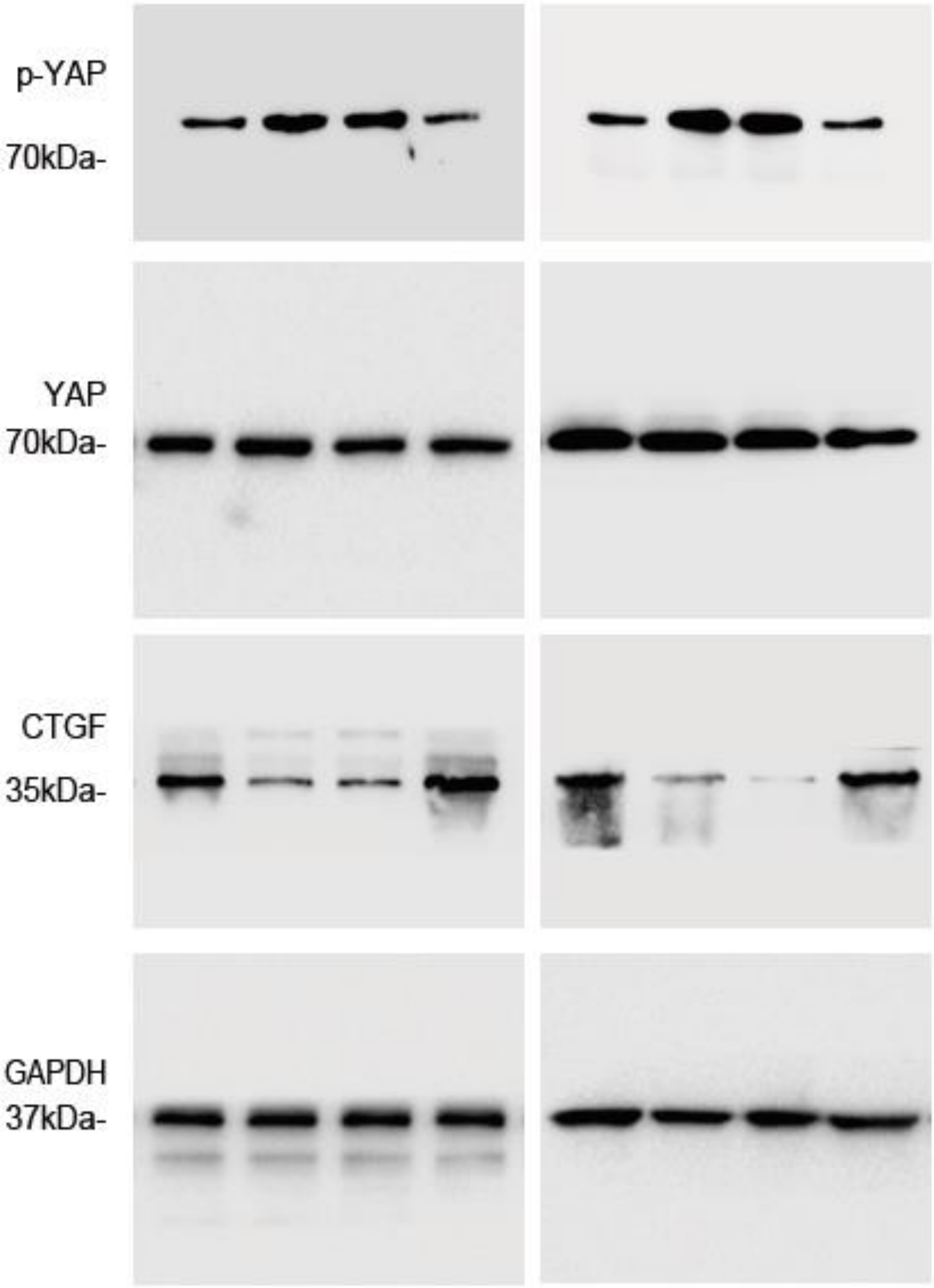
p-YAP
70kDa-
YAP
70kDa-
CTGF
35kDa-
GAPDH
37kDa-

Immunoblot images depicted in Fig. 5b, 5c and 5d

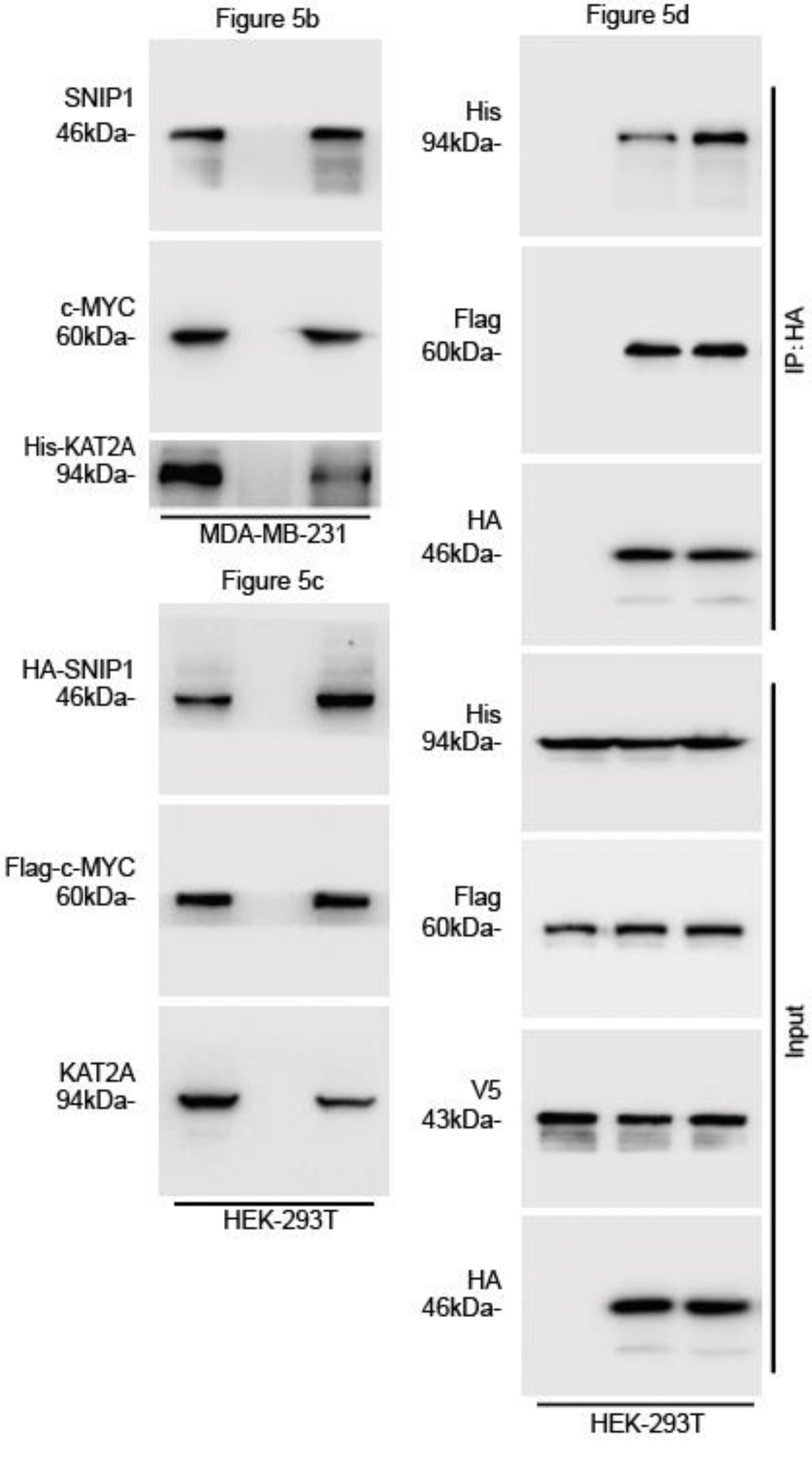

Immunoblot images depicted in Fig. 5e and 5f

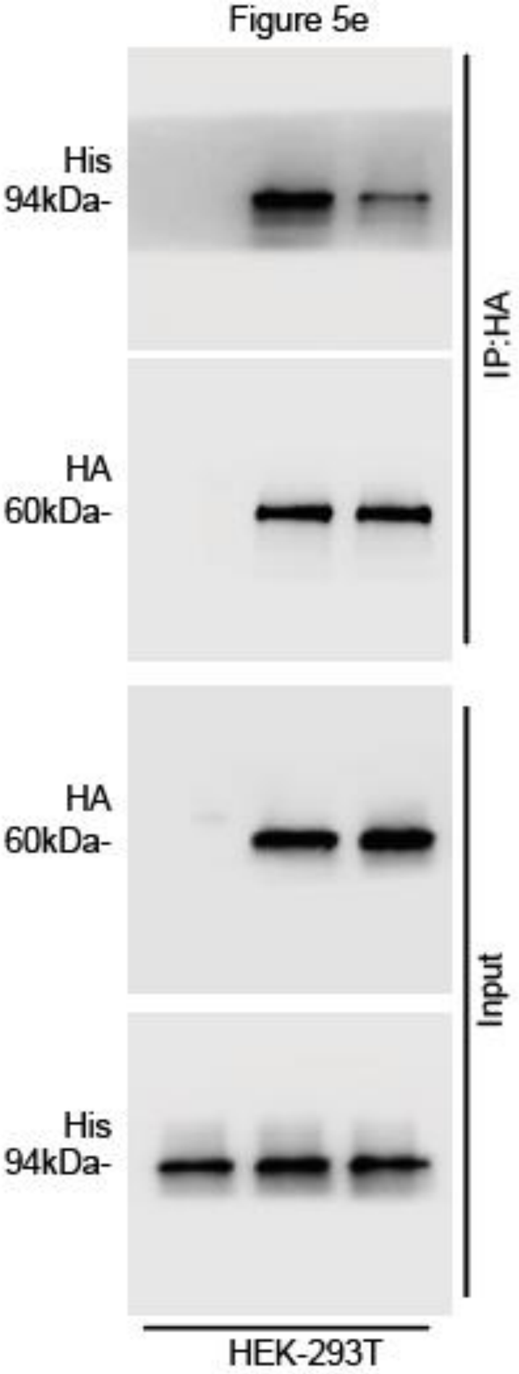

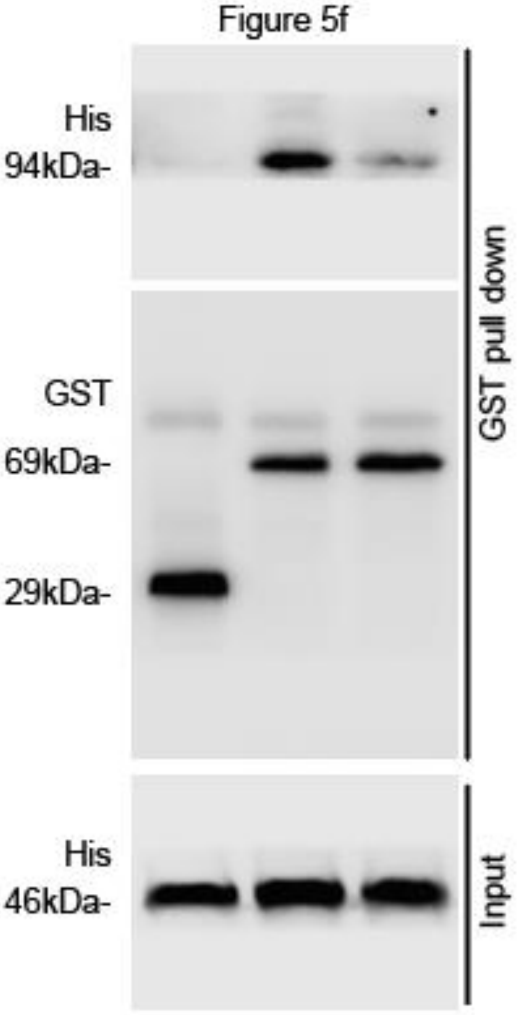

Immunoblot images depicted in Fig. 6a and 6b

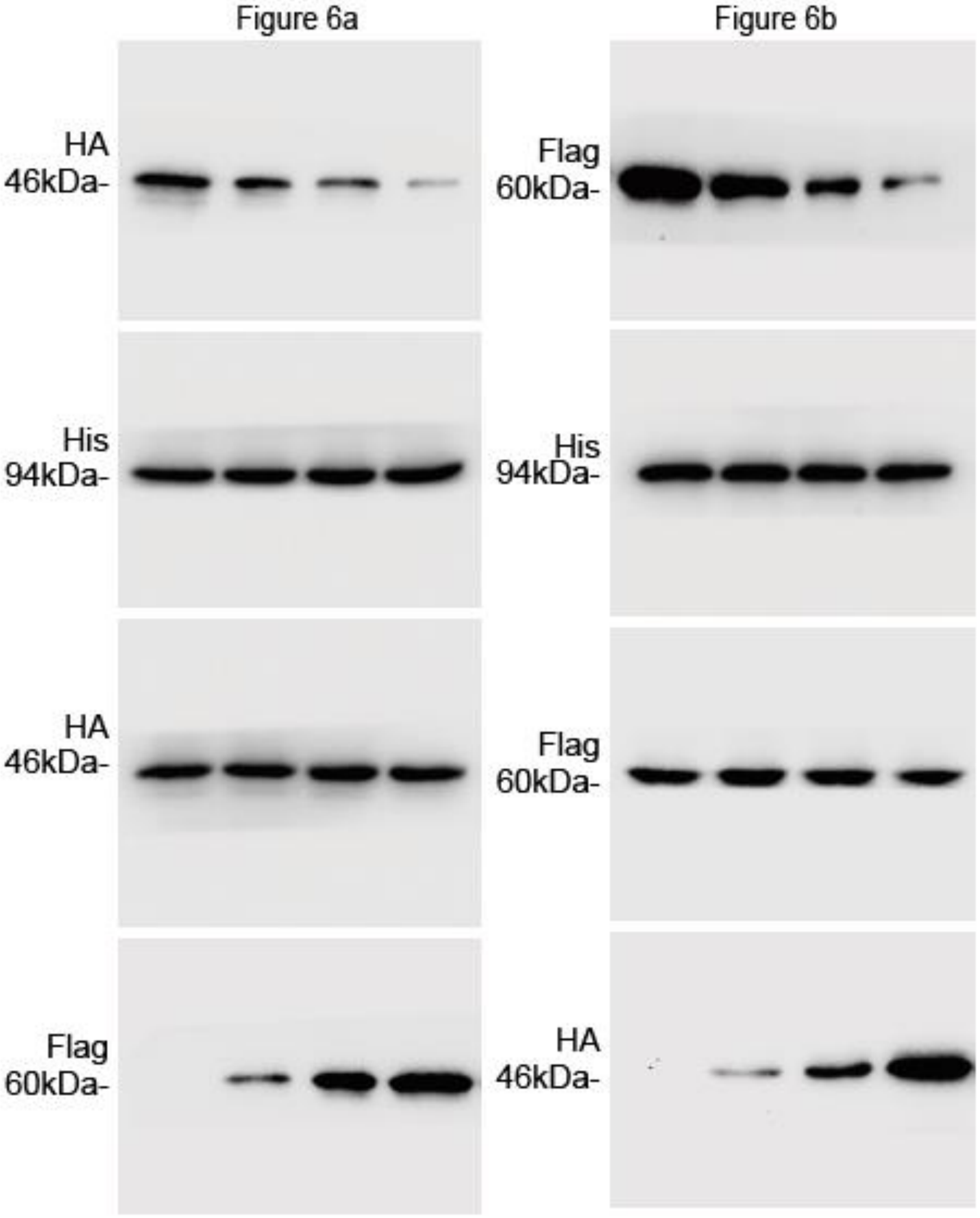

Immunoblot images depicted in Fig. 6c

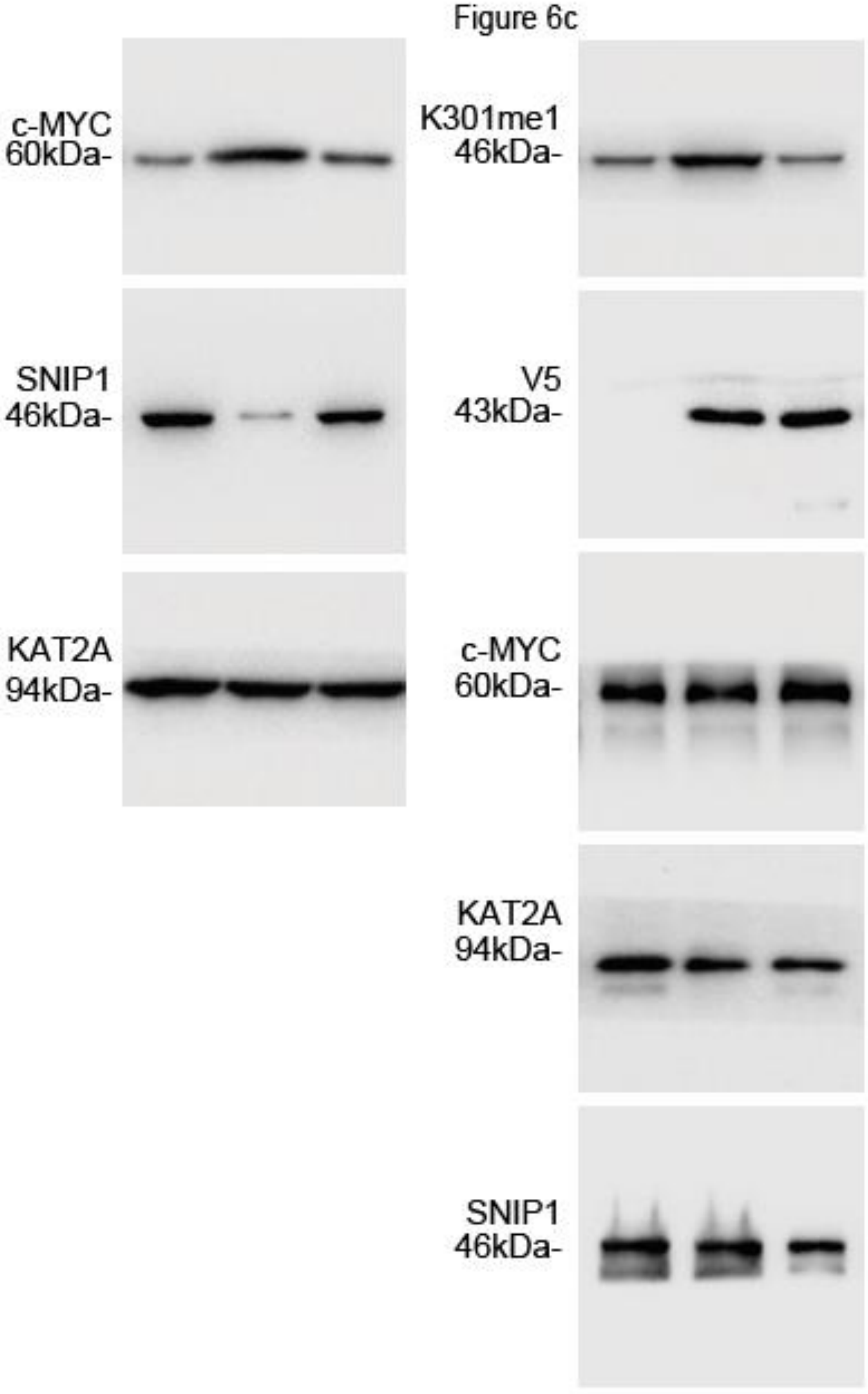

Immunoblot images depicted in Fig. 6d

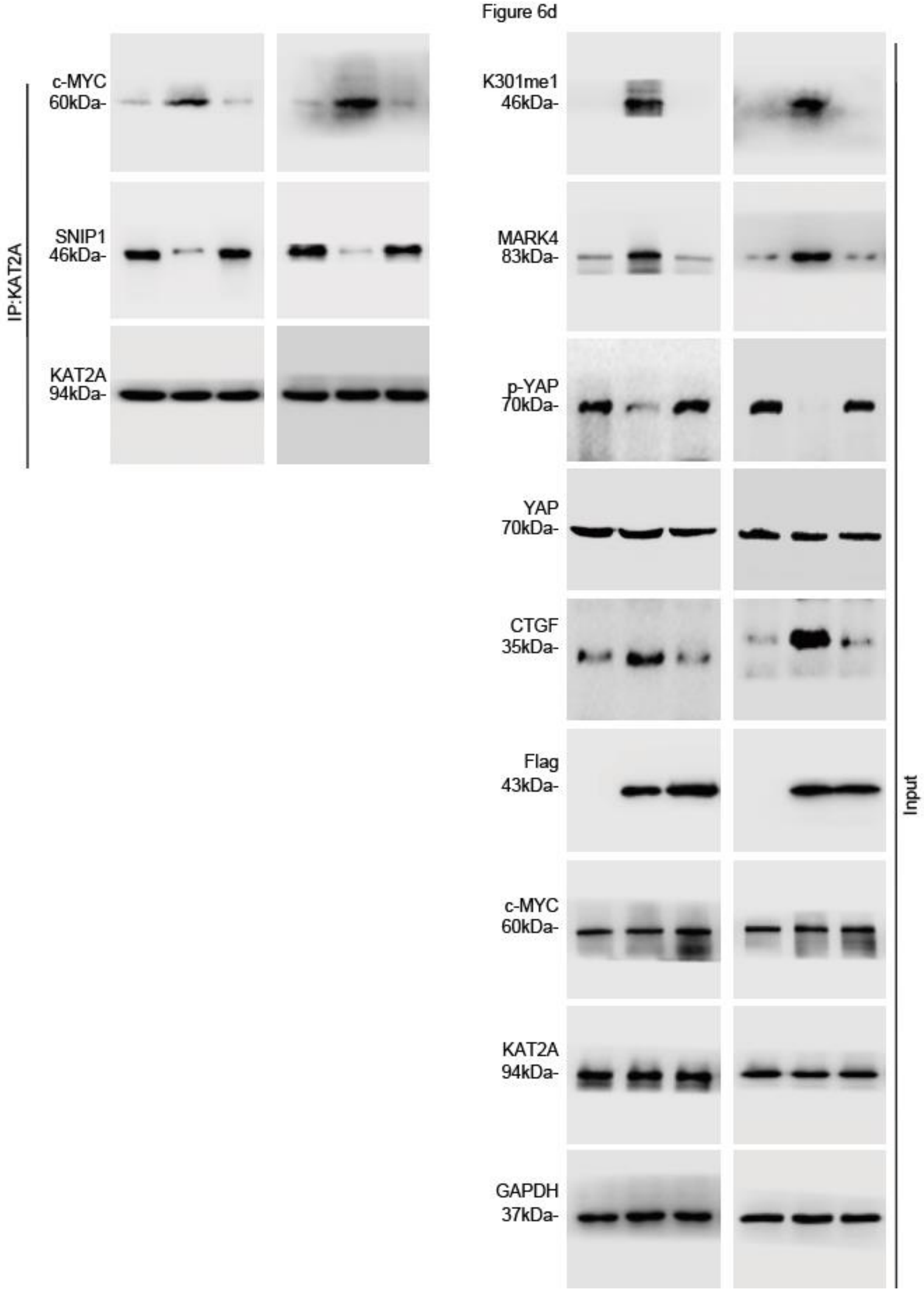

Immunoblot images depicted in Fig. 8d

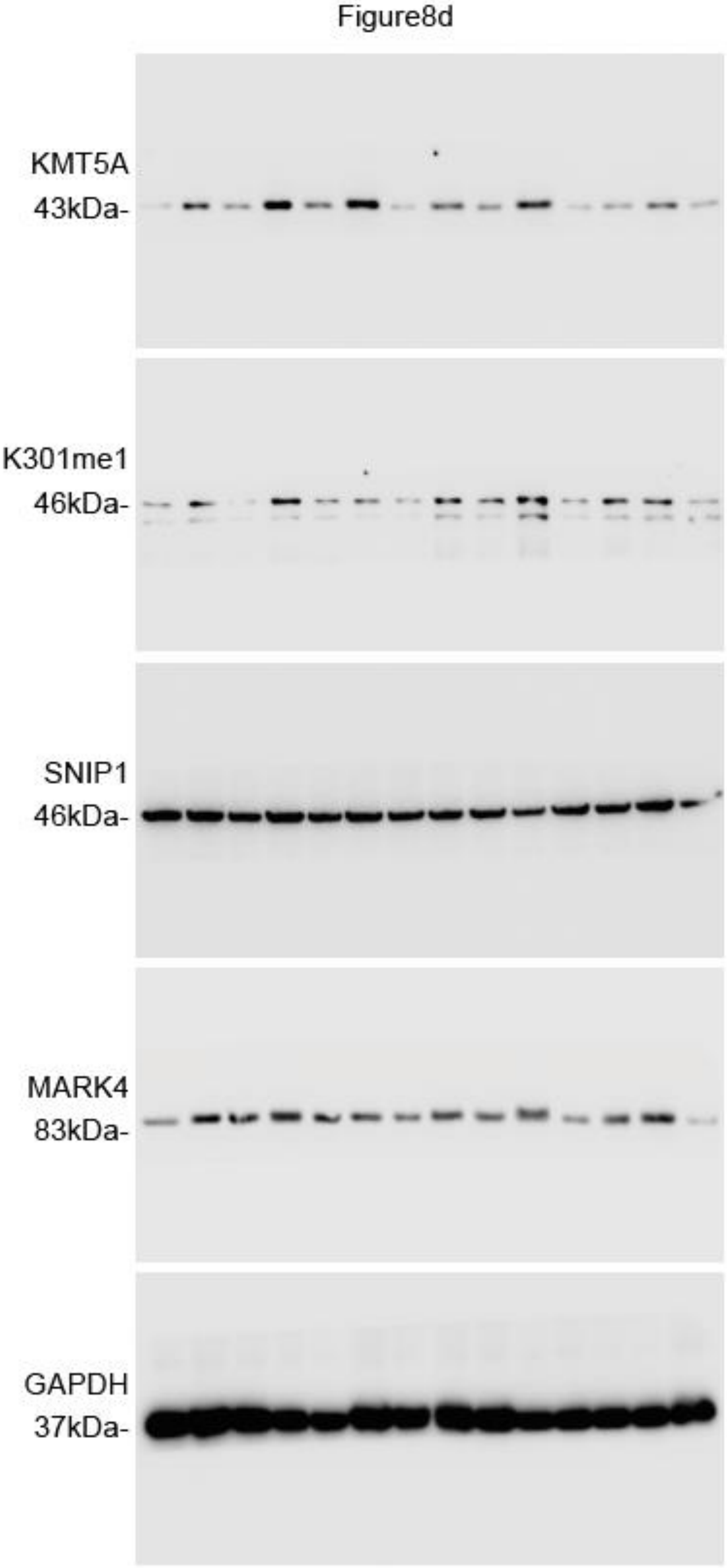

Immunoblot images depicted in Fig. S1a, S1c, and S1d

Figure S1a

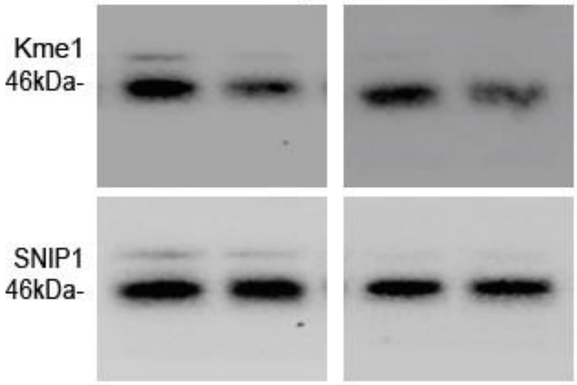

Figure S1c

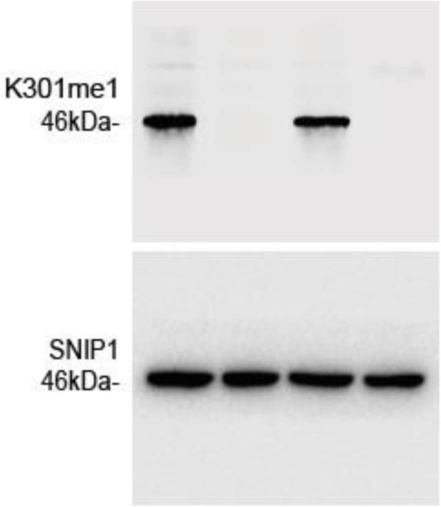

Figure S1d

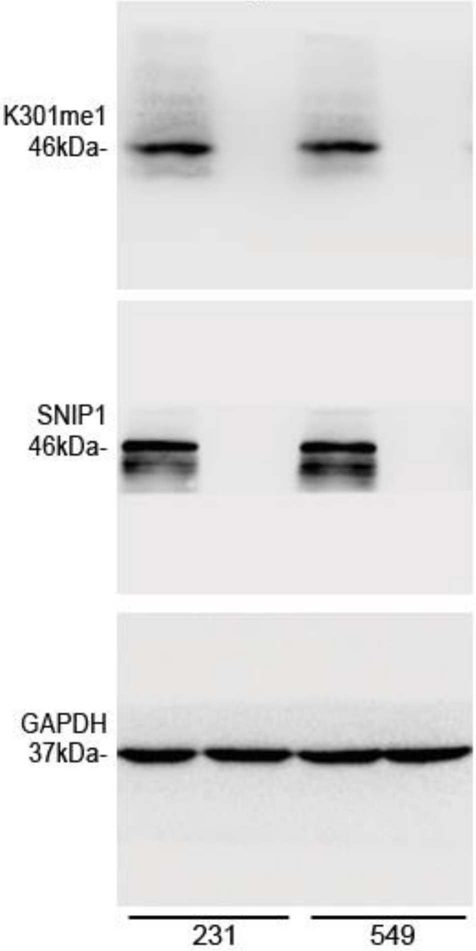

Immunoblot images depicted in Fig. S1e and S1g

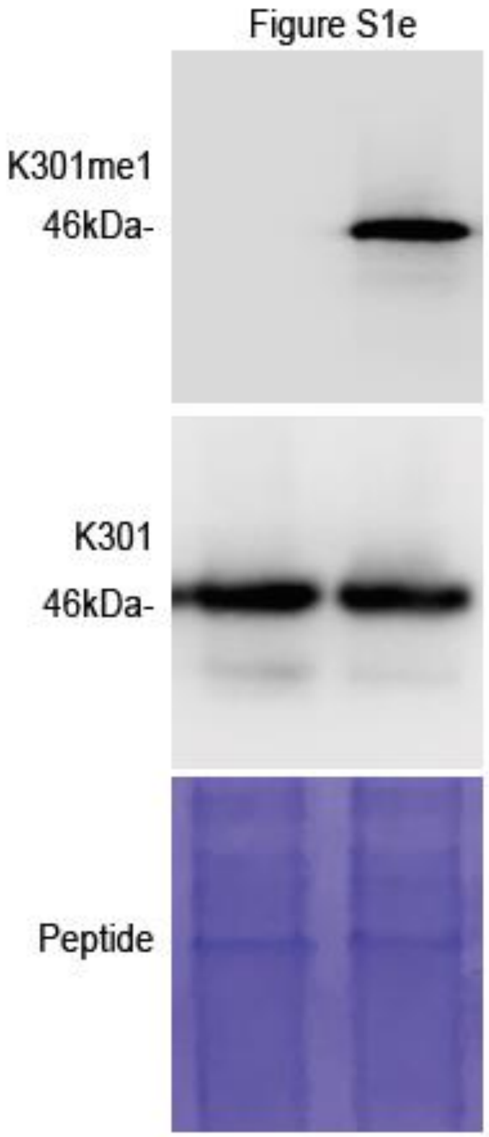

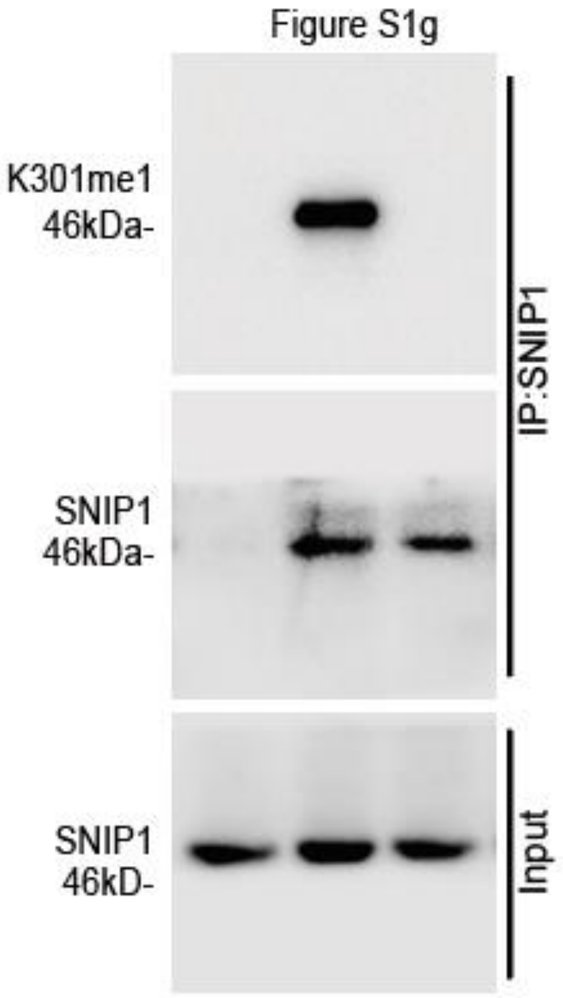

Immunoblot images depicted in Fig. S2a and S2b

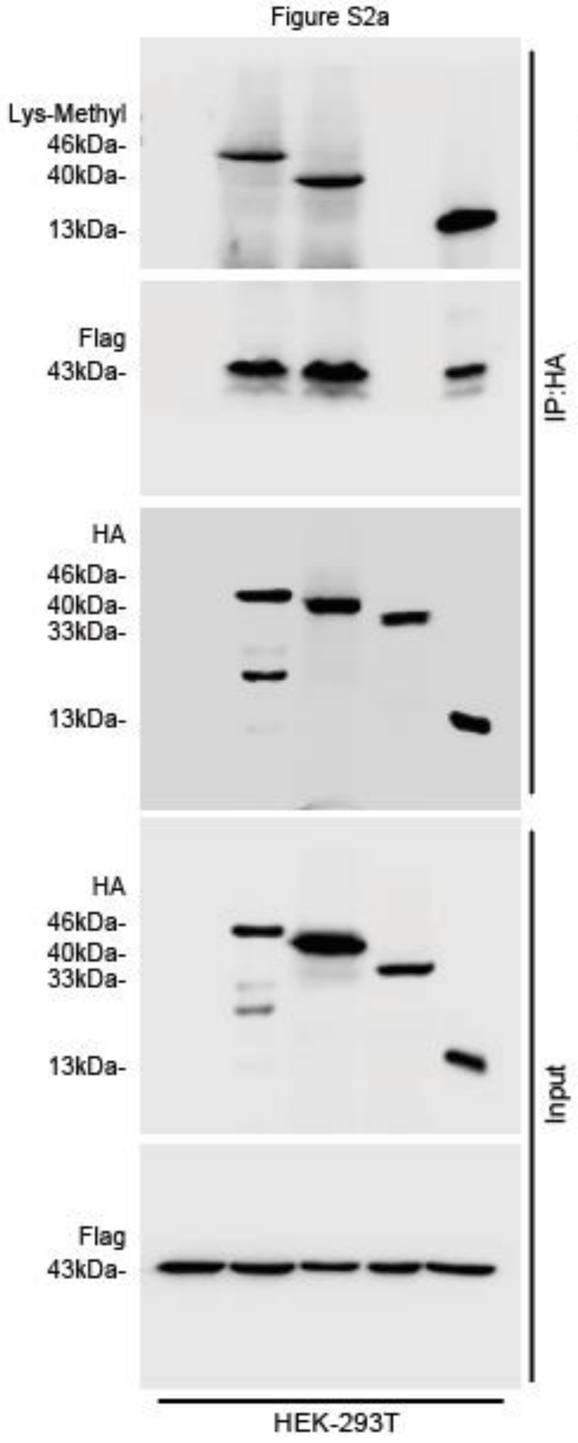

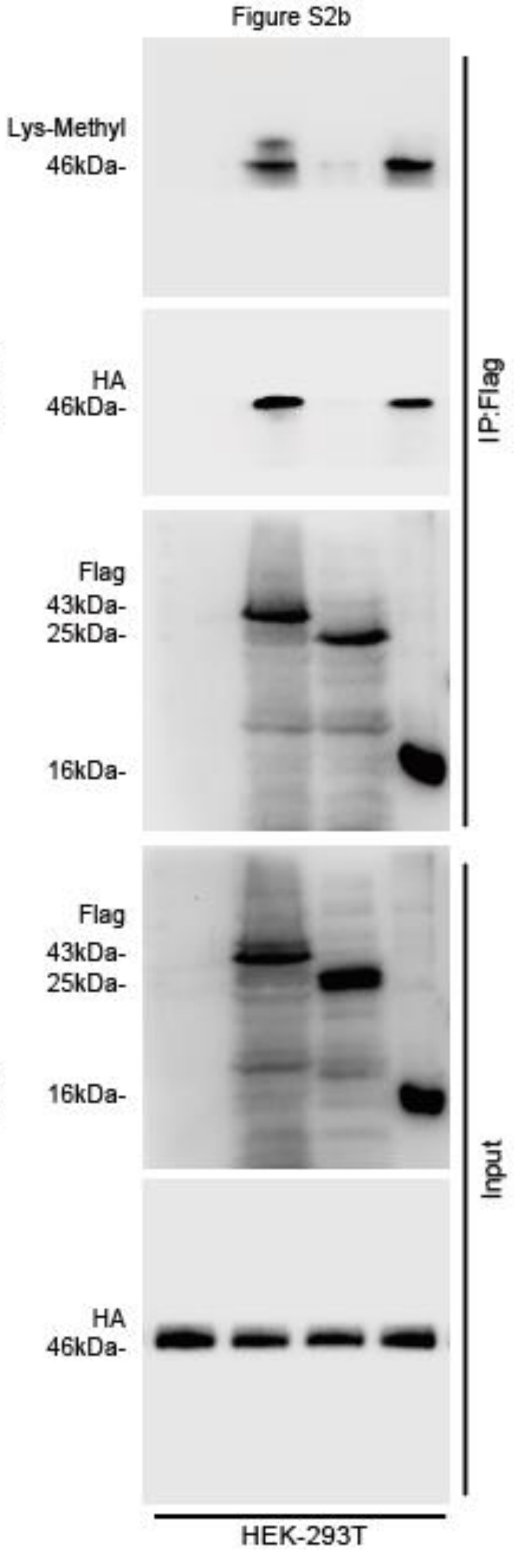

Immunoblot images depicted in Fig. S3d

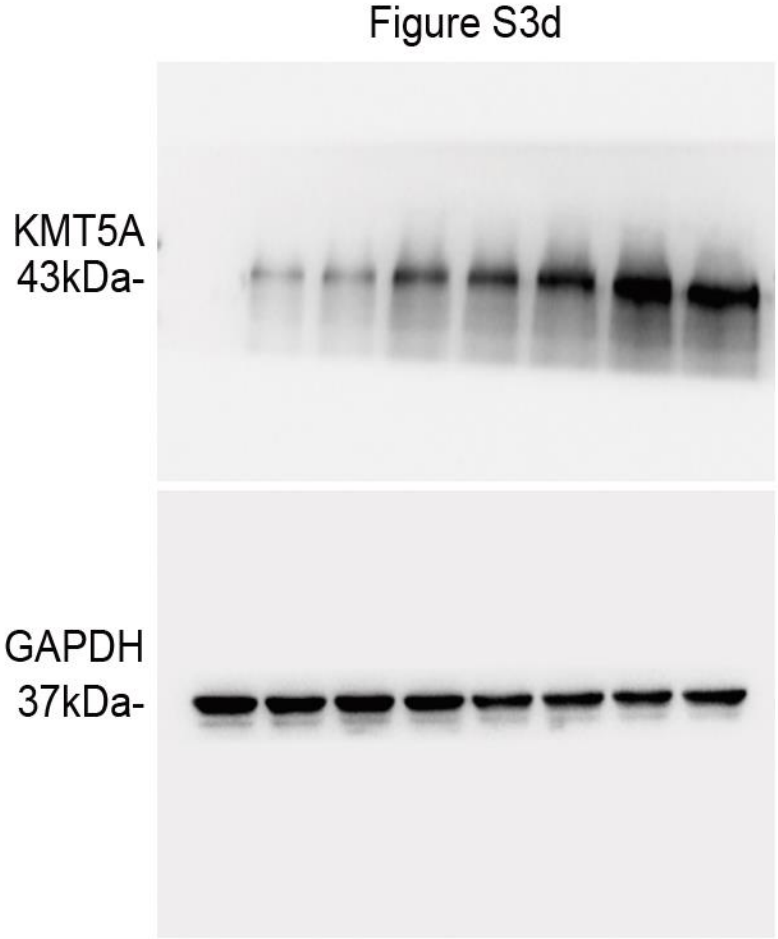

Immunoblot images depicted in Fig. S4a

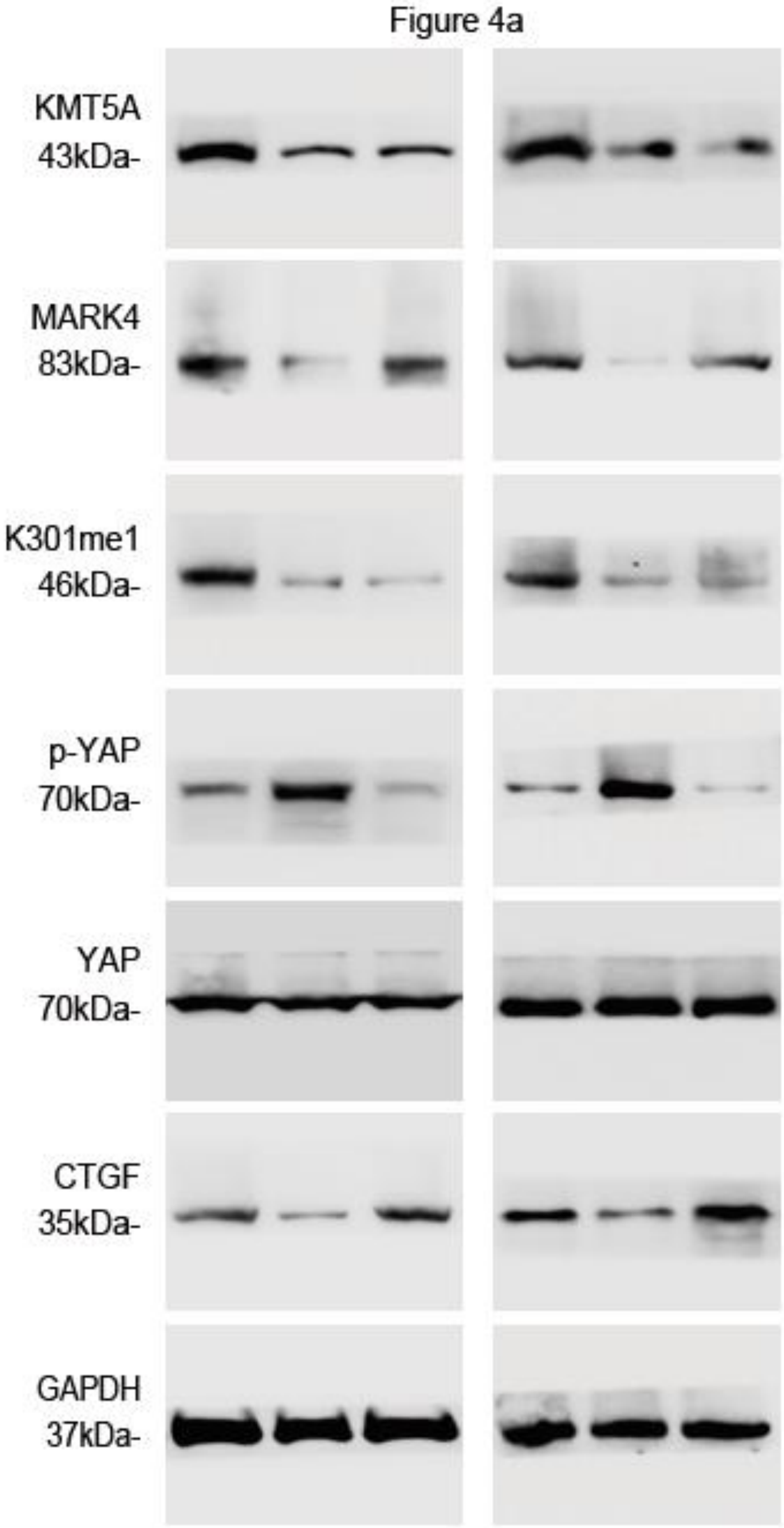

Immunoblot images depicted in Fig. S5a

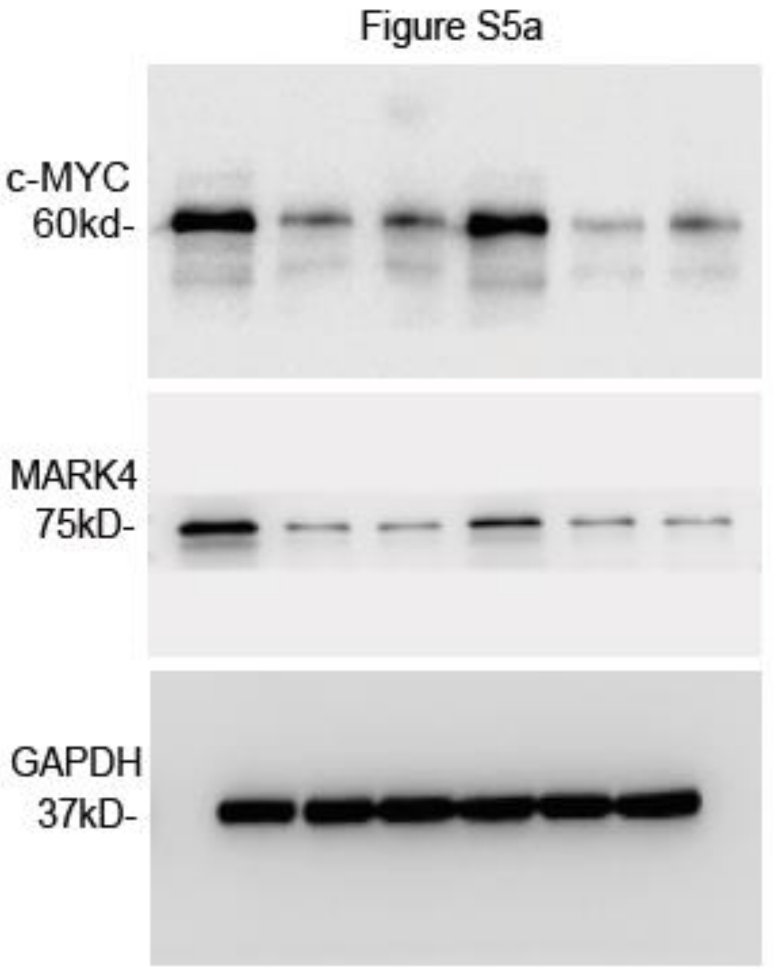

Immunoblot images depicted in Fig. S6a

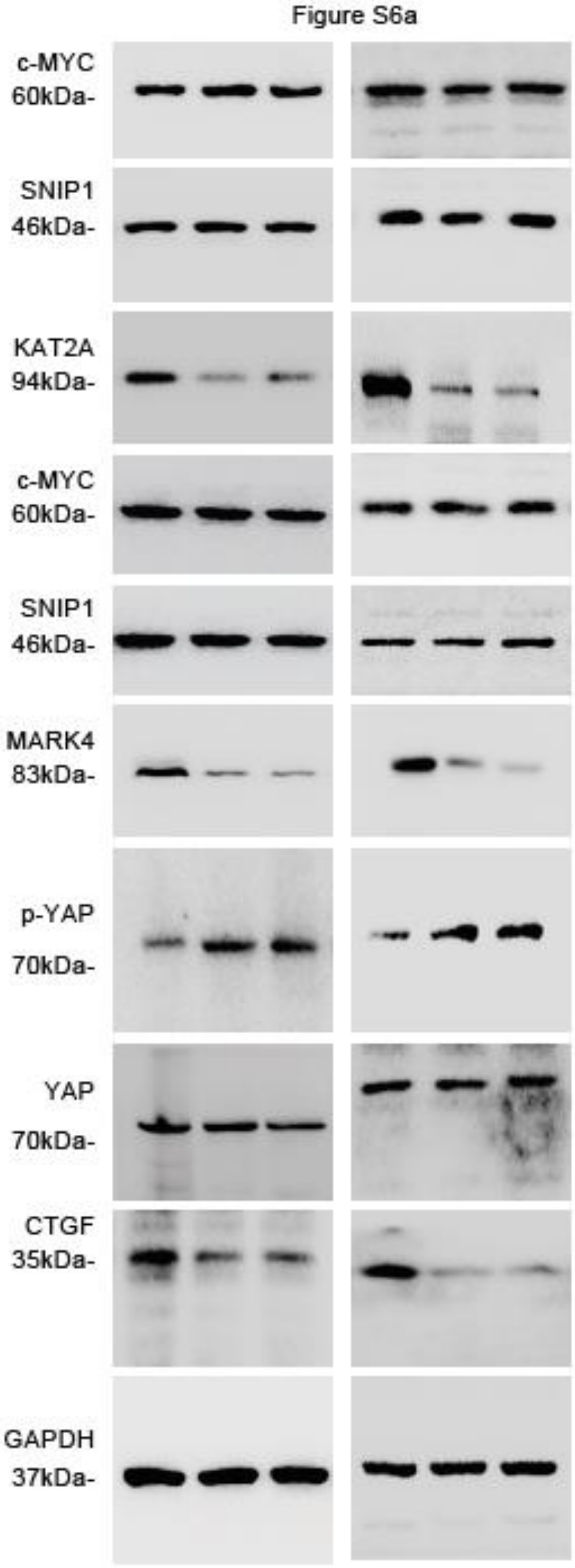

Supplement: Supplementary file 5 — Source Data [file 41467_2022_29899_MOESM5_ESM.zip › Source Data files/Uncropped gel images.pdf]
